# Deep computational image analysis of immune cell niches reveals treatment-specific outcome associations in lung cancer

**DOI:** 10.1038/s41698-023-00403-x

**Published:** 2023-06-01

**Authors:** Cristian Barrera, Germán Corredor, Vidya Sankar Viswanathan, Ruiwen Ding, Paula Toro, Pingfu Fu, Christina Buzzy, Cheng Lu, Priya Velu, Philipp Zens, Sabina Berezowska, Merzu Belete, David Balli, Han Chang, Vipul Baxi, Konstantinos Syrigos, David L. Rimm, Vamsidhar Velcheti, Kurt Schalper, Eduardo Romero, Anant Madabhushi

**Affiliations:** 1 Department of Biomedical Engineering, School of Medicine, Emory University, Atlanta, GA USA; 2 Louis Stokes Cleveland VA Medical Center, Cleveland, OH USA; 3 Case Western Reserve University, School of Engineering, Cleveland, OH USA; 4 Cleveland Clinic, Cleveland, OH USA; 5 Department of Population and Quantitative Health Sciences, Case Western Reserve University, Cleveland, OH USA; 6 Weill Cornell Medical College, New York, NY USA; 7 Institute of Pathology, University of Bern, Bern, Switzerland; 8 Graduate School for Health Sciences, University of Bern, Bern, Switzerland; 9 Department of Laboratory Medicine and Pathology, Institute of Pathology, Lausanne University Hospital and University of Lausanne, Lausanne, Switzerland; 10 Bristol Myers Squibb, New York, NY USA; 11 School of Medicine, National and Kapodistrian University of Athens, Athens, Greece; 12 School of Medicine, Yale University, New Haven, CT USA; 13 New York University, New York, NY USA; 14 Universidad Nacional de Colombia, Facultad de Medicina, Bogotá, Colombia; 15 VA Medical Center, Atlanta, OH USA

**Keywords:** Prognostic markers, Mathematics and computing, Cancer microenvironment, Cancer imaging

## Abstract

The tumor immune composition influences prognosis and treatment sensitivity in lung cancer. The presence of effective adaptive immune responses is associated with increased clinical benefit after immune checkpoint blockers. Conversely, immunotherapy resistance can occur as a consequence of local T-cell exhaustion/dysfunction and upregulation of immunosuppressive signals and regulatory cells. Consequently, merely measuring the amount of tumor-infiltrating lymphocytes (TILs) may not accurately reflect the complexity of tumor-immune interactions and T-cell functional states and may not be valuable as a treatment-specific biomarker. In this work, we investigate an immune-related biomarker (PhenoTIL) and its value in associating with treatment-specific outcomes in non-small cell lung cancer (NSCLC). PhenoTIL is a novel computational pathology approach that uses machine learning to capture spatial interplay and infer functional features of immune cell niches associated with tumor rejection and patient outcomes. PhenoTIL’s advantage is the computational characterization of the tumor immune microenvironment extracted from H&E-stained preparations. Association with clinical outcome and major non-small cell lung cancer (NSCLC) histology variants was studied in baseline tumor specimens from 1,774 lung cancer patients treated with immunotherapy and/or chemotherapy, including the clinical trial Checkmate 057 (NCT01673867).

## Introduction

Adaptive immune responses to cancer include the local accumulation of tumor-infiltrating lymphocytes (TILs) at the tumor site comprising both B-cell and T-cell subsets. The interaction of these cells occurs within lymphoid organs as well as at sites of inflammation and tumor microenvironment; often, these structures are referred to as immune cell niches^1,2^. These TILs can initiate, recognize, and destroy tumor cells thus propagating and maintaining anti-cancer immune responses^3,4^. Despite increasing evidence in the clinical scenarios and the novel design of combination of different therapy strategies (e.g., chemotherapy + immunotherapy), there is a need to understand the effect of said therapies on the tumor microenvironment^5^. For instance, immunotherapy-based treatment has yielded limited response rates and unclear underlying biological mechanisms^6^, a phenomenon also observed in the context of chemotherapy^7^. To date, there continue to be gaps in the comprehensive and systematic characterization of the tumor milieu, limiting our ability to predict treatment response for chemotherapy and immunotherapy.

The challenges deepen in the context of tumor-immune characterization and association with treatment response between tumor histology sub-types such as adenocarcinoma (AD) and squamous cell carcinoma (SCC). These differences are likely on account of the tumor immune milieu, which is extremely diverse on account of unique and distinct cellular phenotypes (immune deserts, immune-excluded, and inflamed tumors)^8^. Further, the presence of specific immune cells such as cancer-specific T-cells is required for effective anti-cancer immunity. Yet, evidence indicates that only a small fraction of T-cells is cancer-specific with most acting as ‘bystanders’ (non-tumor antigens)^9^. Others are identified as exhausted or dysfunctional and have the potential to be reinvigorated by immune checkpoint blockers^10^. These cells are not homogeneous groups but highly diverse in their specificities and effector functions.

A number of computational-based approaches have been proposed to measure the total number of TILs and their density on immunofluorescence (IF) images to assess the association of these measures with prognosis and response to therapy^11,12^. However, the preparation procedures not only requires a fluorescence microscope, but the specific antibodies and fluorochromes preparation can take up to 5 h (Leica Microsystems- Immunofluorescence protocol), compared to H&E (Hematoxylin & eosin) which is performed in minutes. Further, these approaches tend to be very expensive, complex, and involve tissue destruction. On the other hand, studies using H&E images use computational enumeration of TIL count and density^12,13^. Other works^14–17^ have employed spatial arrangement-based approaches to capture the spatial patterns of TILs on H&E tissue images, with some studies showing association with response to treatment;^15,18^ however, these approaches have considered TILs as a single entity, which assumes that all the TILs contribute equally to the outcome. This assumption has been refuted by several studies^11,19^ that have identified activated immune cells (as opposed to exhausted T-cells or bystander immune cells) as being responsible for engendering an active response to therapies such as chemotherapies or immune checkpoint inhibitors (ICI) blockade. This is only possible on the IF scope, due to the difficulty in identifying subtypes of immunes cells on H&E.

**Fig. 1:**
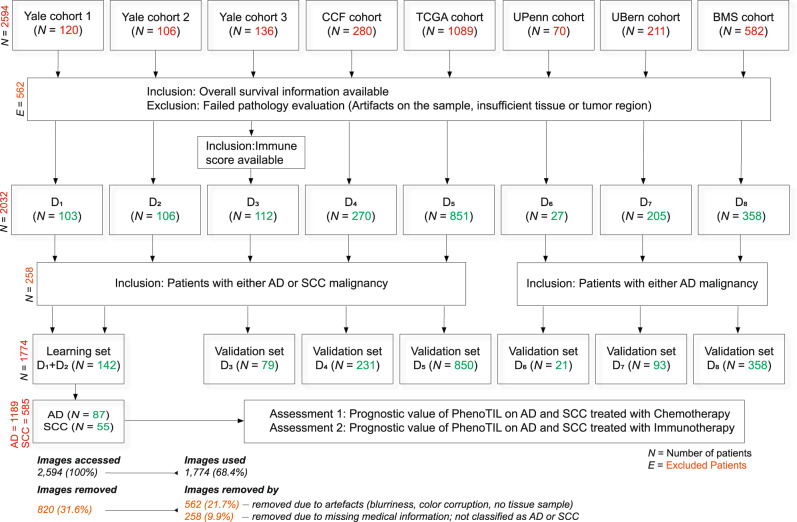
Detailed patient selection and exclusion criteria from the different NSCLC cohorts (D_1-8_). The inclusion criteria for the study. In total 1774 patients who satisfied all the inclusion criteria and who did not meet any of the exclusion criteria were identified. Images with low quality, blurry effects, and significant artifacts were considered for all the datasets, and images that presented them were excluded from the analysis. For D_6_, D_7,_ and D_8_ the additional inclusion criteria invoked included the availability of histologic subtype AD. Inclusion criteria for D_7_ were as follows: from the initial 211 patients, those patients were considered who either underwent surgical resection after neoadjuvant therapy or had a primary resection at a locally advanced stage, which qualified them for neoadjuvant therapy. Three patients had more than one WSI scanned due to the following reasons: Firstly, the patient had a sample with neoadjuvant lung SCC with small AD as an incidental finding, second was a patient with neoadjuvant adenosquamous carcinoma and the third patient had a primary lung AD with three regions with quite different growth patterns within the primary. After invoking the inclusion and exclusion criteria for this study, 71 cases for D_1_ (49 excluded), 71 for D_2_ (35 excluded), 79 for D_3_ (57 excluded), 231 for D_4_ (49 excluded), 850 for D_5_ (239 excluded), 21 for D_6_ (49 excluded), 93 for D_7_ (118 excluded) and 358 for D_8_ (224 excluded) were included.

**Fig. 2:**
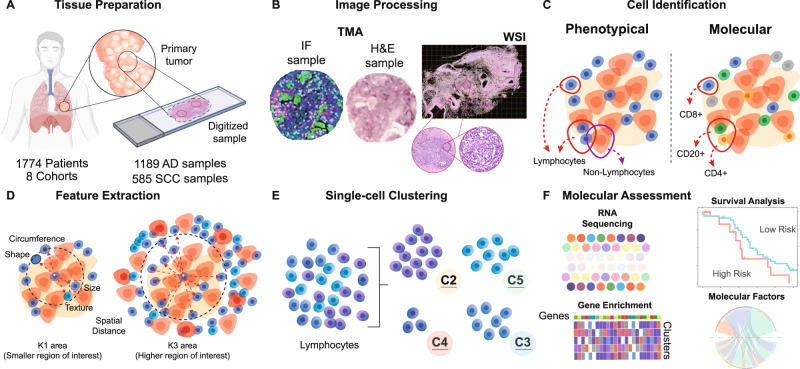
Workflow of the data preparation and experiments. **A** *Tissue preparation:* The cohorts were digitized and represented in the form of patches extracted from whole slide images (WSIs) and tissue microarray (TMA) punches. **B** *Image preprocessing:* A subset composed of H&E-stained TMA and corresponding immunofluorescence (IF) images were utilized to analyze tumor-infiltrating lymphocyte (TIL) subtypes. **C** *Cell Identification:* Corresponding TILs from the H&E samples were associated with IF molecular labels (CD4^+^, CD8^+^, CD20^+^). **D** *Feature extraction:* Phenotyping features were extracted from the TIL cells patches extracted from the WSI and TMAs. **E** *Single-Cell Clustering*: An unsupervised clustering approach was applied to the phenotyping features of TILs. **F** *Molecular Assessment:* RNA-seq-based transcriptome data is obtained from each WSI-TCGA sample. TIL clusters were used as the input matrix to build a model that associate with the clinical outcome overall survival, using a Cox proportional hazards regression model with elastic net regularization. Associations between cluster conformation, molecular, morphological and genomics composition were studied. Minor components of the figure were obtained from BioRender.

In this study, we present PhenoTIL, a computational pathology approach that utilizes H&E-stained images to first identify immune cell niches, and subsequently capture quantitative metrics relating to the spatial interplay of TILs and cancer cells within these immune cell niches. PhenoTIL assumes that the TILs within the tumor microenvironment have different roles in relation to the outcome of the patients and enable characterization of patient risk based off composition of the constituent clusters. The quantitative PhenoTIL metrics describe each of the TILs in reference to their color, shape, texture and convey spatial architectural information about the neighboring TILs and non-TILs at different length scales. The main hypothesis is aimed to investigate the value of PhenoTIL as the immune-related biomarker for treatment-specific outcomes in NSCLC. An important factor of PhenoTIL is the ability to identify distinct TIL clusters on H&E images that bear a resemblance to activated immune ‘hotspots’ associated with tumor rejection or immune ‘cold spots’ associated with dysfunctional features and contributing to adverse outcomes. We evaluated this approach in its ability to prognosticate overall survival (OS) on pre-treatment H&E-stained samples of 1774 patients with lung AD (*n* = 1189) and SCC (*n* = 585) treated with different types of chemotherapy and immunotherapy agents (Fig. 1); the study also included patients from a completed clinical trial of immunotherapy in lung AD patients (Checkmate 057)^20^. Kaplan–Meier (KM) survival curves were generated to evaluate the association of the phenoTIL signature with outcome. The PhenoTIL signature was also evaluated in terms of the molecular composition of immune cell subtypes (Fig. 2) by identifying single cells molecularly using co-registered quantitative immunofluorescence image (QIF) information on a subset of H&E images. To identify which biological pathways were associated with the PhenoTIL signature, gene-set enrichment analysis (GSEA) was performed. Separate analyses were carried out for AD and SCC cases considering their documented differences in their pathophysiology, clinical features, immunogenicity, prognosis, and treatment sensitivity^21–25^.

## Results

### Description of patients cohorts

Eight datasets (D_1_–D_8_) were used in this multi-institutional study, including seven retrospective institutional cohorts and one from a completed clinical trial. Figure 1 illustrates the inclusion and exclusion criteria for patient selection. Out of the 2594 patients available, a total of 1774 (68.4%) patients, who satisfied all the inclusion criteria and did not meet any of the exclusion criteria, were incorporated into this study. A total of 820 (31.6%) patients were excluded from the study due to various reasons related to the image samples. A total of 562 (21.7%) images were removed due to insufficient quality (faulty image format, artifacts such as complete image blurriness, color corruption, no tissue sample available, etc.). In addition, 258 (9.9%) images were removed due to missing medical information or were not classified as either adenocarcinoma or squamous cell carcinoma. The quality control was performed utilizing HistoQC^26^. The first three datasets (D_1_–D_3_) consisted of pre-treatment formalin-fixed paraffin-embedded tumor sections from 44 AD and 27 SCC patients (D_1_), 43 AD and 28 SCC patients (D_2_), and 58 AD and 21 SCC patients (D_3_) provided by the Department of Pathology at Yale University (D_2_ were collected at Sotiria General Hospital and Patras University General Hospital at Greece, but were made available from Yale Pathology)^12^. Corresponding QIF images from D_3_ had been previously acquired and prepared via pan-cytokeratin staining, CD4^+^, CD8^+^, and CD20^+^ using the sequential multiplexed immunofluorescence protocol (See an example in the Supplementary fig. 1(A)), with isotype-specific primary antibodies being employed for staining T lymphocytes (CD3+ IgG, 1:100, clone E272, Novus biologicals, CO), cytotoxic T-cells (CD8 + IgG1, 1:250, clone C8 + /144B, DAKO), and B lymphocytes (CD20 + IgG2a, 1:150, clone L26, DAKO)^12^. D_4_ comprised 145 AD and 86 SCC patients obtained from the Cleveland Clinic (CCF). D_1_-D_3_ were represented in tissue microarrays (TMAs) with cores of size 0.6 mm from each paraffin block (digitally scanned at 20×). D_4_ was represented in TMAs with cores of size 1.2 mm from each paraffin blocks (digitally scanned at 40×)^27^. D_5_ comprised whole slide images (WSIs) of pre-treatment H&E-stained pathology slides (scanned at 40×) of 427 AD and 423 SCC patients (D_5_) obtained from The Cancer Genome Atlas (TCGA). D_6_ comprised diagnostic WSI samples (scanned at 40×) of 21 AD patients who had received ICI-based immunotherapy at the University of Pennsylvania Hospital (UPenn).

D_7_ (acquired at 20× resolution; ratio of 0.2431 μm/px) comprised 93 AD patients (from a larger cohort of 211 patients with 215 available WSIs); these patients had no neuroendocrine histology and had a sufficient amount of primary tumor. A subset of 43 patients (out of the 93) were treated with chemotherapy prior to resection with neoadjuvant intention. 50 patients (out of the 93) were primary resected LUAD with pathologically confirmed infiltration of lymph nodes of at least the mediastinal level. The patients which were neoadjuvantly treated, received platinum-based chemotherapy combinations. Ultimately, slides corresponding to 93 patients were included, along with corresponding tumoral programmed cell death 1 ligand 1 (PD-L1) expression values assessed on the resection samples, for these patients^28,29^. PD-L1 expression was assessed by an expert pathologist as the tumor proportional score (TPS), i.e., the proportion of PD-L1 positive tumor cells to all tumor cells. PD-L1 positive tumor cells were defined as showing membranous staining of any intensity. TPS was assessed as a continuous parameter (1% increments up to 10 and 5% increments in cases showing >10% expression). For statistical analyses, each patient was categorized as high or low expression based on the intensity of immunohistochemistry staining of the lung resection samples. Patients with over 50% PD-L1 expression were classified as high PD-L1 and patients with less than 50% expression were classified as low PD-L1. The data was provided by the University of Bern in Switzerland (UBern).

D_8_ comprised WSIs (scanned at 20×) of 358 AD patients provided by Bristol-Myers Squibb (BMS), the clinical trial CA209-057^16^ (ClinicalTrials.gov identifier: NCT01673867). The patients from D_8_ were randomized from an open-label phase-3 study (international) of non-squamous non-small cell lung cancer (NSCLC), also referred to as CheckMate 057. Patients whose lung cancer progressed during or after a platinum-based doublet chemotherapy regimen received nivolumab or docetaxel. The actual enrollment for the study was 782 participants from 115 international study locations^16^. A total of 204 patients received nivolumab (at a dose of 3 mg per kilogram of body weight every 2 weeks) and 154 patients received docetaxel (at a dose of 75 mg per square meter of body-surface area every 3 weeks). The primary endpoint was OS. The validation on this dataset was carried out in a blinded fashion in which we had access to whole slide images but not to clinical data. Our algorithms were applied to the images to predict risk scores and patient labels, and then these values were sent to the BMS team, who carried out the statistical analysis and provided us with the corresponding results.

Table 1 provide a summary of the datasets and images employed in this study along with the corresponding clinicopathology and outcome information (Supplementary Table 1 shows the clinicopathology information in more detail for each cohort).

**Table 1:** Summary of the clinical and pathologic information for the whole datasets and treatment information for the cohorts involved.

	Adenocarcinoma (ad) (*n* = 1189)	Squamous cell carcinoma (scc) (*n* = 585)	All (*n* = 1774)
*Dataset*			
D1 (Yale)	44 (3.7%)	27 (4.6%)	71 (4.0%)
D2 (Yale)	43 (3.6%)	28 (4.8%)	71 (4.0%)
D3 (Yale)	58 (4.9%)	21 (3.6%)	79 (4.5%)
D4 (CCF)	145 (12.2%)	86 (14.7%)	231 (13.0%)
D5 (TCGA)	427 (35.9%)	423 (72.3%)	850 (47.9%)
D6 (UPenn)	21 (1.8%)	0 (0%)	21 (1.2%)
D7 (UBern)	93 (7.8%)	0 (0%)	93 (5.2%)
D8 (BMS)	358 (30.1%)	0 (0%)	358 (20.2%)
*Sex*			
Female	518 (43.7%)	127 (21.7%)	645 (36.4%)
Male	471 (39.6%)	372 (63.6%)	843 (47.5%)
Missing information	200 (16.7%)	86 (14.7%)	286 (16.1%)
*Status*			
Alive	380 (32.0%)	288 (49.2%)	668 (37.7%)
Dead	451 (37.9%)	297 (50.8%)	748 (42.2%)
Missing information	358 (30.1%)	0 (0%)	358 (20.2%)
*Age (years)*			
Mean (sd)	64.6 (9.97)	67.2 (8.49)	65.7 (9.44)
Median [min, max]	65.0 [33.0, 88.0]	68.0 [39.0, 90.0]	67.0 [33.0, 90.0]
Missing information	511 (43.0%)	91 (15.6%)	602 (33.9%)
*Stage*			
I	4 (0.3%)	3 (0.5%)	7 0.4%)
IA	227 (19.1%)	133 (22.7%)	360 (20.3%)
IB	183 (15.4%)	179 (30.6%)	362 (20.4%)
II	1 (0.1%)	3 (0.5%)	4 (0.2%)
IIA	81 (6.8%)	80 (13.7%)	161 (9.1%)
IIB	102 (8.6%)	95 (16.2%)	197 (11.1%)
III	25 (2.1%)	1 (0.2%)	26 (1.5%)
IIIA	111 (9.3%)	59 (10.1%)	170 (9.6%)
IIIB	45 (3.8%)	18 (3.1%)	63 (3.6%)
IV	321 (27.0%)	6 (1.0%)	327 (18.4%)
IVA	16 (1.3%)	1 (0.2%)	17 (1.0%)
Missing information	73 (6.1%)	7 (1.2%)	80 (4.5%)
*Stage (grouped)*			
Early-stage (I/IA/IB/II/IIA/IIB)	598 (50.3%)	493 (84.3%)	1091 (61.5%)
Late-stage (III/IIIA/IIIB/IV/IVA)	518 (43.6%)	85 (14.5%)	603 (34.0%)
Missing information	73 (6.1%)	7 (1.2%)	80 (4.5%)
*Therapy*			
Chemotherapy	964 (78%)	585 (100%)	1549(85%)
Immunotherapy	225 (18%)	0 (0%)	225 (12%)
Radchemotherapy	14 (1%)	0 (0%)	14 (1%)
Radiotherapy	10 (1%)	0 (0%)	10 (1%)
Other	24 (2%)	0 (0%)	0 (0%)

Cohorts D_1_ (*n* = 71) and D_2_ (*n* = 71) were employed for feature discovery and model training as they were previously employed in studies^12,30^ that demonstrated association between TILs and patient survival.

### PhenoTIL identified immune cell niches from pretreatment H&E samples are associated with overall survival in lung adenocarcinoma and squamous cell carcinoma patients treated with chemotherapy

**Fig. 3:**
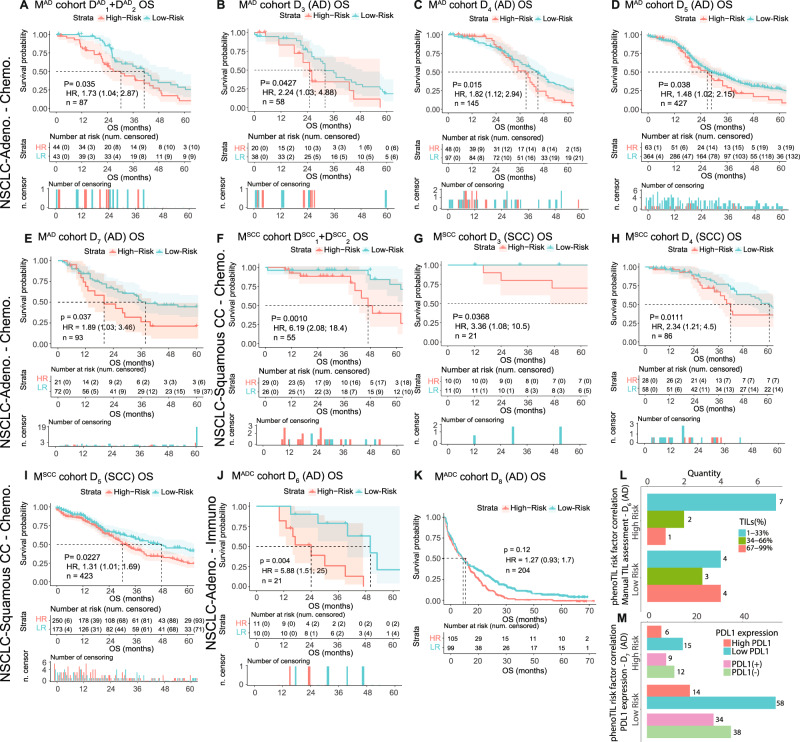
KM curves using OS as endpoint for the TIL cluster model (M^AD^). **A** Training set (D^AD^_1_ and D^AD^_2_) applied to (**B**) chemotherapy treated cohorts D_3_, (**C**) D_4_, (**D**) D_5_ and (**E**) D_7_. KM curves for the TIL cluster model (M^SCC^ (**F**) training (D^SCC^_1_ and D^SCC^_2_) set applied to (**G**) D_3_, (**H**) D_4_ and (**I**) D_5_. KM of TIL cluster model (M^AD^) applied to immunotherapy treated cohorts (**J**) D_6_ and (**K**) D_8_. **L** Bar plot displaying the correlation between the dichotomous risk groups determined by TIL cluster model (M^AD^) for cohort D_6_ on Adenocarcinoma patients and the manual assessment of percentage of TILs. The high-risk group contains 63.6% of Low TIL and the low-risk group contains 80% of High TIL content. **M** Bar plot displaying the correlation between the risk groups determined by TIL cluster model (M^AD^) on D_7_ and the PD-L1 expressions (High expression >50%, low expression ≤50%, Positive >1% and Negative expression ≤1%). The low-risk group contains 79.5% of the low PD-L1. The high-risk group contains 30% of the high PD-L1.

Figure 3(A) illustrates the Kaplan Meier (KM) survival curves corresponding to the model using the training sets (D^AD^_1_, *n* = 44 and D^AD^_2_ = 43) with cluster TIL-based measures (M^AD^). M^AD^ was statistically significantly prognostic of OS on the training sets (D^AD^_1_ and D^AD^_2_) with a hazard ratio (HR) of 1.73 (95% confidence interval (95% CI) = 1.04–2.87, *p* value (p) = 0.035, concordance-index (C-index) = 0.711 (standard error (SE) = 0.070), *n* = 87). M^AD^ was also prognostic of OS in D_3_ (*p* = 0.04, HR = 2.24, 95% CI 1.03–4.88, C-index: 0.705 (SE 0.091), *n* = 58), D_4_ (*p* = 0.015, HR = 1.82; 95% CI, 1.12–2.94, C-index: 0.689 (SE 0.051), *n* = 145), D_5_ (*p* = 0.02, HR = 5.88; 95% CI, 1.51–2.15, C-index: 0.645 (SE 0.07), *n* = 427), and D_7_ (*p* = 0.037, HR = 1.89; 95% CI, 1.03–3.46, C-index: 0.645 (SE 0.07), *n* = 93) as illustrated in Fig. 2(B)–(E). Further, the models (M^AD^ and M^SCC^) were evaluated merging all the patients from the testing cohorts (See Supplementary Fig. 3(A–B)). M^AD^ was evaluated across different lung AD stages (IA, IB, IIA, IIB, IIIA, IIIB, and IV) for patients on datasets D_3_, D_4_, D_5_, D_6_ and D_7_; results are shown in Supplementary Fig. 1(C–H) and Supplementary Fig. 2(A–F). M^AD^ yielded a statistically significant separation between low and high-risk groups (*n* = 97, *p* = 0.038, HR = 1.96, 95% CI: 1.03–3.76) in Stage III patients. For Stages IB (*n* = 127, *p* = 0.05, HR = 1.95, 95% CI: 0.99–3.83) and IIIA (*n* = 80, *p* = 0.057, HR = 1.97, 95% CI: 0.97–4.0), M^AD^ showed association with OS but did not reach statistical significance.

Figure 3(F) illustrates the KM curves corresponding to the model using the training sets (D^SCC^_1_, *n* = 27 and D^SCC^_2_ = 28) with cluster TIL-based measures (M^SCC^). M^SCC^ was statistically significantly prognostic of OS on the training sets (D^SCC^_1_ and D^SCC^_2_) with HR of 6.19 (95% CI: 2.08–18.4, *p* = 0.001, C-index: 0.664 (SE 0.059), *n* = 55). M^SCC^ was also prognostic of OS in D_3_ (*p* = 0.04, HR = 3.36; 95% CI, 1.08–10.5, C-index: 0.910 (SE 0.061), *n* = 21), D_4_ (*p* = 0.01, HR = 2.34; 95% CI, 1.21–4.5, C-index: 0.654 (SE 0.07), *n* = 86), and D_5_ (*p* = 0.07, HR = 1.38; 95% CI, 1.05–1.82, C-index: 0.590 (SE 0.039), *n* = 423) as illustrated in Fig. 2(G)–(I). M^SCC^ was evaluated using different lung SCC stages (I, II, III, and IV) across the cohorts D_3_–D_7_; results are shown in Supplementary Fig. 3(C–H). M^SCC^ yielded a statistically significant separation between low and high-risk groups (*n* = 279, *p* = 0.007, HR = 1.53, 95% CI: 1.12–2.09) in Stage I patients. Similarly, the trend is seen for Stage III (*n* = 73, *p* = 0.05, HR = 1.97, 95% CI: 0.99–3.92) and slight separation can be seen for Stage II (*n* = 167, *p* = 0.772, HR = 1.06, 95% CI: 0.72–1.55) but the differences did not rise to the level of statistical significance. M^SCC^ showed association with OS for Early-Stage disease (combined Stage I and II) (*n* = 446, *p* = 0.018, HR = 1.33, 95% CI: 1.05–1.7). For Late-Stage disease, the separation of the risk groups (*n* = 79, *p* = 0.064, HR = 1.84, 95% CI: 0.96–3.53) can be visually appreciated but the differences did not rise to the level of statistical significance. Interestingly, when M^AD^ and M^SCC^ were evaluated on SCC and AD patients respectively, neither model yielded a significant association with OS (See Supplementary Fig. 2(G–H)). A multivariable analysis was performed on the cohorts, after adjusting for the effects of clinicopathological variables (Table 2). Only the clinco-pathologic variables that were available across all cohorts were considered for this analysis. Gender was found to be significant for D_1_, D_2_, D_3_ and D_7_. Histological subtype was also found to be significant for D_1_, D_2_ and D_3_. Only partial clinical information for D8 was available. Supplementary Table 3 shows the multivariable survival analysis performed using the training models (M^AD^ and M^SCC^) for some clinical variables (age, gender, and stage). In D_7_, M^AD^ was able to identify high and low risk groups in patients treated with Docetaxel plus Cisplatin or Carboplatin (*p* = 0.002, HR = 4.1, 95% CI: 1.55–10.82) (Supplementary Fig. 2(I–L)). A similar trend was observed for patients who received Cisplatin plus Navelbine or Docetaxel or Pemetrexed (p = 0.041, HR = 2.04, 95% CI: 1.02–4.08). For patients in D_8_ treated with single monotherapy Docetaxel, M^AD^ did not show to be prognostic of OS with an HR of 0.91 (95% CI: 0.66–1.26) (See Supplementary Fig. 2(M)). Further, M^AD^ was significantly associated with OS for those patients who received radiotherapy but not radiochemotherapy from D_5_ and D_7_ (*n* = 208, *p* = 8.7e^-07^, HR = 2.94, 95% CI: 1.88–4.62) (See Supplementary Fig. 6 (A–B)).

**Table 2:** Multivariable analysis of OS with PhenoTIL (MAD + MSCC) and clinical variables for each cohort (D1, D2, D3, D4, D5, D6, D7).

	Characteristics	Hazard ratio	95% CI	*P*	*n*
D1	Gender				
	Male	0.48	0.26–0.91	**0.025**	
	Female				
	Histologycal Subtpes				
	Squamous Cell Carcinoma	0.42	0.21–0.84	**0.014**	71
	Adenocarcinoma				
	Clinical Stage				
	I				
	II	0.22	0.05–0.94	**0.042**	66
	III	3.35	0.94–11.92	0.062	
	IV	1.44	0.44–4.77	0.55	66
	TNM Staging				
	N0				
	N1	0.69	0.35–1.36	0.287	59
	T1				
	T2	0.82	0.44–1.53	0.535	59
	PhenoTIL				
	Low Risk	0.81	0.45–1.46	0.489	71
D2	Gender				
	Male	0.39	0.21–0.72	**0.003**	71
	Female				
	Histologycal Subtpes				
	Squamous Cell Carcinoma	0.53	0.29–0.99	**0.048**	
	Adenocarcinoma				
	Clinical Stage				
	I				
	II	0.93	0.42–2.00	0.849	70
	III	2.16	0.91–5.12	0.081	70
	IV	3.09	1.04–9.16	**0.042**	
	TNM Staging				
	T1				
	T2	0.95	0.47–1.93	0.894	54
	T3	0.7	0.15–3.14	0.637	64
	PhenoTIL				
	Low Risk	0.43	0.23–0.79	**0.006**	
D3	Gender				
	Male	0.45	0.26–0.79	**0.005**	
	Female				
	Histologycal Subtpes				
	Squamous Cell Carcinoma	0.31	0.17–0.59	**<0.001**	79
	Adenocarcinoma				
	Clinical Stage				
	I				
	II	0.48	0.21–1.10	0.082	72
	III	2.62	1.23–5.58	**0.012**	
	IV	2.21	0.66–7.44	0.201	72
	PhenoTIL				
	Low Risk	0.54	0.31–0.92	**0.023**	79
D4	Histologycal Subtpes				
	Squamous Cell Carcinoma	0.79	0.58–1.07	0.13	376
	Adenocarcinoma				
	Clinical Stage				
	I				
	II	1.07	0.76–1.50	0.714	351
	TNM Staging				
	T1				
	T2	0.83	0.12–5.92	0.849	376
	PhenoTIL				
	Low Risk	0.9	0.71–1.14	0.365	
D5	Gender				
	Male	1.28	1.03–1.60	**0.026**	
	Female				
	Histologycal Subtpes				
	Squamous Cell Carcinoma	1.15	0.93–1.42	0.209	850
	Adenocarcinoma				
	Clinical Stage				
	I				
	II	1.94	1.27–2.11	**<0.001**	838
	III	2.12	1.59–2.81	**<0.001**	838
	IV	2.72	1.68–4.39	**<0.001**	838
	TNM Staging				
	T1				
	T2	1.29	0.99–1.68	0.06	841
	T3	1.96	1.36–2.82	**<0.001**	841
	T4	3.28	1.98–5.43	**<0.001**	841
	Tx	3.62	0.89–14.79	0.073	
	N0				
	N1	1.53	1.20–1.96	**<0.001**	
	N2	1.96	1.42–2.69	**<0.001**	840
	N3	1.39	0.34–5.59	0.646	
	Nx	1.29	0.57–2.92	0.538	840
	M0				
	M1	2.12	1.31–3.43	**0.002**	834
	Mx	1.08	0.81–1.43	0.598	834
	PhenoTIL				
	Low Risk	1.06	0.78–1.44	0.688	849
D6	Gender				
	Male	0.96	0.29–3.21	0.946	21
	Female				
	PhenoTIL				
	Low Risk	5.83	1.53–22.26	**0.01**	
D7	Gender				
	Male	2	1.14–3.52	**0.0016**	93
	Female				
	Clinical stage				
	I				
	II	1.34	0.36–4.99	0.665	43
	III	2.76	0.90–8.52	0.077	
	IV	2.64	0.48–14.44	0.263	43
	PhenoTIL				
	Low Risk	0.53	0.29–0.97	**0.041**	93

Values in bold are statistically significant by two-tailed test (*P* < 0.05).

For D_7_, patients were divided into low (≤50%) and high (>50%) PD-L1. Also, patients were represented into positive (>1%) and negative (≤1%) PD-L1 expression groups. M^AD^ was also found to be associated with OS for the low PD-L1 arm, with HR of 2.41 (*n* = 69, *p* = 0.013, 95% CI: 1.17–4.94, C-index: 0.689 (SE 0.074)) (See Fig. 3(M)). Conversely, for the high PD-L1 arm, M^AD^ did not show an association with OS (See Supplementary Fig. 6(C–F)).

### PhenoTIL comparison with density and spatial aspects of TIL

For comparison purposes, the prognostic ability of two models based on density of TILs (denTIL^SCC^ and denTIL^AD^) was assessed on datasets D_3_, D_4_, D_5_, and D_7_. These models, however, were not able to distinguish between patients treated with chemotherapy at low- and high- risk of death (*p* > 0.05) (Supplementary Fig. 4). Validation of the PhenoTIL models on D_8_ was done blindly in which we had access to whole slide images but not clinical data. Our algorithms were applied to the images to predict risk scores and patient labels, and then these values were sent to the BMS team, who carried out the statistical analysis and provided us with the corresponding results. Therefore, it was not possible to validate the current density of TILs models (denTIL^SCC^ and denTIL^AD^)”.

“In addition, the prognostic ability of two models based on density of TILs (denTIL^SCC^ and denTIL^AD^) was assessed on dataset D_6_. These models, however, were not able to distinguish between patients treated with immunotherapy at low and high risk of death (*p* > 0.05) (Supplementary Fig. 5).

The prognostic ability of the two models based on spatial location of TILs (spaTIL^SCC^ and spaTIL^AD^) was assessed on datasets D_3_, D_4_, D_5_, and D_7_. These models, however, were not able to distinguish between patients treated with chemotherapy at low and high-risk of death (*p* > 0.05) (Supplementary Fig. 5).

### PhenoTIL identified immune cell niches from pretreatment H&E samples were associated with overall survival in lung adenocarcinoma and squamous cell carcinoma patients treated with immunotherapy

For D_6_, patients who received nivolumab and were identified by M^AD^ as having a “low risk of death” had significantly longer survival time (*p* = 0.004, HR = 5.83, 95% CI: 1.53–22.26) compared to patients identified as high risk, (see Fig. 3(J)). An expert pathologist manually assessed and categorized the quantity of TILs as low TIL (1–33%), moderate TIL (34–66%), and high TIL (67–99%) (for more details see Fig. 3(L)). A total of 70% of the patients labeled as “high risk” by M^AD^ were found to have a low TIL count (1–33% of TILs) while 63.6% of the patients labeled as “low risk” by M^AD^ had a high TIL count (34–99% of TILs). For D_8_, M^AD^ showed an association with OS but did not reach statistical significance within the trial arm of patients who received nivolumab with an HR of 1.27 (*n* = 204, *p* = 0.15, 95% CI: 0.93–1.7), see Fig. 3(K)).

### Identified cluster patterns of TILs in H&E images are different morphologically and molecularly between lungs adenocarcinoma and squamous cell carcinoma

**Fig. 4:**
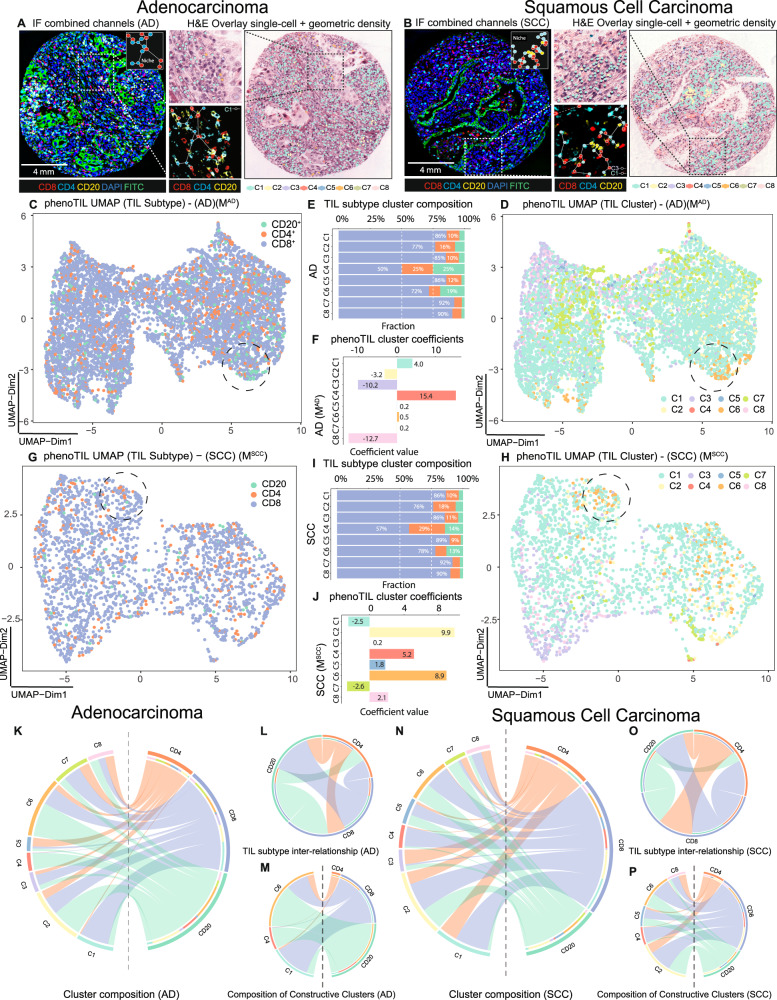
The molecular composition of immune subtypes and cluster association for lung AD and SCC sample. TIL subtype single-cell visual representation on an (**A**) lung AD and (**B**) SCC samples from cohort D_3_. The IF channels are displayed highlighting the spatial distribution of CD4^+^, CD8^+^, and CD20^+^ cells. Cluster labels are overlaid with corresponding H&E images. Smaller patches are shown to visualize the magnitude of complexity of cell detection and identification, depicting niche structures. **C** UMAP representation of TIL subtype composition for the lung AD. **D** UMAP of a lung AD sample cluster. **E** TIL subtype composition of clusters lung AD sample clusters has some concentrations of C1: CD4^+^ 10%, CD8^+^ 86%, CD20^+^ 4%; C4: CD4 + 25%, CD8^+^ 50%, CD20^+^ 25%; C6: CD4^+^ 9%, CD8^+^ 72%, CD20^+^ 19%. **F** The β coefficients (C1: 4.0, C2: −3.2, C3: −10.2, C4: 15.4, C5: 0.2, C6: 0.5, C7: 0.2, C8: −12.7) of the TIL cluster model (M^AD^). **G** UMAP representation of TIL subtype composition for the lung SCC. **H** UMAP of SCC sample clusters. **I** TIL subtype composition of SCC sample cluster. **J** The β coefficients (C1: −2.5, C2: 9.9, C3: 0.2, C4: 5.2, C5: 1.8, C6: 8.9, C7: −2.6, C8: 2.1) of the TIL cluster model (M^SCC^). **K** Chord diagram representing the cluster composition for lung AD. **L** Interconnection between the TIL subtypes and (**M**) between ‘constructive’ clusters. **N** Chord diagram representing the cluster composition for lung SCC. **O** Interconnection between the TIL subtypes and (**P**) ‘constructive’ clusters. CD4^+^ has a broader influence among clusters for SCC compared to AD. CD20^+^ plays a bigger role on the AD clusters.

A qualitative and quantitative evaluation of the TIL niches identified by PhenoTIL from H&E images was performed on images from D_3_. Differences in composition, spatial behavior, and intercellular communication of TIL subtypes (i.e., CD_4_, CD_8_, CD_20_) were analyzed for the different PhenoTIL clusters. Since TIL subtypes are not discernible on standard H&E samples, this analysis was carried out only on dataset D_3_ as it was the only one with subtype information available via QmIF. An illustration of the molecularly identified TIL subtypes (CD4^+^, CD8^+^ and CD20^+^) on the QmIF image is shown alongside the corresponding TIL cluster identified by M^AD^ on the co-registered H&E image (See Fig. 4(A)). A similar illustration was constructed for M^SCC^ (See Fig. 4(B)). A two-dimensional UMAP representation of the cell’s features was performed for the AD and SCC samples (See Fig. 4(D) and 4(H)), with corresponding QmIF labels (See Fig. 4(C) and 4(G)). The percentage of the QmIF subtype concentration among the M^AD^ and M^SCC^ clusters can be seen in Fig. 4(E) and 4(I) respectively.

By using the Elastic Net^31^ coefficients, major differences in the cluster involvement on survival for both the AD (M^AD^) and SCC (M^SCC^) can be observed in Fig. 3(F) and 3(J). When a cluster contribution toward a patients’ survival is found to be ‘constructive,’ it is interpreted as patients having longer survival times. For instance, for AD, clusters C1, C4, C5, C6, and C7 play constructive roles (Fig. 4(F)) while C2, C3, and C8 play obstructive roles. On the other hand, for SCC, clusters C2, C3, C4, C5, C6, and C8 play constructive roles while C1 and C8 play obstructive roles (See Methods or more details). Intercellular communication is examined using a chord diagram, which interrogates the inter- and intra-relationship between TIL subtypes and the clusters. The influence of the TIL subtypes among clusters for AD (See Fig. 4(K)–4(M)) and SCC (See Fig. 4(N)–4(P)) varies greatly.

**Fig. 5:**
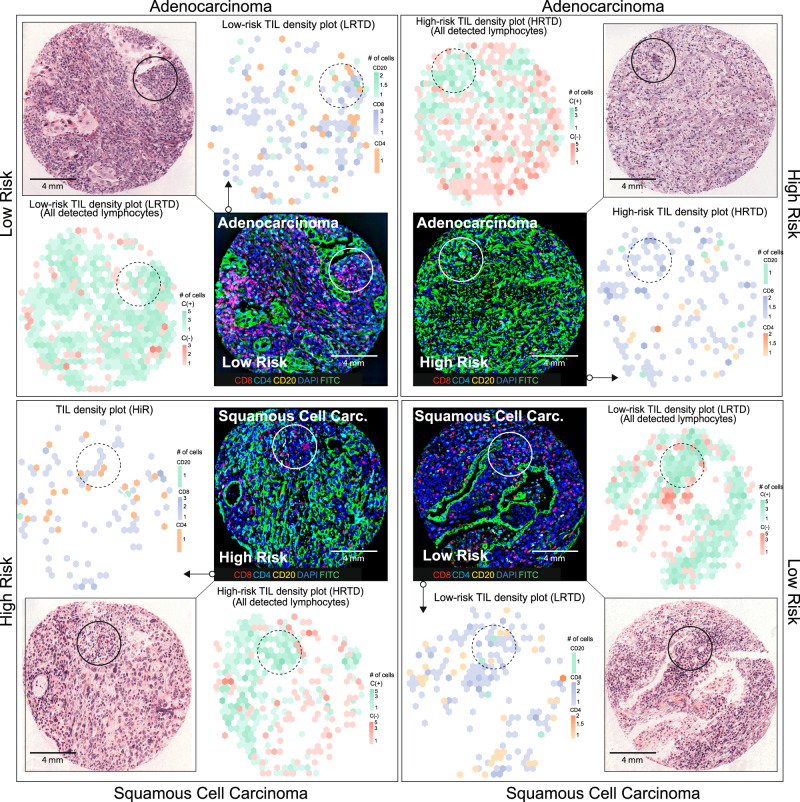
The density plots of TIL subtypes and cluster groups for lung AD and SCC are displayed alongside their corresponding H&E-stained and IF TMAs. The density plots based on the cluster’constructive’ C (+) and’obstructive’ C (−) are shown, depicting the concentration of each cluster group concentration. The TIL density plots also display concentration of TIL cells at the same position for each subtype, CD4^+^, CD8^+^ and CD20^+^. H&E samples are displayed at the outer layer of the figure and IF samples in the inside layer. The plots are shown for low- and high-risk groups assigned by the trained models (M^AD^ and ^MSCC^). For lung AD, CD20^+^ had a few cells spatially located to CD4^+^ pockets. high-risk TIL density plot (HRTD) sample displays CD8^+^ pockets to be sparse. CD20^+^ was found to be less frequent around CD8^+^ pockets. The ‘constructive’ clusters are seen to be forming highly concentrated pockets, compared to the ‘obstructive’ clusters, for both AD and SCC low-risk samples. Similarly, on the low-risk TIL density plot (LRTD), CD8^+^ can be seen forming concentrated pockets for AD. For SCC, the CD8^+^ pockets are sparser.

**Fig. 6:**
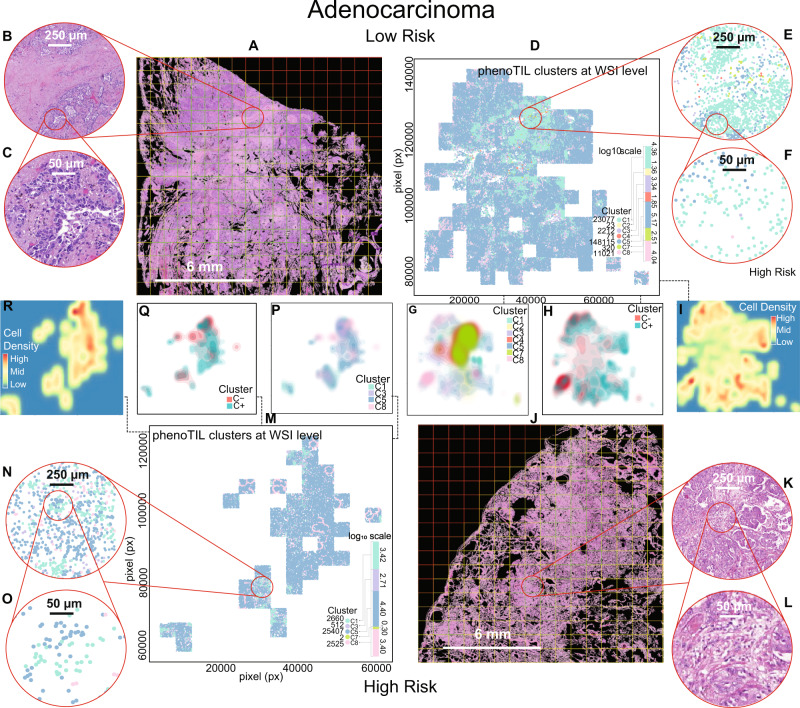
Representation of the TIL clusters at the Whole-Slide Image (WSI) level. Two adenocarcinoma samples, labeled as low and high-risk by the trained model (M^AD^) from cohort D_6_. **A** It displays the H&E WSI for a sample labeled as low-risk. The overlaid grid describes patches of 2000 × 2000 pixel size which colors indicates tissue content and usefulness (green = High amount of tissue content, Yellow = Medium amount of tissue, and red = Low to no tissue content). Tiles allocated into the green grid were used for this representation. It depicts a zoomed-in region of interest at (**B**) 250 µm and (**C**) 50 µm. For each tile, TIL features and clusters were generated for the (**D**) for the low-risk sample. The colored-bar size indicates the log10 scale of the quantity of cells for the sample. The quantity of cells for each cluster is shown next to each colored dot and label identifier. A zoom-in patch is displayed alongside, highlighting the conformation of different cluster labels at (**E**) 250 µm and (**F**) 50 µm. Density estimation plot is further shown. The density estimation is performed through a probability density function in 2-D by a kernel density estimate, generating a binning grid area. The higher the quantity of data points (cells) that fall within the binning grid area, the higher the color intensity. This is performed for (**G**) individual clusters, (**H**) the ‘constructive’ clusters labeled as C (+) and ‘obstructive’ as negative C (−) and (**I**) density estimation of all the detected lymphocyte cells, highlighting the densest areas in red tonality (‘High’). Similarly, for high-risk samples, the samples were utilized from (**J**) H&E images, zoomed-in (**K**, **L**), (**M**) TIL clusters at WSI level and (**N**, **O**) zoom-in. Density plots for (**P**) clusters, (**Q**) ‘constructive’ and ‘obstructive,’ and (**R**) lymphocyte density plot.

**Fig. 7:**
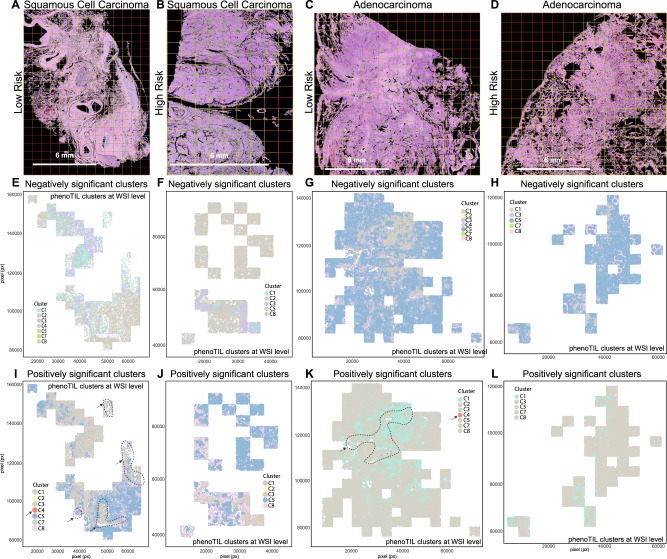
Cluster importance comparison among lung AD and SCC. The first two columns are squamous cell carcinoma cases (First and second columns are low and high-risk respectively). The last two columns are adenocarcinoma cases (Third and fourth columns are low and high-risk respectively). The first row represents the H&E WSI samples for lung AD (**A**, **B**) and SCC (**C**, **D**). Second row represents the plotting of cluster position that were assigned as ‘obstructive’ by the TIL model (M^SCC^) for (**E**, **F**) lung SCC and TIL model (M^AD^) for (**G**, **H**) lung AD. Third row represents the plotting of cluster position that were assigned as ‘constructive’ by the TIL model (M^SCC^) for (**I**, **J**) lung SCC and TIL model (M^AD^) for (**K**, **L**) lung AD model. For the low-risk samples (**I**, **K**), the influence of ‘constructive’ Cluster C4 around other clusters, is highlighted by the dotted-line region.

In Fig. 5, the density of cells for two low- and high-risk samples (two AD and two SCC) is illustrated. The density plots are illustrated next to their corresponding H&E and IF images. This is performed for both models (M^AD^ and M^SCC^). The clusters on M^AD^ were grouped based on their roles as either ‘constructive’ (C (+)) or ‘obstructive’ (C (−)). They were represented by a bivariate histogram (hexagonal bin plot), useful for visualizing the structure of multiple clusters of cells. The spatial arrangement of the TIL clusters is illustrated at the WSI level for a PhenoTIL identified low- and high-risk AD tumor (See Fig. 6). In addition, the spatial cell interaction of the ‘constructive’ and ‘obstructive’ clusters is shown across two SCC and AD tumors (See Fig. 7).

The distribution of the clusters across the risk groups revealed that for M^AD^, cluster C1 is more abundant for the low-risk cases. A similar trend is seen for TIL subtype CD8^+^. For M^SCC^, the trend is less clear, showing that the combination of clusters C2 and C6 participate in the low-risk cases (See Supplementary Fig. 7 (B–E)).

The morphometric measures used to construct TIL clusters by the models (M^AD^ and M^SCC^), are also shown across the risk groups for lung AD (Supplementary Fig. 8(A–D)) and SCC (Supplementary Fig. 8(E–H)). Further, the morphometric features identified as part of the model M^AD^, found to be associated with cluster C1, are mostly related to the texture of the TIL. For the low-risk groups, the feature shows a low variation in texture and color intensity, as opposed to the high-risk groups in which there is significantly high variation. Another feature implicated in M^AD^ is the distance between a TIL and non-TIL cell, which was significantly different, being shorter for tumors identified in the low-risk group and longer for the tumors in the high-risk group. This can be seen in the cluster of TILs associated with cluster C1 (See more details in the Supplementary Data 15).

### Biological pathway association of PhenoTIL clusters with Immune activation, regulation, and antigen presentation

**Fig. 8:**
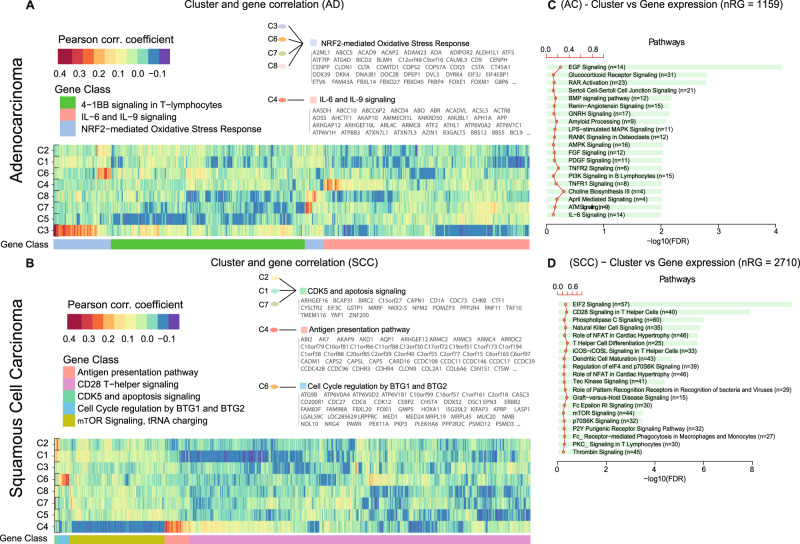
Gene expression and molecular pathways association with the immune clusters for lung AD and SCC. Heatmap representation of the significant gene expression for (**A**) lung AD. Each row represents an overly expressed gene. Each column indicates the cluster. The colors of the heatmap represent the Pearson correlation coefficient. The grouped genes are color-coded with a GO term representation. **B** The biological and molecular pathways are described in box plots. The most significant (FDR adjusted *p* value) terms are illustrated. The number of total associated gene signatures is shown as number of regulatory genes (nRG). Similarly, for lung SCC is shown (**C**) the gene expression heatmap and the (**D**) biological and molecular pathways is also included.

To understand the underlying biological processes associated with the PhenoTIL identified clusters, a gene enrichment analysis was performed. RNA sequencing of 20,531 genes was available for D_5_. For lung AD (*n* = 427), a functional profile of 1159 sets of genes were found to be significantly associated with the risk scores derived using the PhenoTIL-derived cluster measurements (M^AD^). From the identified set of genes for lung AD, the enrichment analysis identified 342 biological pathways (results with false discovery rate (FDR) *p* < 0.05), obtained from the Gene Ontology (GO) analysis platform^32,33^. Out of the 242 identified for AD, 24 pathways were found to be immune-related, from which 8 were shown to be associated directly with each PhenoTIL cluster for ADs. Pearson’s correlation coefficient was used to measure the strength and direction of the linear association between the immune-related pathways and the AD TIL clusters. The AD TIL clusters C3, C6, C7, and C8 were correlated with NRF2-mediated Oxidative Stress Response, C4 with IL-6 and IL-9 pathways. A heatmap illustrating the association between the most associated GO class and the individual TIL clusters determined by the TIL model (M^AD^) was constructed and illustrated in Fig. 8(A). The top 20 immune-related pathways, such as P13K signaling in B lymphocytes (B cell development) and TNFR2 signaling on regulatory T-cells (Increment on T-reg stability) are illustrated in Fig. 8(B).

For lung SCC (*n* = 423), 2710 genes were found to be significantly associated with the risk scores derived using M^SCC^. A total of 413 biological pathways (results with FDR *p* < 0.05) were found from which 30 pathways were immune-related. 10 were shown to be associated directly with each TIL cluster. TIL cluster C4 was found to negatively correlate with mTOR signaling pathway, which regulates T cell proliferation and orchestrates T cell quiescence^34^, and tRNA charging and was positively correlated with an antigen presentation pathway. Cluster C1 had a negative correlation with CD28 T-helper signaling. C7 positively correlates with CDK5 and apoptosis signaling. Cluster C6 had a positive correlation with cell cycle regulation by GTB1 and GTB2. Figure 8(C) illustrates the heatmap of the GO class associated with each TIL cluster from lung SCC determined by the model (M^SCC^). Figure 8(D) shows the top 20 immune-related pathways, such as natural killer cell signaling, T helper cell differentiation, ICos-ICosL signaling in T helper cells, and mTOR signaling identified for SCC tumors. The complete biological process, cellular component, molecular function, and gene pathways results based on the GO analysis are shown in Supplementary Fig. 9(A–B). In general terms, the most significant pathways found in lung AD and SCC were involved in, (a) the immune recognition, (b) the antigen presentation, (c) the antigen response and (d) the antigen regulation.

## Discussion

T cells are generated in the thymus and undergo further differentiation in the periphery to become specialized T cells such as CD8+ and CD4 + T cells. These cells effectively navigate to acute viral infection or tumor presence^35^. The tumor immune microenvironment (TIME) comprises abundant activated effector cytotoxic CD8+ and helper CD4+ tumor-infiltrating lymphocytes (TILs). The function of CD8 + T cells is to suppress tumors, by killing cancer cells with cytotoxic molecules. CD4 + T cells’ primary role is to mediate the anti-tumor immunity by stimulating CD8 + T cells^36^. Interestingly, a large quantity of cytotoxic CD8+ TILs found in TIME that have not been activated by tumor antigens behave as “bystanders”^9^. After a period of hyperresponsive state caused by chronic antigen stimulation, an activated CD8 + TIL undergoes ‘exhaustion’, characterized by high inhibitory expression and reduced cytotoxicity^10,37^. These diverse populations of immune cells form heterogeneous clusters with intricate communications within the TIME^38^. Due to the TIME complexity, estimating immunological frequency and TIL density may not be sufficient to describe cellular heterogeneity and their spatial distribution. However, capturing the complexity of the TIME could allow the development of prognostic and predictive biomarkers of response to current cancer treatments that target tumors (chemotherapy and radiation) and modulators of immune responses (immunotherapy).

Several studies have explored immune response mechanisms and their role in lung cancer treatment response^39^. For instance, Nejati et al. ^40^ demonstrated that high concentrations of intra-tumoral CD8^+^ and CD4^+^ TILs were associated with prolonged OS in patients treated with chemotherapy. A similar scenario was seen in lung SCC^19^ in which elevated levels of CD8+ or CD4+ TILs was associated with a longer disease-specific survival and disease-free survival, compared to patients with a lower concentration. In experiments using H&E slides, Wang et al. ^18^ explored the interplay between TIL and non-TIL cells in the tumor epithelium area, and identified spatial statistics related to TIL-cancer cell distances that were associated with OS and progression-free survival. Similarly, Azarianpour et al. ^15^ examined the spatial arrangement of TIL and cancer nuclei on H&E images, demonstrating an association between these spatial distance statistics with clinical benefit to chemotherapy, radiation therapy and nivolumab in gynecological cancers. Furthermore Ding et al. ^21^ explored both the density and spatial aspects of TILs and their association with outcome in patients treated with chemotherapy and nivolumab for lung AD and SCC. Also, finding the TIL density to be more crucial in patients with SCC and TIL spatial distribution for AD patients.

In this study, we introduced a new computationally-derived TIL phenotyping approach called ‘PhenoTIL’. PhenoTIL is a computational pathology approach that captures and employs the phenotypic and morphologic characteristics and spatial organization of TILs in the tumor microenvironment on H&E-stained tumor samples to identify distinct immune clusters, composition of which are associated with clinically relevant outcomes. In addition, PhenoTIL signature was able to stratify patients based on their risk of death using pre-treatment biopsy H&E-stained samples and a single cohort with post-neoadjuvant sections (D_7_), in the context of different therapy scenarios such as chemotherapy and targeted immunotherapy. Dedicated PhenoTIL models for characterizing the TIME in SCC and AD tumors were developed using 87 lung AD and 53 lung SCC patients respectively, subsequently evaluated for predicting OS on 1102 lung AD patients and 530 SCC patients. Unique PhenoTIL signatures that were associated with clinically relevant outcomes for squamous cell carcinomas and adenocarcinomas were identified.

In the context of adenocarcinomas, PhenoTIL was able to risk-stratify patients undergoing different therapies including chemotherapy and radiotherapy (Supplementary Fig. 6(A–B)). This trend was seen across dataset D_7_, in which chemotherapy was used in a neoadjuvant setting (i.e., before surgical resection) and scenarios with a combination of two chemo agents (Taxol plus platinum-based). The PhenoTIL model for adenocarcinomas (M^AD^) and squamous cell carcinoma (M^SCC^) were trained with most patients with early-stage (I and II) lung cancer, and the validation cohorts corresponded to patients from a plurality of staging groups (58.45%), indicating that the signatures were not specific to a particular disease stage, but the prognostic trend was observed most emphatically for early-stage disease. Most likely the immune reaction across the stages is highly variable, with late-stage immune landscape predominantly characterized by high levels of T-cell exhaustion^41^ and early-stage represented by a gradual transition from immune activation to immunosuppression, including the decrease of T-cell clonotypes, increase in regulatory T-cells infiltration and reduced infiltration of anti-tumor helper T cells^42^. For instance, despite PhenoTIL model M^AD^ not having a statistically significant stratification of risk (*p* = 0.12, HR = 1.27 (0.93–1.7)) in the phase 3 clinical trial cohort D_8_ (NCT01673867) of patients who received just Nivolumab, the Kaplan–Meir plots clearly revealed distinct risk groups (Fig. 3(K)). These patients were predominantly late-stage IV, so, surgery was not a viable option due to tumor dissemination. A similar behavior was seen for cohort D_6_, in which most of the patients were stage III and IV (76%) and were treated with Nivolumab (57%). More importantly, the PhenoTIL model M^AD^ was able to discriminate between groups of patients with a low and high risk of death in a low PD-L1 (<50%) setting, potentially allowing these patients who might otherwise be recommended for ICI-chemo combination therapy, to be candidates for ICI monotherapy; thereby potentially obviating the need for chemotherapy in a subset of LUAD patients with low PD-L1 expression.

At the tumor microenvironment level, we observed differences between the two NSCLC subtypes, with SCC exhibiting higher inter- and intra-tumoral heterogeneity than lung AD, which aligns with previous findings. For instance, some studies found that SCC tissue displayed enriched neutrophils at the cellular level compared to AD and has a more robust interaction with tumor cells^43^. Another study found that the spatial differences in PD-1 and CD8^+^ cells were significantly different between AD and SCC, regardless of the infiltration in the tumor proximity. In AD, a higher density of PD-1+ cells in the stroma and tumor islet cells was associated with shorter survival times while, in SCC, it was associated with better outcomes^44^. Using molecular and genomic analysis, we found that CD4^+^ and CD8^+^ T cell interactions played a key role in SCC patients with low risk of death while CD8^+^ and C20^+^ T cells were key components for lung AD patients with low risk of death. Regulation of the B-cells activation and proliferation, which play a critical role in recognizing antigens^45^, were correlated with ‘constructive’ clusters of TIL signature (M^AD^). Similarly, the ‘obstructive’ clusters were associated with NRF2-mediated oxidative stress response pathways, whose deficiency causes enhanced susceptibility to inflammatory diseases, deterioration, and oxidation;^46,47^, ultimately correlating with the exhausted state of T-cells. For the SCC PhenoTIL signature, the desirable clusters were found to be associated with mTOR signaling, which influences T-cell differentiation, stimulation, and proliferation, a major regulator of memory antigen-activated CD8^+^ T-cell differentiation, whose correlates play important roles in the TIME.

PhenoTIL is not the only approach that has characterized and used the spatial interplay of TILs, for instance studies carried out by Park et al. ^48^, Lopez de Rodas et al. ^49^, Wang et al. ^50^ and Ding et al. ^21^ have been exploring the use of computational imaging and artificial intelligence-based approaches for characterizing the density and spatial architecture of TILs in H&E images and their association with response to ICI and survival. Differences between our approach and these previously published studies include (1) the use of tiles or patches to obtain TIL-related features as opposed to our study which used the TIL immunophenotyping information on a cell-by-cell basis via the use of qmIF images, (2) risk stratification based on phenotypic traits of the TIL clusters, and (3) identifying molecular differences and distinct functions of the stratified TILs groups between lung tumor subtypes AD and SCC. In addition, the underlying hypothesis behind these studies^14,16–18,21,48–50^ remains the same, namely that all TILs are treated in a consistent and homogeneous manner.

The PhenoTIL approach identified clusters on the H&E images potentially represent individual aggregates of activated, exhausted, and ‘bystander’ immune cells, this hypothesis will need to be explicitly validated in future work. While we have not explicitly validated that PhenoTIL cluster mimics the functionality of each specific TIL subtype, we assume that the PhenoTIL signature appears to distinguish the unique formation of TIL clusters that resemble the functional aspects of activated TILs, which are prone to have a ‘constructive’ behavior toward tumor regulation and exhausted and bystander T cells, which have a more ‘obstructive’ behavior. The fundamental hypothesis of PhenoTIL, is that not all TILs uniformly contribute toward a prognostic signature for lung AD and SCC subtypes. Even though PhenoTIL does not explicitly identify individual molecular subtypes of TILs, it does appear to cluster the TILs into unique immune cell niches within the TIME, the architecture of which has clinical relevance with outcome for multiple therapy types.

Our study did have its limitations. One limitation was that the study design was retrospective in nature. In addition, although PhenoTIL was found to be prognostic of OS in both lung AD and SCC, the PhenoTIL signature M^AD^ was not associated with OS for patients who received either single-agent platinum or three-agent variations of multiple Taxol plus platinum. This behavior has been seen in patients benefiting from receiving combination compared to single-agent use^51^. This is probably due to the monotherapy scheme and outcome effects, relating to the biological difference reflected on the survival effect of TILs. Similar trends have been observed in the use of single chemo-agents which could inflict deleterious and inhibitory effects on the immune system and lymphocyte activation^52^. In addition, since both PhenoTIL signatures M^AD^ and M^SCC^ were trained solely on cohorts of patients treated with different chemotherapy agents, our signatures while associated with OS for individual therapies, were not explicitly validated as predictive for benefit of specific therapies. However, a strength of our approach was that the signature was associated with clinically relevant outcomes across different treatment types, reflecting the ability of the PhenoTIL signature to pick up biological hallmarks of good and poor tumor biology. Further, in this work, we studied the association between patient prognosis and nuclei clusters, built from contextual features (PhenoTIL), on the premise that cells in the tumor tend to act as groups rather than individually^53^. However, future work may benefit from analysis of the association between patient outcome and PhenoTIL features, independent of clusters.

In summary, we have developed and validated a computational biomarker, ‘PhenoTIL’, an immune-related biomarker associated with treatment-specific outcomes in NSCLC. It is also capable of capturing the immune phenotypes of TILs and their spatial interplay, allocating them into unique ‘immune clusters’ using digitized H&E-stained biopsy samples. PhenoTIL can also identify low-risk lung AD and SCC patients, which would allow clinicians to make adequate changes in their therapy management. Future work will involve prospective validation across independent cohorts and validation of the PhenoTIL signature as predictive of benefit of specific therapies.

## Methods

### Data and image processing

Figure 2 shows the overall workflow including image preparation, feature extraction, TIL single-cell cluster formation, molecular identification of TIL, and genomic pathway association for the PhenoTIL approach. As part of the inclusion criteria for all the datasets, images with low quality, blurry effects, and significant artefacts were excluded from the analysis. Image quality was checked using HistoQC^26^, an open-source quality control tool for digital pathology slides. HistoQC analyzes a set of WSIs and generates image masks indicating the regions that are not useful for analysis because of large blurry areas, obstructive dotting pen markings, or sub-coverslip bubbles. Images where the computationally valuable regions were either empty or smaller than 40% of the total real-estate of the slide were discarded. For this project, HistoQC was run using its default parameters^26^. This was performed for datasets D_5_, D_6_, D_7,_ and D_8_. For cohorts with TMA images, a previous study^54^ have mentioned that their use for evaluating TILs may be an issue due to TMAs not having substantial cell-related information and small core diameter may not reflect the entire tissue composition, nevertheless further studies as indicated by^55^ have shown that well-annotated TMAs datasets were optimal for drawing concordant results with TIL-related biomarkers and clinical outcome, indicating TMAs to be good option for this type of study. Regarding the TMA cohorts on our study, D_1_, D_2_, D_3,_ and D_4_, images that were not able to be processed for feature extraction due to lack of tissue (images containing 20% or less percentage of pixels in the tissue area) and sufficient nuclei detection (images containing 20 or less identified TIL and non-TILs) were removed. For D_6_, D_7,_ and D_8_ the additional inclusion criteria invoked included the availability of histologic subtype AD (See Fig. 1). Inclusion criteria for D_7_ were as follows: From the initial 211 patients, the considered patients were those who either underwent surgical resection after neoadjuvant therapy or had a primary resection at a locally advanced stage, which qualified them for neoadjuvant therapy. Three patients had more than one WSI scanned due to the following reasons: the first patient had a sample with neoadjuvant lung SCC with small AD as an incidental finding, the second was a patient with neoadjuvant adenosquamous carcinoma, and the third patient had a primary lung AD with three regions with very different growth patterns within the primary. Only one WSI containing the most tissue sample was selected for each patient. After invoking the inclusion and exclusion criteria for this study, 71 cases for D_1_ (49 excluded), 71 for D_2_ (35 excluded), 79 for D_3_ (57 excluded), 231 for D_4_ (49 excluded), 850 for D_5_ (239 excluded), 21 for D_6_ (49 excluded), 93 for D_7_ (118 excluded) and 358 for D_8_ (224 excluded) were included. Tiles of size 2048 by 2048 pixels (at 20× resolution; approx. 990 μm) were extracted from an automatic segmented tumor area^18^ of each WSI, from cohorts D_5_, D_6_, D_7,_ and D_8_. TMA spots from cohorts D_1_, D_2,_ and D_3_ were directly utilized. TMAs from D_4_ were down-sampled to 20× using nearest-neighbor interpolation. Supplementary Table 2 provides a summary of the image-related information for each cohort.

An inspection for assessing batch effects was performed using the subsequent full set of 288 PhenoTIL features across the different histologic image tiles and TMAs. The set of features were embedded into a two-dimensional feature space and plotted using UMAP^56^. The embedding shows (See Supplementary Fig. 7(A)) that there were no significant clusters of patients or isolation of samples by institutional site (cohort). This suggests that PhenoTIL features are resilient to batch effects and appear to be reproducible across the multiple sites/cohorts; in other words, no evidence of batch effects was identified.

### Nuclei segmentation and TIL identification

In D_5_, for each patient, 10 tiles of pixel size 2048 × 2048 (~990 μm) were extracted. Similarly, from D_6_, D_7,_ and D_8_, 10 tiles were obtained per WSI, outlined from an automatically segmented tumor area. For D_7_ multiple tiles were obtained from 4 WSIs for visualization purposes. The method developed by Wang et al. ^18^ was employed for the task of tumor segmentation. This method is a U-Net based convolutional network tuned using adversarial training, and it receives as input a WSI and outputs a tumor heatmap (See Supplementary Fig. 1(B)). Authors of the method report that, in a validation testing set containing 45 WSIs, the detector achieved a 90.6% patch-level accuracy^18^. The validation set is from the lung TCGA archives (TCGA-LUAD). The same cohort is used in the present study (D5). In addition, this model was used as is, i.e., it was not retrained or adjusted. The model was applied on each WSI and subsequently down-sampled to 20× through nearest-neighbor interpolation. The tumor detector was used to generate the heatmap for WSI to indicate the probability of tumor (90.6% patch-level accuracy) (See Supplementary Fig. 1(B)). D_4_ TMAs were down-sampled to 20× through nearest-neighbor interpolation. Two nuclei segmentation models were used to detect and segmentate the nuclei. First, the deep learning model introduced by Wang et al. ^18^ was used “as is” to delineate the boundary pixels of each nucleus. This model is also a U-Net network-tuned model, using adversarial training that receives a patch as input and then generates a mask indicating the location of the nuclei within the patch (and Supplementary Data 2). According to the authors, this model yielded an f-score of 0.88 in a dataset with 8000 nuclei annotated^18^. A second machine learning model based on morphological transformations and image processing using a watershed-based algorithm was used to further segmentate the nuclei^57^, resulted in a positive predictive value of 90% (Supplementary Data 3). Both models’ outputs were combined, and a single binary nuclei mask was obtained. Next, the model developed by Corredor et al. ^58^ is employed, as is, for classifying each segmented nucleus as either a TIL or non-TIL. It receives as input both an image patch and its respective nuclei segmentation mask (obtained using the method previously described), and it outputs the location of TILs and non-TILs within the image. Authors report that this model is a support vector machine trained visual features (texture, shape, and color), which yielded an f-score of 0.86 in validation phase^58^. Approximately 53,000 TILs were identified by automatically counting the total detected amount from lung AD patients (*n* = 87) in D^AD^_1_ and D^AD^_2_, and 40,000 TILs were identified from SCC (*n* = 55) in D^SCC^_1_ and D^SCC^_2_. Due to the color variation of images acquired from different scanners, color-normalization was applied to the H&E images using a technique a spectral matching named Macenko’s normalization^59^ to ensure coherent color representation across all datasets. An expert pathologist performed a manual TIL assessment for cohort D_6_ and assigned a label of 1–33%, 34–66%, and 67–99%, based on the percentage of infiltrating lymphocytes.

### PhenoTIL: TIL-based feature extraction and cluster computation

Characterization of the interaction of TILs within the tumor microenvironment was done by quantifying their spatial interaction with all other neighboring cells^30^. The algorithm comprises four steps: (1) The rectangular position (two-dimensional coordinate with positions, x, and y) of each TIL is obtained. (2) Iteratively, the algorithm locates each TIL and from its centroid coordinate, three circles are defined with incremental increasing radii of k = dL*10, dL*20, and dL*30 pixels (dL = 20 pixels, the average diameter of lymphocytes at 20× resolution. For a visual representation, see Supplementary Fig. 10(A)). (3) At each circle, a set of cell features were extracted from the neighboring cells including morphological characteristics (e.g., size, orientation, diameter, color intensity), density (e.g., number of surrounding lymphocytes), cell texture (Haralick et al. ^60^) And graph-based metrics (e.g., distances between lymphocytes and non-lymphocytes) were computed (The complete list can be seen in the Supplementary Table 4 and Supplementary Data 1 and 4). This set of 288 features (See *PhenoTIL: TIL-based digital risk score and statistical analysis*) characterize each TIL. 4) TILs were then clustered based on the features using a Gaussian Mixture Model^61^, from which a total of 8 unique TIL clusters were found (For more details of the clustering process, see the Supplementary Data 5). Finally, a patient is represented by eight vectors. These vectors represent the eight unique TIL clusters (C1, C2, C3, C4, C5, C6, C7, C8). The total TIL-related data is represented as a single matrix of size 1774 × 8 (1774 patients and eight TIL vector clusters).

### IF and H&E image registration

For D_3_, two challenges were found when processing the immunofluorescence (IF) images. One of the challenges was the overexposure found across the different IF markers intensities, affecting the comparison between the different images. To address this issue, the quantitative immunofluorescence score calculated by AQUA (Automated quantitative analysis) was used^12^ for intensity normalization. Another challenge was that the IF and H&E-stained images were generated from consecutive tissue sections. Their alignment is imperfect on account of the partial overlap of different TIL subtypes markers on a single-cell basis and the variation in the degrees of misalignment between the corresponding H&E TMA boundaries. To match both images and address the geometric misalignment of the images, a piece-wise linear transformation^62^ and spatial transformation via MATLAB function *“cp2tform”* was applied.

### Single-cell molecular pairing of TIL in H&E with IF images

One of the first steps to interrogate the TILs was to assign a unique molecular label to each identified TIL on H&E images. Once the images are co-registered, the identification of the TIL subtypes is performed. The process is based on the comparison on a pixel level, of the intensities of each cell. For each detected cell, the membrane is isolated. The intensity of the immunofluorescence stain of each marker (CD4 + , CD8+ and CD20 + ) is quantified from within the membrane (See the Supplementary Fig. 10(B–D)). These values are then normalized by the AQUA value^12^ (See Methods section, *IF and H&E Image Registration*). Finally, the marker with the highest value is taken as the true label for the cell. This process is repeated for each TIL.

### Gene enrichment and pathway analysis

The enrichment of genes and pathway analysis is performed for dataset D_5_. The gene collection was obtained from TCGA. The quantification of gene expression by RNA-seq is obtained for both lung AD (TCGA-LUAD) and SCC (TCGA-LUSC). The data is prepared into a matrix of gene expressions for each patient. A correlation matrix is computed between the gene expression matrix and the TIL clusters obtained from each model (M^AD^ and M^SCC^). Pearson’s correlation coefficient is calculated to identify the genes highly associated (values higher than 50% of the maximum correlation value). These genes are referred to as ‘activation genes’ or differentially expressed genes. These genes were used to find on the gene ontology (GO) knowledgebase^32^, pathways of biological processes through enrichment analysis. The pathways for each cluster were identified and visualized using a heatmap and bar plot displaying the canonical pathways significantly overrepresented. The most significant pathways are identified and listed according to their FDR corrected *p* value, shown as (−) log_10_(FDR). The analysis was performed using the R package *TCGAbiolink*s^33^, an integrative analysis with genomic data commons data.

### PhenoTIL: TIL-based digital risk score and statistical analysis

Overall survival was defined as the time interval between the date of diagnosis and the date of death and was censored at the date of the last follow-up for those patients known to be alive. An OS risk score (*OSRS*) for each *i*^*th*^ patient is defined as ${{OSRS}}_{i}={\sum }_{i=1}^{n}{N}_{{ij}}{\beta }_{j}$, where *β*_*j*_ is the regularizing coefficients returned by the Elastic Net^31^ method and *N*_*ij*_ is the number of clusters for the *i*^*th*^ patient. These steps were performed for developing the PhenoTIL signatures for lung AD using D^AD^_1_ and D^AD^_2_ and SCC using D^SCC^_1_ and D^SCC^_2_. The *β*_*j*_ coefficients are also indicators of cluster contribution toward patients’ survival, clusters with +*β*_*j*_ value is ‘constructive’ and those with −*β*_*j*_ values are ‘obstructive.’ In summary an *OSRS* was computed for each patient as a linear combination of the clusters and their respective coefficients (Supplementary Data 6). The median value of all risk scores of patients was computed and used as a cutoff, so a patient whose risk score value is higher than the median is considered “high risk”, and a patient with a risk score value lower than the median is considered “low risk”. Two different survival Cox proportional hazard regression models^63^ are obtained for lung AD (M^AD^) and SCC (M^SCC^) from (D^AD^_1_ and D^AD^_2_) and (D^SCC^_1_ and D^SCC^_2_), respectively, and are validated on D_3_, D_4_, D_5_, D_6_, D_7,_ and D_8_. M^SCC^ is validated only on D_3_, D_4_, and D_5_. To evaluate the efficacy of M^AD^ and M^SCC^ to predict OS, the Mantel-Haenszel log-rank test^64^ was used to assess the difference in OS. *P* values were two-sided, and values under 0.05 were considered to be statistically significant. The performance of the models M^AD^ and M^SCC^ were evaluated employing Harrell’s concordance-index (C-index)^65^. Kaplan–Meier survival analysis was utilized to determine the difference of OS across the different patient risk groups.

Three statistical analyses were performed between the TIL clusters across lung AD and SCC on D_3_. First, the statistical differences across the TIL clusters, e.g., how statistically different is cluster C1 on AD compared to cluster C1 on SCC (See Supplementary Table 5(A)). Second, the difference between AD from SCC regardless of the cluster labels, e.g., how statistically different are the TIL phenotypic features from AD vs SCC samples (See Supplementary Table 5(B)). Third, the statistical differences across the TIL subtypes (CD4^+^, CD8^+^, CD20^+^), e.g., how statistically different is the arrangement of CD8+ across all the clusters on AD compared to all the clusters on SCC (See Supplementary Table 5(C)).

### DenTIL: Developing prognostic models using features based on density of TILs

19 features relating to the density of TILs (denTIL)^21^ were extracted from each patch. These features include descriptors such as ratio between the number of TILs and the tissue area, ratio between the number of TILs and the total number of nuclei, and ratio between the total area covered by TILs to the total area of the tissue. The final feature vector for each patient was obtained by computing the mean, median, skewness and kurtosis for each feature across all its constituent tiles (i.e., four features per patient). Finally, two different survival Cox proportional hazard regression models were trained for lung AD (denTIL^AD^) and SCC (denTIL^SCC^) from (D^AD^_1_ and D^AD^_2_) and (D^SCC^1 and D^SCC^_2_), respectively, and are validated on D_3_ to D_7_.

### SpaTIL: Developing prognostic models using features based on TIL architecture

350 features were used to quantify the spatial arrangement of TILs and spatial interaction between TILs and non-TILs (spaTIL)^21^ as extracted from each patch. These features include the area of TIL clusters and the intermixing of the TIL and non-TIL clusters, among others. The final feature vector for each patient was obtained by computing the mean, median, skewness and kurtosis for each feature across all its constituent tiles (i.e., four features per patient). Finally, two different survival Cox proportional hazard regression models were trained for lung AD (spaTIL^AD^) and SCC (spaTIL^SCC^) from (D^AD^_1_ and D^AD^_2_) and (D^SCC^_1_ and D^SCC^_2_), respectively, and are validated on D_3_ to D_7_.

### Reporting summary

Further information on research design is available in the Nature Research Reporting Summary linked to this article.

## Supplementary information

Supplementary Material

REPORTING SUMMARY

## Data Availability

Since the cases from the involved institutions are protected through institutional compliance, the clinical repository of cases can only be shared per specific institutional review board (IRB) requirements. Upon reasonable request, a data sharing agreement can be initiated between the interested parties and the clinical institution following institution-specific guidelines. This is applicable for Cohort D1, D2, D3, D4, D6, D7, and D8. D1, D2, D3 were provided by the Department of Pathology at Yale University (D2 were collected at Sotiria General Hospital and Patras University General Hospital at Greece but were made available from Yale Pathology). Clinical information was provided. D4 was obtained from the Cleveland Clinic (CCF). Clinical information was provided. D5 was generated by TCGA Research Network (http://cancergenome.nih.gov/), and they have made them publicly available. The diagnostic slide (H&E slides) as well as the RNA sequencing data (https://portal.gdc.cancer.gov/projects/TCGA-LUAD and https://portal.gdc.cancer.gov/projects/TCGA-LUSC). Clinical information can be found at their portal. D6 was provided by the University of Pennsylvania Hospital (UPenn). Clinical information was provided. D7 was provided by the University of Bern in Switzerland (UBern). Clinical information was provided. D_8_ was provided by Bristol-Myers Squibb (BMS), from the clinical trial CA209-057 (ClinicalTrials.gov identifier: NCT01673867). No clinical information was provided.

## Code availability

All the codes and guidance for MATLAB and Python scripts can be found on GitHub: https://github.com/maberyick/PhenoTIL_V1.

Nuclei Segmentation https://github.com/maberyick/nucleiSegmentationHEDL.

Lymphocyte detection https://github.com/maberyick/LympDetect_HE_ML_V2.
