# Supplementary material for: Deep computational image analysis of immune cell niches reveals treatment-specific outcome associations in lung cancer
Source: NPJ Precis Oncol. 2023 Jun 1;7:52. doi: 10.1038/s41698-023-00403-x (PMC10235089; doi:10.1038/s41698-023-00403-x)
Supplement: Supplementary file 1 — Supplementary Material [file 41698_2023_403_MOESM1_ESM.pdf]

Supplementary Material

Supplementary Data 1

PhenoTIL parameters for models and implementation: The PhenoTIL model is constructed with two different languages that can be put together without much effort. First, MATLAB was used to build a set of feature extraction scripts, of which consists of 288 vectors, that are generated from identified lymphocyte cells from the H&E (Hematoxylin & Eosin) images. The features describe from shape, form, color and texture of the cells. The second language, Python, it was used to take the lymphocyte cells with their 288 feature vectors and generate a set of 8 unique clusters. The outcome of the model then is used to build the survival models.

Supplementary Data 2

Tumor segmentation: The training of the tumor segmentation was done using the U-Net TensorFlow version. With adversarial training with rates of 0.001 and 0.01 for regular and adversarial training, respectively. Other parameters were leave as default. Two class for the output (n=background, 1=tumor tissue) and input is the color image (RGB channels). 100 epochs for training, batch size = 1, optimizer = adam. The input image is the smaller size representation of the H&E-stained whole slide image (WSI) (aprox. Range of 512x512 to 1024x1024 pixel size). The output image mask is of the same size and dimension as the input image.

Supplementary Data 3

MATLAB Nuclei segmentation and lymphocyte identification: The nuclei segmentation offered uses two different models (Deep learning based and Machine learning with MATLAB). For the Machine Learning, it is based on multiple view across the region of interests, allowing cells of different size to be detected. It uses a watershed approach. It follows certain steps, 1) pre-processing with color unmixing and morphological operators, 2) watershed segmentation at multiple scales, 3) post-processing for removal of possible false cells and 4) merging of final results. The parameters specified in the GitHub code of file (run_phenoTIL_matlabr2020b_featureExtraction.m) with the line ‘nuclei = getWatershedMask(img,1,4,12)’, specifies as the img: the input RGB H&E image, the color normalization (1=yes), the lower level of scape (4) and upper level of scale (12).
The identification of the lymphocytes from the annotated binary masks can be performed using a set of morphological features, it takes into account the shape and color variations of the cells in order to identify the lymphocyte candidates. They ended up to be identified as either lymphocytes or non-lymphocytes. A binary mask can be generated from each label.

Supplementary Data 4

MATLAB feature extraction: The set of features of phenoTIL are then extracted from the binary mask and the original H&E image. The output is a .mat file with the 288 feature vector for each of the identified

lymphocytes. The piece of code from the GitHub file (run_phenoTIL_matlabe2020b_featureExtraction.m) with the line ‘getAllFeatures_V2(input_path,output_path); ` will extract the phenoTIL features, with the path of the H&E image location (input_path), the path of saving the mat file with the features (output_path). To run the complete script, follow the README guide for run_phenoTIL_matlabr2020b_featureExtraction. The mat files contains the centroid (position) of the cells, the local set of features (e.g. shape, size, color intensity), the contextual features (spatial-related information, such as distance between cells, quantity of cells around a specific radio), the graph interplay features (it uses the local features and contextual features to form a set of features as e.g. the mean area of a group of cells from a specific distance of central cell).

Supplementary Data 5

Python cluster generation: The cluster conformation is performed using the set of extracted features (from MATLAB, previous step). It uses the mat files as the input for each sample. As the extraction was performed using Python 2.7, the README file contains the steps to reproduce this step (Prerequisites for Python (Machine Learning) and Running unsupervized clustering (Python)). In general the Python script contains a pre-trained scaler to normalize the set of features and a Gaussian Mixture Model (GMM) with Dirichlet Process (DP) to clusterize the lymphocyte candidate cells using the extracted set of features. The output features are then used and the cluster model predicts from those, with the output being the label of a cluster prediction (cluster 1 to cluster 8) for each lymphocyte cell. Thus, no single cell can be in two or more clusters. For a sample, the file is save as a mat file (to be used later for building the survival model) with a single column being the cluster label and the rows the single lymphocyte cells.

Supplementary Data 6

Survival Model: Once the cells are put into an specific cluster with corresponding label (1 to 8), the cells are then represented to a quantitative matrix. From a matrix of column being the cluster label and rows the cells, to a matrix of columns being cluster 1 to cluster 8 (8 columns) and a single row with the total count of cells for each cluster label (e.g. row as quantity 0, 14, 58, 33, 33, 12, 134, 45 and columns as cluster label C1, C2, C3, C4, C5, C6, C7, C8). The matrix is then build from all the samples of the training set. This is the input for the linear model with penalized maximum likelihood build by the MATLAB function Glmnet, offering a regularization with elastic net penalty at the grid of values for regularization parameter lambda, affecting or altering the 8 clusters vectors. The input matrix (x) is the mentioned cluster matrix and the response vector (y) is the clinical outcome, overall survival (given in months). This is helpful to determine which set of clusters are more incline to be associated with the clinical outcome and those that are not.

Supplementary Data 7

Feature description for lung AD and SCC clusters: The PhenoTIL features are based on the cell-by-cell description via local features (such as their intensity, shape, texture) within a pre-specified neighborhood. Each lymphocyte cell is assigned a metric of those distinctive descriptors. This is performed in different sizes of communities. K1 to K3 changes according to a constant distance (K1, K2, K3; equals to 10, 20, 30 times an average lymphocyte size in pixels at 20x magnification (approx. 30 pixels) respectively). K3 allows for encompassing more lymphocyte cells within its neighborhood compared to K1. Features from the connective tissue binding the cells are extracted as well as features that capture the associated contextual information. Once the 288 features (V1 to V288) are extracted, clusters are formed in an unsupervised manner. Features relevancy is also assessed for each cluster by using a minimum redundancy feature selection (mRMR). The relevant characteristics are selected by their statistical dependency with the clinical variable, overall survival. For instance, features can be selected to be mutually distant from one another but still have a statistically significant correlation to the clinical variable. mRMR is an approximation to maximizing the dependency between the joint distribution of the selected characteristics and the clinical variable. The outcome is a sorted list of features, with the first in line as the most relevant and the last feature as the least relevant.

The Supplementary Figures S5 correspond to the feature analysis for AD (Figure S5(A-D)) and SCC (Figure S5(E-H)) respectively. The images were generated using a ridgeline plot, a chart useful for visualization of feature distributions and for comparing the low and high-risk groups. In summary, the phenotypic attributes that were mostly found for AD samples were related to the quantity and color intensities of the lymphocyte cells, and their interplay with the non-lymphocyte cells. Furthermore, for SCC samples, the attributes were related to the shape, diameter, and eccentricity of lymphocyte cells, involving the color intensities of the connective tissue surrounding the lymphocyte cells.

Supplementary Data 8

Relevant features from cluster C1 in lung AD

Feature ID V159 Value description: The median value of the entropy associated with the cell's image intensity (red R channel only) of the individual lymphocytes within the region delimited by K3. Effect description: The feature distribution corresponding to the high-risk group is characterized by multiple peaks and valleys, in turn reflecting the variability in the intensity distribution of the red channel corresponding to the lymphocyte cells on the H&E images. In part, this variation is also reflective of the significant variation in the appearance of the lymphocytes that constitute this higher-risk group. For the low-risk group, the opposite trend was observed with no deep valleys being observed in the corresponding feature distribution, in turn suggesting a greater consistency and homogeneity with respect to the appearance of the individual lymphocytes.

Feature ID V106 Value description: Mean of the cell areas of individual lymphocytes within the region delimited by K1. Effect description: The feature distribution corresponding to the low-risk group is characterized by two peaks with a soft valley in between, in turn reflecting the low variability of the lymphocyte cell size on the H&E images. For the high-risk group, the opposite trend was observed with a steep peak and valley located at the lower interval (<20%) in the corresponding feature distribution. This in turn appears to suggest a higher variability in the size of lymphocyte cells and a great accumulation of small-sized lymphocytes in H&E images.

Feature ID V141 Value description: Variance in the average pixel intensity for individual lymphocytes within the region delimited by K3. Effect description: The feature distribution corresponding to the high-risk group is characterized by a dominant peak and a deep valley, suggesting that the color intensities of the

lymphocyte cells on the H&E images are not consistent. On the other hand, the low-risk group is characterized by 2-3 peaks and two deep valleys in-between with most of the values concentrated on the lower interval (<40%), in turn suggesting that the color intensities of the lymphocyte cells are highly variable.

Feature ID V213 Value description: Ratio between the median of the minimum distance between the lymphocyte cells, and the median of the minimum distances between the lymphocyte and closest non-lymphocyte cells within the region delimited by K3. Effect description: Both feature distributions corresponding to the high- and low-risk groups show a single peak concentrated on the lower end of the spectrum (<25%), with the high-risk group distribution having a much higher and more dominant peak, in turn reflecting the greater quantity of small-groups of lymphocyte cells neighboring non-lymphocytes cells on the H&E images.

Supplementary Data 9
Relevant features from cluster C2 in lung AD

Feature ID V185 Value description: The standard deviation of the ratio between the median intensity of the red (R) and green (G) pixel channels of the individual lymphocyte cells within the region delimited by K1. Effect description: The feature distribution corresponding to the low-risk group is characterized by a single peak located at the lower end of the range (<20%) without valleys, suggesting lymphocyte cells with lower signal intensity in the red channel are less dispersed and more clustered on H&E images. Conversely, the high-risk group is characterized by two peaks-one smaller- with a valley in-between in the corresponding feature distribution, suggesting that lymphocytes that displayed a low red color intensity, tend to be more dispersed and less clustered on H&E images.

Feature ID V220 Value description: Total tissue area in pixels from the lymphocyte centroid to the region delimited by K1. Effect description: Both feature distributions corresponding to the high- and low-risk groups show a single peak concentrated in the upper range of the intensity distribution (>85%), with the high-risk group distribution with a dominant and taller peak, in turn suggesting that the tissue area surrounding the lymphocytes on the H&E images occupy a larger area compared to the low-risk group.

Feature ID V198 Value description: Ratio between the number of lymphocyte and non-lymphocyte cells within the region delimited by K3. Effect description: The feature distribution corresponding to the low-risk group is characterized by a single peak located at the lower range of the distribution (<20%), in turn reflecting an aggregation of small clusters of lymphocytes in the H&E images. For the high-risk group, a similar trend is observed with a flat peak located at the lower end of the spectrum (<20%) in the corresponding feature distribution, in turn suggesting a more disordered organization of lymphocyte clusters in the H&E images.

Feature ID V167 Value description: The mean of the ratio between the median intensity of the red (R) and blue (B) pixel channels of the individual lymphocyte cells within the region delimited by K2. Effect description: The feature distribution corresponding to the low-risk group is characterized by multiple peaks and valleys, with the main dominant peak located at the center of the distribution (~50%), in turn suggesting that the intensities of the cells (in the red channels) are more dispersed on H&E images. For the high-risk group, the opposite trend was observed with no deep valleys being observed in the corresponding feature distribution. This variation suggests a lower-than-average consistency and homogeneity with respect to the appearance of the individual lymphocytes with high eosin color intensity.

Supplementary Data 10

Relevant features from cluster C3 in lung AD

Feature ID V159 Value description: The median of entropy values of the cell's image intensity (red channel only) of the individual lymphocytes within the region delimited by K3. Effect description: The feature distribution corresponding to the low-risk group is characterized by multiple peaks and valleys with most concentrated on the upper interval (>50%), in turn reflecting the large number of details of the cell (high entropy) due to the entropy distribution of the red channel corresponding to the lymphocyte cells on the H&E images. In part, this variation is also reflective of the significant detail in the appearance of the lymphocyte cells that constitute this low-risk group. For the high-risk group, a similar feature distribution is observed but with fewer deep valleys and peaks, with the main peaks located within the lower half of the distribution (<50%), in turn suggesting more consistency and homogeneity with respect to the appearance of the individual lymphocytes.

Feature ID V257 Value description: Haralick textural feature: sum of the distinct gray levels in the quantified image representing the connective tissue, characterized as a gray-level co-occurrence matrix

(GLCM) and delimited by K2. Effect description: The feature distribution corresponding to both low- and high-risk groups is characterized by two peaks and no valleys, with the main peak located within the top half of the distribution (>85%) and the feature distribution corresponding to the low-risk patients with the taller peak. This difference in the appearance of the distributions suggests an over-expression of texture feature values of the cell, in turn indicating an elevated heterogeneity among the lymphocytes on the H&E images.

Feature ID V285 Value description: Haralick textural feature: the difference between the rows and columns entropy of the distinct gray levels in the quantified image representing the connective tissue, characterized as a gray-level co-occurrence matrix (GLCM) and delimited by K3. Effect description: The feature distribution corresponding to the low-risk group is characterized by a single peak located within the top half of the distribution (>85%). The feature distribution corresponding to the high-risk group is characterized by two unequal peaks. For the feature distribution of the low-risk group, there appears to be high variability in the texture feature distribution of the cell, in turn reflecting an elevated heterogeneity among the lymphocytes on the H&E images.

Feature ID V110 Value description: Median of the cell areas of individual lymphocytes within the region delimited by K2. Effect description: The feature distribution corresponding to both low- and high-risk groups is characterized by multiple peaks and valleys, with the main peak located within the lower half of the distribution (<40%) and the feature distribution corresponding to the low-risk being located within the bottom 30% of the range.

In part, the feature variation found in the low-risk group indicates that a great number of lymphocytes have a small cell size.

Supplementary Data 11

Relevant features from cluster C8 in lung AD

Feature ID V159 Value description: The median of the entropy value of the image intensity (red R channel only) of the individual lymphocytes within the region delimited by K3. Effect description: The feature distribution corresponding to the high-risk group is characterized by peaks and a valley, reflecting the variability in the intensity distribution on the H&E images. In part, this variation is also reflective of the variability in the appearance of the lymphocytes that constitute this higher-risk group. For the low-risk group, the opposite trend was seen with no deep valleys but a single peak is observed in the corresponding feature distribution, in turn suggesting a greater consistency and homogeneity with respect to the appearance of the individual lymphocytes.

Feature ID V216 Value description: Number of lymphocyte cells found within the area enclosed by the non-lymphocyte cells within the region delimited by K3. Effect description: Both feature distributions

corresponding to the high- and low-risk groups show a single peak concentrated in the middle of the distribution, with the high-risk group distribution having smaller peaks along the feature distribution, in turn reflecting the fluctuating accumulation of lymphocyte cells neighbouring non-lymphocyte cells on the H&E images.

Feature ID V102 Value description: Count the number of lymphocyte cells within the region delimited by K3. Effect description: Both feature distributions corresponding to the high- and low-risk groups show a single peak concentrated at different close intervals, with the high-risk group distribution having a smaller peak, in turn reflecting the varying accumulation of lymphocyte cells on the H&E images.

Feature ID V111 Value description: Median of cell areas of individual lymphocytes within the region delimited by K3. Effect description: The feature distribution corresponding to the high-risk group is characterized by a single flat peak located within the center of the distribution (~50%) without valleys, suggesting lymphocyte cells that are smaller compared to a pre-determined size appeared to be less dispersed on H&E images. For the low-risk group, the feature distribution is characterized by two peaks with a small valley in between, reflecting that the low-risk group is represented by lymphocyte cells that are smaller in size but more dispersed on H&E images.

Supplementary Data 12

Relevant features from cluster C1 in lung SCC

Feature ID V138 Value description: Standard deviation in the size of lymphocytes within the region delimited by K3. Effect description: The feature distribution corresponding to the high-risk group is characterized by multiple peaks and valleys, suggesting lymphocytes that tend to be smaller are more dispersed on H&E images. For the low-risk group, the feature distribution is characterized by two peaks with a small valley, in turn reflecting the low variability in cell diameter among lymphocyte cells on the H&E images.

Feature ID V268 Value description: Maximum pixel intensity of the area from the lymphocyte centroid to the region delimited by K3. Effect description: Both feature distributions corresponding to the high- and low-risk groups show a single peak concentrated within the upper interval (>85%), with the low-risk group distribution with an even more pronounced and higher peak, in turn suggesting that the connective tissue surrounding the lymphocytes on the H&E images have a higher pixel intensity compared to the connective tissue corresponding to the high-risk group.

Feature ID V134 Value description: Median value of the cell diameters of lymphocytes within the region delimited by K2. Effect description: The feature distribution corresponding to the high-risk group is characterized by two peaks and a valley, suggesting lymphocytes with small cell diameters are more

dispersed on H&E images. For the low-risk group the feature distribution is characterized by a single peak, in turn reflecting the low variability in cell diameter on the H&E images.

Feature ID V251 Value description: Ratio between the median value of all the pixel intensities corresponding to the red (R) and green (G) channels of the connective tissue comprising the lymphocyte cells and delimited by K2. Effect description: Both feature distributions corresponding to the high- and low-risk groups reveal a single peak localized within the lower interval (<40%), with the high-risk group distribution characterized by a higher peak, in turn suggesting that the connective tissue surrounding the lymphocytes on the H&E images have a higher color intensity. In part, this variation is also reflective of the significant variation in the appearance of the connectivity tissue surrounding the lymphocytes that constitute the high-risk group. In comparison with the low-risk group, the corresponding feature distribution is reflective of the fact that the connectivity tissue has a lower pixel intensity.

Supplementary Data 13

Relevant features from cluster C2 in lung SCC

Feature ID V120 Value description: Mean value of cell eccentricities of lymphocytes within the region delimited by K3. Effect description: The feature distribution corresponding to the high-risk group is characterized by multiple peaks and valleys, in turn reflecting the variability in the size of the cell eccentricity corresponding to the lymphocytes on the H&E images. In part, this variation is also reflective of the significant variation in the appearance of the lymphocytes that constitute this higher risk group. For the low-risk group the feature distribution is characterized by two peaks proximal to each other, in turn reflecting the low variability in terms of cell shape eccentricity on the H&E images.

Feature ID V133 Value description: Median value of cell diameters of lymphocytes within the region delimited by K1. Effect description: The feature distribution corresponding to the high-risk group is characterized by two peaks and a valley suggesting that lymphocytes are more dispersed on H&E images and the cells have a higher variability in their diameter. In part, this variation is also reflective of the significant difference in the shape and appearance of the lymphocytes that constitute this higher risk group. For the low-risk group the feature distribution is characterized by a single peak, in turn reflecting the low variability in cell diameter on the H&E images.

Feature ID V183 Value description: The median of the ratio of the median value of the red (R) and green (G) channel intensities of the individual lymphocyte cells within the region delimited by K3. Effect description: The feature distribution corresponding to the low-risk group is characterized by two peaks and a valley, mostly concentrated within the lower interval (<20%), in turn reflecting the variability in the intensity distribution of the red channel corresponding to the lymphocyte cells on the H&E images. This in

turn reflects the variation in the color appearance of the lymphocytes that constitute this lower risk group. For the high-risk group, the opposite trend was observed with two peaks and a small valley being observed in the corresponding feature distribution, in turn suggesting greater consistency and homogeneity with respect to the appearance of the individual lymphocytes.

Feature ID V274 Value description: Ratio between the median of the red (R) and green (G) channel intensities of the tissue containing the lymphocyte cells from the lymphocyte centroid to the region delimited by K3. Effect description: Feature distributions corresponding to the high- and low-risk groups reveal a single peak, with the feature distribution of the low-risk group concentrated within the lower interval (~20%), indicating that the connective tissue surrounding the lymphocytes on H&E images has a higher pixel intensity for the red channel. This is also reflective of the significant variation in the color appearance of the connective tissue surrounding the lymphocytes that constitute the lower risk group. For the high-risk group, the opposite trend was observed with a single peak being observed in the corresponding feature distribution, in turn suggesting a greater inconsistency and heterogeneity with respect to the appearance of the connective tissue surrounding the lymphocytes.

Supplementary Data 14

Relevant features from cluster C5 in lung SCC

Feature ID V123 Value description: Median value of the cell eccentricities of lymphocytes within the region delimited by K3. Effect description: The feature distribution corresponding to the high-risk group is characterized by two peaks and a valley, mostly concentrated within the lower interval (~10%), in turn reflecting the low variability in terms of cell eccentricity. This is also reflective of the significant variation in the appearance of the lymphocytes that constitute this higher risk group. For the low-risk group the feature distribution is characterized by two peaks near each other, reflecting the low variability in terms of cell eccentricity.

Feature ID V263 Value description: Haralick textural feature: the information measure of correlation from the image representing the connective tissue, characterized as a gray-level co-occurrence matrix (GLCM) and delimited by K2. Effect description: Both feature distributions corresponding to the high- and low-risk groups show a single peak concentrated within the middle of the interval (~50%), with the valley of the high-risk group distribution being situated at a much lower level (20%). This suggests that the texture of the tissue surrounding the lymphocytes on the H&E image of the high-risk group has a lower expression (and hence lower heterogeneity) compared to the low-risk group.

Feature ID V226 Value description: Entropy of the red channel (R) of the tissue comprising the lymphocyte cells from the lymphocyte centroid to the region delimited by K1. Effect description: The feature distribution corresponding to the low-risk group is characterized by a single peak found within the upper interval (>70%), in turn reflecting the high entropy (greater detail) of the connective tissue

comprising the lymphocytes. This variation is also reflective of the significant detail in the appearance of the connective tissue that constitutes this low-risk group. For the high-risk group, the feature distribution is characterized by two peaks and a valley in-between, with the main peaks located within the middle interval (~50%). This in turn suggests consistency and homogeneity with respect to the appearance of the connective tissue containing the lymphocyte cells on the H&E images.

Feature ID V186 Value description: The standard deviation of the ratio between the median value of the red (R) and green (G) channels of the individual lymphocyte cells within the region delimited by K1. Effect description: The feature distribution corresponding to the low-risk group is characterized by two peaks and a valley, mostly concentrated within the lower interval (<40%), in turn reflecting the variability in the intensity distribution of the red channel. This also reflects the significant variation in the color appearance of the lymphocytes that constitute this lower-risk group. For the high-risk group, the opposite trend was observed with multiple peaks and valleys, reflecting an inconsistency and heterogeneity with respect to the appearance of the individual lymphocytes.

Supplementary Data 15
Relevant features from cluster C8 in lung SCC

Feature ID V158 Value description: The median value of the entropy associated with the red R channel intensity for the individual lymphocytes within the region delimited by K2. Effect description: The feature distribution corresponding to the low-risk group is characterized by two peaks and a valley, concentrated within the middle interval (~50%), reflecting the high cellular entropy within the red channel. This is also reflected in the significant detail in the appearance of the lymphocyte cells that constitute this low-risk group. For the high-risk group, a similar feature distribution is observed but with more peaks and valleys, with the main peaks located within the lower interval (~30%). This in turn suggests a low consistency and homogeneity with respect to the appearance of the individual lymphocytes.

Feature ID V273 Value description: Ratio between the median of the red (R) and blue (B) channel of the tissue containing the lymphocyte cells from the lymphocyte centroid to the region delimited by K3. Effect description: Both feature distributions corresponding to the high- and low-risk groups show a single peak, with the low-risk group feature distribution concentrated within the lower interval (~40%). This in turn reflects the significant variation in the color appearance of the connective tissue surrounding the lymphocytes that constitute the lower risk group. For the high-risk group, the opposite trend was observed with a single higher peak being observed in the corresponding feature distribution. This in turn reflects a greater inconsistency and heterogeneity with respect to the appearance of the connective tissue surrounding the lymphocytes for the higher risk group.

Feature ID V226 Value description: Entropy of the red channel (R) of the tissue containing the lymphocyte cells from the lymphocyte centroid to the region delimited by K1. Effect description: The

feature distribution corresponding to the low-risk group is characterized by two peaks, with the main peak situated at the upper interval (>90%). This is reflective of the significant level of detail in the appearance of the connective tissue that constitutes this low-risk group. Similarly, for the high-risk group, the feature distribution is characterized by two peaks. These results suggest consistency and homogeneity with respect to the appearance of the connective tissue comprising the lymphocyte cells on the H&E images.

Feature ID V134 Value description: Median value of the cell diameters of lymphocytes within the region delimited by K2. Effect description: The feature distribution corresponding to the high-risk group is characterized by two peaks and a valley, suggesting lymphocytes with a small diameter tend to be more dispersed on H&E images. For the low-risk group the feature distribution is characterized by a single peak, in turn reflecting the low variability in diameter on the H&E images.

Supplementary Table 1. summary of the clinical and pathologic information for the datasets (D1, D2, D3, D4, D5, D6, D7 and D8)

	D1 (n=71)	D2 (n=71)	D3 (N=79)	D4 (n=231)	D5 (n=850)	D6 (n=21)	D7 (n=93)	D8 (n=358)	total (n=1774)
**gender**									
female	36 (50.7%)	25 (35.2%)	39 (49.4%)	0 (0%)	346 (40.7%)	15 (71.4%)	45 (48.4%)	0 (0%)	506 (28.5%)
male	35 (49.3%)	46 (64.8%)	40 (50.6%)	0 (0%)	504 (59.3%)	6 (28.6%)	48 (51.6%)	0 (0%)	679 (38.3%)
missing	0 (0%)	0 (0%)	0 (0%)	231 (100%)	0 (0%)	0 (0%)	0 (0%)	358 (100%)	589 (33.2%)
**status**									
alive	23 (32.4%)	23 (32.4%)	16 (20.3%)	50 (21.6%)	506 (59.5%)	7 (33.3%)	43 (46.2%)	0 (0%)	668 (37.7%)
dead	48 (67.6%)	48 (67.6%)	63 (79.7%)	181 (78.4%)	344 (40.5%)	14 (66.7%)	50 (53.8%)	0 (0%)	748 (42.2%)
missing	0 (0%)	0 (0%)	0 (0%)	0 (0%)	0 (0%)	0 (0%)	0 (0%)	358 (100%)	358 (20.2%)
**age (years)**									
mean (sd)	64.7 (9.21)	64.8 (9.87)	63.9 (9.60)	NA (NA)	66.4 (9.32)	67.9 (5.42)	62.9 (9.56)	NA (NA)	65.7 (9.44)
median [min, max]	65.0 [33.0, 88.0]	68.0 [39.0, 90.0]	67.0 [33.0, 90.0]	NA (NA)	67.0 [33.0, 90.0]	67.0 [33.0, 90.0]	63.0 [31.0, 84.0]	NA (NA)	67.0 [33.0, 90.0]
missing information	0 (0%)	0 (0%)	0 (0%)	231 (100%)	13 (1.5%)	0 (0%)	0 (0%)	358 (100%)	623 (35.1%)
**stage**									
I	44 (62.0%)	39 (54.9%)	42 (53.2%)	161 (69.7%)	432 (50.8%)	1 (4.8%)	10 (10.8%)	0 (0%)	729 (41.1%)
II	10 (14.1%)	14 (19.7%)	11 (13.9%)	70 (30.3%)	244 (28.7%)	3 (14.3%)	10 (10.8%)	0 (0%)	362 (20.4%)
III	8 (11.3%)	13 (18.3%)	16 (20.3%)	0 (0%)	132 (15.5%)	3 (14.3%)	64 (68.8%)	23 (6.4%)	259 (14.6%)

IV	4 (5.6%)	4 (5.6%)	3 (3.8%)	0 (0%)	30 (3.5%)	14 (66.7%)	9 (9.7%)	280 (78.2 %)	344 (19.4%)
missing information	5 (7.0%)	1 (1.4%)	7 (8.9%)	0 (0%)	12 (1.4%)	0 (0%)	0 (0%)	55 (15.4 %)	80 (4.5%)
**stage (grouped)**									
early-stage (I/II)	54 (76.1%)	53 (74.6%)	53 (67.1%)	231 (100%)	676 (19.0%)	4 (19.0%)	20 (21.5%)	0 (0%)	1091 (61.5%)
late-stage (III/IV)	12 (16.9%)	17 (23.9%)	19 (24.1%)	0 (0%)	162 (19.1%)	17 (81.0%)	73 (78.5%)	358 (100%)	603 (34.0%)
missing information	5 (7.0%)	1 (1.4%)	7 (8.9%)	0 (0%)	12 (1.4%)	0 (0%)	0 (0%)	0 (0%)	80 (4.5%)
**therapy**									
chemotherapy/radiotherapy related	71 (100%)	71 (100%)	79 (100%)	231 (100%)	850 (100%)	0 (0%)	93 (100%)	154 (43.0 %)	1549 (87.3%)
immunotherapy related	0 (0%)	0 (0%)	0 (0%)	0 (0%)	0 (0%)	21 (100%)	0 (0%)	204 (57.0 %)	225 (12.7%)
**histology**									
adenocarcinoma (ad)	44 (62.0%)	43 (60.6%)	58 (73.4%)	145 (62.8 %)	427 (50.2%)	21 (100%)	93 (100%)	358 (100%)	1189 (67.0%)
squamous cell carcinoma (scc)	27 (38.0%)	28 (39.4%)	21 (26.6%)	86 (37.2 %)	423 (49.8%)	0 (0%)	0 (0%)	0 (0%)	585 (33.0%)

Supplementary Table 2. Summary of the cohort provenance and imagery details.

	**D1**	**D2**	**D3**	**D4**	**D5**	**D6**	**D7**	**D8**
**study**								
	training cohort no. 1	training cohort no. 2	validation cohort no. 1	validation cohort no. 2	validation cohort no. 3	validation cohort no. 4	validation cohort no. 5	validation cohort no. 5
**interest**								
	TILs significance in nsclc (h&e)	TILs significance in nsclc (h&e)	TILs significance in nsclc (immunofluorescence)	added benefit of chemotherapy in nsclc (h&e)	gene ontology (go) enrichment analysis	immuno-oncology (io) benefits. expert manual TIL assessment	chemotherapy benefits. pd-l1 expression	nivolumab benefit over docetaxel benefit in nonsquamous nsclc
**institution**								
	yale university	sotiria general hospital. patras university general hospital (greece)	yale university	cleveland clinic foundation (ccf)	the cancer genome atlas (tcga). a joint project of the national cancer institute and national human genome research institute	university of pennsylvania hospital	university of bern in switzerland (ubern)	bristol-myers squibb (bms
**scanner**								
	aperio scanscope cs whole-slide imager	aperio scanscope cs whole-slide imager	ventana iscan ht scanner	roche-ventana iscan ht scanner	different scanner modalities	philips intellisite pathology solution	pannoramic p250 flash III	aperio at2
**image type**								
	tumor microarrays (tma)	tumor microarrays (tma)	tumor microarrays (tma)	whole slide image	whole slide image	whole slide image	whole slide image	whole slide image
**magnification**								
	20x	20x	20x	20x	40x	40x	20x	20x
**description**								

	restrospective collections (1988 - 2003). clinico-pathological information collected from clinical and pathology records. approved by patient consent form or waiver of consent. all tissue used after approval from yale human investigation committee protocol no. 9505008219	retrospective collections (1991 and 2001). clinicopathological information was collected from clinical and pathology records. approved by patient consent form or waiver of consent	retrospective collections (1988 - 2003). clinicopathological information was collected from clinical and pathology records. approved by patient consent form or waiver of consent. all tissue used after approval from yale human investigation committee protocol no. 9505008219	retrospective chart review of patients continuously admitted in ccf with nsclc between 2005-2015	resection samples (frozen). all patients provided written informed consent to conduct genomic studies in accordance with local institutional review boards. committee of experts in lung cancer pathology performed a review of all samples to confirm histological subtypes	patients with diagnostic tumor biopsies were treated with nivolumab. diagnostic/pretreatment biopsies	total of 93 patients with locally-advanced luad. a subset of 43 patients were treated with chemotherapy prior to resection with neoadjuvant intention and 50 patients were primary resected luad with pathologically confirmed infiltration of lymph nodes of at least the mediastinal level. patient which were neoadjuvantly treated received platinum-based chemotherapy combinations (cisplatin plus docetaxel, carboplatin plus paclitaxel, cisplatin plus pemetrexed, cisplatin plus gemcitabine, cisplatin plus vinorelbine, cisplatin plus etoposide and others)	patients eligible had stage IIIB or IB. patients were followed for survival while receiving treatment and every 3 months after the treatment's discontinuation; recurrent nonsquamous. nsclc after radiation or surgical resection or progression during or after one prior platinum-based doublet chemotherapy regimen. tumor tissue obtained before treatment was not used in the selection of patients. tumor response was assessed using response evaluation criteria in solid tumors, version 1.1. complete eligibility criteria are provided in the study protocol nct01673867 at the website clinicaltrials.gov

Supplementary Table 3. Multivariable survival analysis (overall survival) on the training cohorts for the TIL model MAD (DAD1+DAD2) and MSCC (DSCC1+DSCC2). The assessment was performed on the lung ADC and SCC using the clinical variables, age, gender and diagnostic stages as covariates.

multivariable analysis on ad training set

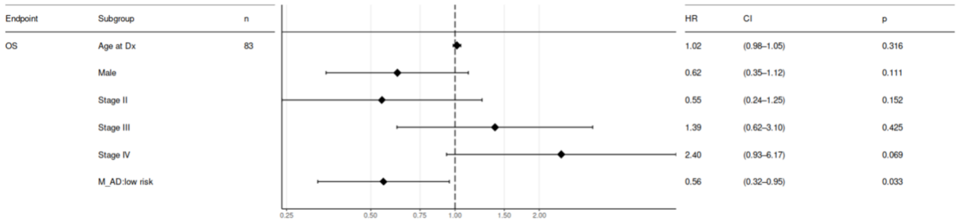

multivariable analysis on scc training set

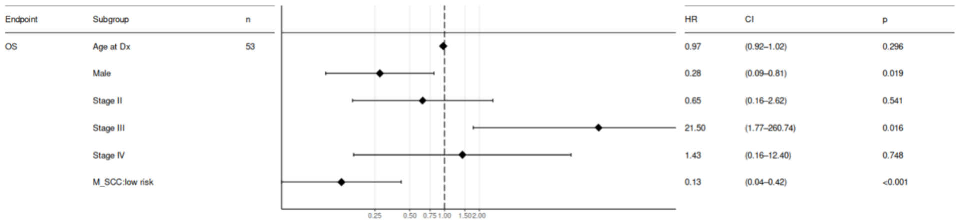

Supplementary Table 4. feature description of the feature involving local, contextual, and graph-based features of the TILs. the features were calculated on the basis of three different expanding radii (k1,k2,k3) allocating different quantities of cells on each one. some of the features extracted were from the cell (including nucleus and membrane). cells labeled as non-lymphocyte are composed of the majority of tumor cells as the regions involved are tumor areas. it accounts for 288 unique feature markers. the features are used to create the unsupervised clusters.

feature summary

local (n=99)	contextual (n=86)	graph-based interplay (n=103)
area	quantity of lymphocyte cells	Sum of the inverse distance (weighted) of lymphocytes
eccentricity	area size of lymphocyte cell	Ratio between the cell-area of lymphocytes and non-lymphocytes
equivalent diameter	eccentricity measurement of lymphocyte cell	Ratio between the cell-area of lymphocytes and the tissue sample holding the cells
orientation	diameter of lymphocyte cell	Differences between the pixel intensity of the lymphocyte cells
ratio between axes (x,y)	pixel intensity of the lymphocyte cell	Ratio between the area enclosed by lymphocytes and the area enclosed by the non-lymphocytes
entropy of the pixel intensities	entropy measurement of the lymphocyte cell	Intersected segment between the area enclosed by the lymphocytes and the area enclosed by the non-lymphocytes
maximum, medium and minimum pixel intensity	ratio between the red and blue color channel of the lymphocyte cell pixels	Average distance between the lymphocyte cell to non-lymphocyte cells
entropy of the red color channel	ratio between the red and green color channel of the lymphocyte cell pixels	Ratio between the average distance between lymphocyte cells and average distance between non-lymphocyte cells
the ratio between the red and blue color channel		Number of lymphocytes cells in non-lmyphocyte cells formed area
the ratio between the red and green color channel		Sum of the distances between lymphocytes cells to non-lymphocyte cells
**Haralick features extracted to the lymphocyte cell (nucleus and membrane) involving**		**Haralick features extracted from the tissue sample holding the cell involving**
energy angular second moment		energy angular second moment
contrast		contrast
correlation		correlation
variance		variance
inverse difference moment		inverse difference moment
sum average		sum average

sum variance		sum variance
sum entropy		sum entropy
entropy		entropy
difference variance		difference variance
difference entropy		difference entropy
information measures correlation I		information measures correlation I
information measures correlation II		information measures correlation II
maximal correlation coefficient		maximal correlation coefficient
**Zernike Polynomials (1 to 72)**		area of the tissue sample holding the cells
		entropy of the tissue sample holding the cells
		maximum, medium and minimum pixel intensity of the tissue sample holding the cells

complete list (288 features)

feature id	local feature key	feature id	contextual feature key	feature id	graph-based interplay feature key
v1	Area	v100	filtquantvarmatK1	v187	sumInvDistLympK1
v2	Eccentricity	v101	filtquantvarmatK2	v188	sumInvDistLympK2
v3	EquivDiameter	v102	filtquantvarmatK3	v189	sumInvDistLympK3
v4	Orientation	v103	filtareasvarmatK1	v190	RatioAreaLympNonLympK1
v5	ratioAxes	v104	filtareasvarmatK2	v191	RatioAreaLympNonLympK2
v6	entropyIntensity	v105	filtareasvarmatK3	v192	RatioAreaLympNonLympK3
v7	MaxIntensity	v106	filtareasmeanmatK1	v193	ratioAreaLympSampleK1
v8	medIntensity	v107	filtareasmeanmatK2	v194	ratioAreaLympSampleK2
v9	MinIntensity	v108	filtareasmeanmatK3	v195	ratioAreaLympSampleK3
v10	edgeMedIntensity	v109	filtareasmedianmatK1	v196	ratioNumLympNonLympK1

v11	entropyRed	v110	filtareasmedianmatK2	v197	ratioNumLympNonLympK2
v12	ratioMedRB	v111	filtareasmedianmatK3	v198	ratioNumLympNonLympK3
v13	ratioMedRG	v112	filtareasstdmatK1	v199	intensityDifferenceMedianK1
v14	harFeat_energyAngularSecondMoment	v113	filtareasstdmatK2	v200	intensityDifferenceMedianK2
v15	harFeat_Contrast	v114	filtareasstdmatK3	v201	intensityDifferenceMedianK3
v16	harFeat_correlation	v115	filteccentricityvarmatK1	v202	convexHullAreaRatioK1
v17	harFeat_Variance	v116	filteccentricityvarmatK2	v203	convexHullAreaRatioK2
v18	harFeat_InverseDifferenceMoment	v117	filteccentricityvarmatK3	v204	convexHullAreaRatioK3
v19	harFeat_SumAverage	v118	filteccentricitymeanmatK1	v205	convexHullIntersectedAreaK1
v20	harFeat_SumVariance	v119	filteccentricitymeanmatK2	v206	convexHullIntersectedAreaK2
v21	harFeat_SumEntropy	v120	filteccentricitymeanmatK3	v207	convexHullIntersectedAreaK3
v22	harFeat_Entropy	v121	filteccentricitymedianmatK1	v208	mediamDistLympNonLympNeigK1
v23	harFeat_DifferenceVariance	v122	filteccentricitymedianmatK2	v209	mediamDistLympNonLympNeigK2
v24	harFeat_DifferenceEntropy	v123	filteccentricitymedianmatK3	v210	mediamDistLympNonLympNeigK3
v25	harFeat_InformationMeasuresCorrelationI	v124	filteccentricitystdmatK1	v211	ratioMediamDistLympNonLympNeigK1
v26	harFeat_InformationMeasuresCorrelationII	v125	filteccentricitystdmatK2	v212	ratioMediamDistLympNonLympNeigK2
v27	harFeat_MaximalCorrelationCoefficient	v126	filteccentricitystdmatK3	v213	ratioMediamDistLympNonLympNeigK3
v28	zernFeat1	v127	filtdiametervarmatK1	v214	numLymInNonLympAreaK1
v29	zernFeat2	v128	filtdiametervarmatK2	v215	numLymInNonLympAreaK2
v30	zernFeat3	v129	filtdiametervarmatK3	v216	numLymInNonLympAreaK3

v31	zernFeat4	v130	filtdiametermeanmatK1	v217	sumDistLymptoNonLympK1
v32	zernFeat5	v131	filtdiametermeanmatK2	v218	sumDistLymptoNonLympK2
v33	zernFeat6	v132	filtdiametermeanmatK3	v219	sumDistLymptoNonLympK3
v34	zernFeat7	v133	filtdiametermedianmatK1	v220	sampleAreaK1
v35	zernFeat8	v134	filtdiametermedianmatK2	v221	sampleentropyIntensityK1
v36	zernFeat9	v135	filtdiametermedianmatK3	v222	sampleMaxIntensityK1
v37	zernFeat10	v136	filtdiameterstdmatK1	v223	samplemedIntensityK1
v38	zernFeat11	v137	filtdiameterstdmatK2	v224	sampleMinIntensityK1
v39	zernFeat12	v138	filtdiameterstdmatK3	v225	sampleedgeMedIntensityK1
v40	zernFeat13	v139	filtmeanIntensityvarmatK1	v226	sampleentropyRedK1
v41	zernFeat14	v140	filtmeanIntensityvarmatK2	v227	sampleratioMedRBK1
v42	zernFeat15	v141	filtmeanIntensityvarmatK3	v228	sampleratioMedRGK1
v43	zernFeat16	v142	filtmeanIntensitymeanmatK1	v229	sampleharFeat_energyAngularSecondMomentK1
v44	zernFeat17	v143	filtmeanIntensitymeanmatK2	v230	sampleharFeat_ContrastK1
v45	zernFeat18	v144	filtmeanIntensitymeanmatK3	v231	sampleharFeat_correlationK1
v46	zernFeat19	v145	filtmeanIntensitymedianmatK1	v232	sampleharFeat_VarianceK1
v47	zernFeat20	v146	filtmeanIntensitymedianmatK2	v233	sampleharFeat_InverseDifferenceMomentK1
v48	zernFeat21	v147	filtmeanIntensitymedianmatK3	v234	sampleharFeat_SumAverageK1
v49	zernFeat22	v148	filtmeanIntensitystdmatK1	v235	sampleharFeat_SumVarianceK1
v50	zernFeat23	v149	filtmeanIntensitystdmatK2	v236	sampleharFeat_SumEntropyK1

v51	zernFeat24	v150	filtmeanIntensitystdmatK3	v237	sampleharFeat_EntropyK1
v52	zernFeat25	v151	filtentropyRedvarmatK1	v238	sampleharFeat_DifferenceVarianceK1
v53	zernFeat26	v152	filtentropyRedvarmatK2	v239	sampleharFeat_DifferenceEntropyK1
v54	zernFeat27	v153	filtentropyRedvarmatK3	v240	sampleharFeat_InformationMeasuresCorrelationIK1
v55	zernFeat28	v154	filtentropyRedmeanmatK1	v241	sampleharFeat_InformationMeasuresCorrelationIIK1
v56	zernFeat29	v155	filtentropyRedmeanmatK2	v242	sampleharFeat_MaximalCorrelationCoefficientK1
v57	zernFeat30	v156	filtentropyRedmeanmatK3	v243	sampleAreaK2
v58	zernFeat31	v157	filtentropyRedmedianmatK1	v244	sampleentropyIntensityK2
v59	zernFeat32	v158	filtentropyRedmedianmatK2	v245	sampleMaxIntensityK2
v60	zernFeat33	v159	filtentropyRedmedianmatK3	v246	samplemedIntensityK2
v61	zernFeat34	v160	filtentropyRedstdmatK1	v247	sampleMinIntensityK2
v62	zernFeat35	v161	filtentropyRedstdmatK2	v248	sampleedgeMedIntensityK2
v63	zernFeat36	v162	filtentropyRedstdmatK3	v249	sampleentropyRedK2
v64	zernFeat37	v163	filtratioRedBbluevarmatK1	v250	sampleratioMedRBK2
v65	zernFeat38	v164	filtratioRedBbluevarmatK2	v251	sampleratioMedRGK2
v66	zernFeat39	v165	filtratioRedBbluevarmatK3	v252	sampleharFeat_energyAngularSecondMomentK2
v67	zernFeat40	v166	filtratioRedBbluemeanmatK1	v253	sampleharFeat_ContrastK2
v68	zernFeat41	v167	filtratioRedBbluemeanmatK2	v254	sampleharFeat_correlationK2
v69	zernFeat42	v168	filtratioRedBbluemeanmatK3	v255	sampleharFeat_VarianceK2

v70	zernFeat43	v169	filtratioRedBblu emedianmatK1	v256	sampleharFeat_Inve rseDifferenceMome ntK2
v71	zernFeat44	v170	filtratioRedBblu emedianmatK2	v257	sampleharFeat_Sum AverageK2
v72	zernFeat45	v171	filtratioRedBblu emedianmatK3	v258	sampleharFeat_Sum VarianceK2
v73	zernFeat46	v172	filtratioRedBblu estdmatK1	v259	sampleharFeat_Sum EntropyK2
v74	zernFeat47	v173	filtratioRedBblu estdmatK2	v260	sampleharFeat_Entr opyK2
v75	zernFeat48	v174	filtratioRedBblu estdmatK3	v261	sampleharFeat_Diff erenceVarianceK2
v76	zernFeat49	v175	filtratioRedGree nvarmatK1	v262	sampleharFeat_Diff erenceEntropyK2
v77	zernFeat50	v176	filtratioRedGree nvarmatK2	v263	sampleharFeat_Info rmationMeasuresCo rrelationIK2
v78	zernFeat51	v177	filtratioRedGree nvarmatK3	v264	sampleharFeat_Info rmationMeasuresCo rrelationIIK2
v79	zernFeat52	v178	filtratioRedGree nmeanmatK1	v265	sampleharFeat_Max imalCorrelationCoeff icientK2
v80	zernFeat53	v179	filtratioRedGree nmeanmatK2	v266	sampleAreaK3
v81	zernFeat54	v180	filtratioRedGree nmeanmatK3	v267	sampleentropyInten sityK3
v82	zernFeat55	v181	filtratioRedGree nmedianmatK1	v268	sampleMaxIntensity K3
v83	zernFeat56	v182	filtratioRedGree nmedianmatK2	v269	samplemedIntensity K3
v84	zernFeat57	v183	filtratioRedGree nmedianmatK3	v270	sampleMinIntensity K3
v85	zernFeat58	v184	filtratioRedGree nstdmat	v271	sampleedgeMedInte nsityK3
v86	zernFeat59	v185	filtratioRedGree nstdma2	v272	sampleentropyRedK 3
v87	zernFeat60	v186	filtratioRedGree nstdma3	v273	sampleratioMedRBK 3
v88	zernFeat61			v274	sampleratioMedRGK 3

v89	zernFeat62			v275	sampleharFeat_energyAngularSecondMomentK3
v90	zernFeat63			v276	sampleharFeat_ContrastK3
v91	zernFeat64			v277	sampleharFeat_correlationK3
v92	zernFeat65			v278	sampleharFeat_VarianceK3
v93	zernFeat66			v279	sampleharFeat_InverseDifferenceMomentK3
v94	zernFeat67			v280	sampleharFeat_SumAverageK3
v95	zernFeat68			v281	sampleharFeat_SumVarianceK3
v96	zernFeat69			v282	sampleharFeat_SumEntropyK3
v97	zernFeat70			v283	sampleharFeat_EntropyK3
v98	zernFeat71			v284	sampleharFeat_DifferenceVarianceK3
v99	zernFeat72			v285	sampleharFeat_DifferenceEntropyK3
				v286	sampleharFeat_InformationMeasuresCorrelationIK3
				v287	sampleharFeat_InformationMeasuresCorrelationIIK3
				v288	sampleharFeat_MaximalCorrelationCoefficientK3

Feature description

feature id	description
v1	area of the lymphocyte cell in pixels
v2	eccentricity of the lymphocyte cell in pixels
v3	equivalent diameter of the lymphocyte cell in pixels
v4	orientation of the lymphocyte cell in pixels
v5	ratio between the measurement of the y-axis and x-axis of the lymphocyte cell in pixels

v6	entropy of the grayscale image representation of the lymphocyte cell
v7	value of the pixel with the greatest intensity from the lymphocyte cell
v8	mean of the pixel values of intensity from the lymphocyte cell
v9	value of the pixel with the lowest intensity from the lymphocyte cell
v10	median intensity of the pixels conforming the perimeter area (membrane) around the lymphocyte cell
v11	entropy of the pixels with only the red channel (R) of the lymphocyte cell
v12	ratio between the median pixel value of the red channel (R) and the median pixel value of the blue channel (B) of the lymphocyte cell
v13	ratio between the median pixel value of the red channel (R) and the median pixel value of the green channel (G) of the lymphocyte cell
v14	Haralick value: angular second moment (energy) of the lymphocyte cell pixels characterized as a gray-level co-occurrence matrix
v15	Haralick value: gray-level "contrast" of the lymphocyte cell pixels characterized as a gray-level co-occurrence matrix
v16	Haralick value: gray-level "correlation" of the lymphocyte cell pixels characterized as a gray-level co-occurrence matrix
v17	Haralick value: gray-level "variance" of the lymphocyte cell pixels characterized as a gray-level co-occurrence matrix
v18	Haralick value: gray-level "inverse difference" of the lymphocyte cell pixels characterized as a gray-level co-occurrence matrix
v19	Haralick value: gray-level "sum average" of the lymphocyte cell pixels characterized as a gray-level co-occurrence matrix
v20	Haralick value: gray-level "sum variance" of the lymphocyte cell pixels characterized as a gray-level co-occurrence matrix
v21	Haralick value: gray-level "sum entropy" of the lymphocyte cell pixels characterized as a gray-level co-occurrence matrix
v22	Haralick value: gray-level "entropy" of the lymphocyte cell pixels characterized as a gray-level co-occurrence matrix
v23	Haralick value: gray-level "difference variance" of the lymphocyte cell pixels characterized as a gray-level co-occurrence matrix
v24	Haralick value: gray-level "difference entropy" of the lymphocyte cell pixels characterized as a gray-level co-occurrence matrix
v25	Haralick value: gray-level "information measure correlation I" of the lymphocyte cell pixels characterized as a gray-level co-occurrence matrix
v26	Haralick value: gray-level "information measure correlation II" of the lymphocyte cell pixels characterized as a gray-level co-occurrence matrix
v27	Haralick value: gray-level "maximal correlation coefficient" of the lymphocyte cell pixels characterized as a gray-level co-occurrence matrix
v28	Zernike value: amplitude of the moment of order 0; measured from the (arbitrary degrees, around its centroid, 0 times) binary mask of the lymphocyte cell
v29	Zernike value: phase (angle) in degree of order 0; measured from the (arbitrary degrees, around its centroid, 0 times) binary mask of the lymphocyte cell

v30	Zernike value: amplitude of the moment of order 1; measured from the rotated (anti-clockwise, arbitrary degrees, around its centroid, 1 times) binary mask of the lymphocyte cell
v31	Zernike value: phase (angle) in degree of order 1; measured from the rotated (anti-clockwise, arbitrary degrees, around its centroid, 1 times) binary mask of the lymphocyte cell
v32	Zernike value: amplitude of the moment of order 1; measured from the rotated (clockwise, arbitrary degrees, around its centroid, 1 times) binary mask of the lymphocyte cell
v33	Zernike value: phase (angle) in degree of order 1; measured from the rotated (clockwise, arbitrary degrees, around its centroid, 1 times) binary mask of the lymphocyte cell
v34	Zernike value: amplitude of the moment of order 2; measured from the rotated (anti-clockwise, arbitrary degrees, around its centroid, 2 times) binary mask of the lymphocyte cell
v35	Zernike value: phase (angle) in degree of order 2; measured from the rotated (anti-clockwise, arbitrary degrees, around its centroid, 2 times) binary mask of the lymphocyte cell
v36	Zernike value: amplitude of the moment of order 2; measured from the (arbitrary degrees, around its centroid, 0 times) binary mask of the lymphocyte cell
v37	Zernike value: phase (angle) in degree of order 2; measured from the (arbitrary degrees, around its centroid, 0 times) binary mask of the lymphocyte cell
v38	Zernike value: amplitude of the moment of order 2; measured from the rotated (clockwise, arbitrary degrees, around its centroid, 2 times) binary mask of the lymphocyte cell
v39	Zernike value: phase (angle) in degree of order 2; measured from the rotated (clockwise, arbitrary degrees, around its centroid, 2 times) binary mask of the lymphocyte cell
v40	Zernike value: amplitude of the moment of order 3; measured from the rotated (anti-clockwise, arbitrary degrees, around its centroid, 3 times) binary mask of the lymphocyte cell
v41	Zernike value: phase (angle) in degree of order 3; measured from the rotated (anti-clockwise, arbitrary degrees, around its centroid, 3 times) binary mask of the lymphocyte cell
v42	Zernike value: amplitude of the moment of order 3; measured from the rotated (anti-clockwise, arbitrary degrees, around its centroid, 1 times) binary mask of the lymphocyte cell
v43	Zernike value: phase (angle) in degree of order 3; measured from the rotated (anti-clockwise, arbitrary degrees, around its centroid, 1 times) binary mask of the lymphocyte cell
v44	Zernike value: amplitude of the moment of order 3; measured from the rotated (clockwise, arbitrary degrees, around its centroid, 1 times) binary mask of the lymphocyte cell
v45	Zernike value: phase (angle) in degree of order 3; measured from the rotated (clockwise, arbitrary degrees, around its centroid, 1 times) binary mask of the lymphocyte cell
v46	Zernike value: amplitude of the moment of order 3; measured from the rotated (clockwise, arbitrary degrees, around its centroid, 3 times) binary mask of the lymphocyte cell
v47	Zernike value: phase (angle) in degree of order 3; measured from the rotated (clockwise, arbitrary degrees, around its centroid, 3 times) binary mask of the lymphocyte cell
v48	Zernike value: amplitude of the moment of order 4; measured from the rotated (anti-clockwise, arbitrary degrees, around its centroid, 4 times) binary mask of the lymphocyte cell
v49	Zernike value: phase (angle) in degree of order 4; measured from the rotated (anti-clockwise, arbitrary degrees, around its centroid, 4 times) binary mask of the lymphocyte cell
v50	Zernike value: amplitude of the moment of order 4; measured from the rotated (anti-clockwise, arbitrary degrees, around its centroid, 2 times) binary mask of the lymphocyte cell

v51	Zernike value: phase (angle) in degree of order 4; measured from the rotated (anti-clockwise, arbitrary degrees, around its centroid, 2 times) binary mask of the lymphocyte cell
v52	Zernike value: amplitude of the moment of order 4; measured from the (arbitrary degrees, around its centroid, 0 times) binary mask of the lymphocyte cell
v53	Zernike value: phase (angle) in degree of order 4; measured from the (arbitrary degrees, around its centroid, 0 times) binary mask of the lymphocyte cell
v54	Zernike value: amplitude of the moment of order 4; measured from the rotated (clockwise, arbitrary degrees, around its centroid, 2 times) binary mask of the lymphocyte cell
v55	Zernike value: phase (angle) in degree of order 4; measured from the rotated (clockwise, arbitrary degrees, around its centroid, 2 times) binary mask of the lymphocyte cell
v56	Zernike value: amplitude of the moment of order 4; measured from the rotated (clockwise, arbitrary degrees, around its centroid, 4 times) binary mask of the lymphocyte cell
v57	Zernike value: phase (angle) in degree of order 4; measured from the rotated (clockwise, arbitrary degrees, around its centroid, 4 times) binary mask of the lymphocyte cell
v58	Zernike value: amplitude of the moment of order 5; measured from the rotated (anti-clockwise, arbitrary degrees, around its centroid, 5 times) binary mask of the lymphocyte cell
v59	Zernike value: phase (angle) in degree of order 5; measured from the rotated (anti-clockwise, arbitrary degrees, around its centroid, 5 times) binary mask of the lymphocyte cell
v60	Zernike value: amplitude of the moment of order 5; measured from the rotated (anti-clockwise, arbitrary degrees, around its centroid, 3 times) binary mask of the lymphocyte cell
v61	Zernike value: phase (angle) in degree of order 5; measured from the rotated (anti-clockwise, arbitrary degrees, around its centroid, 3 times) binary mask of the lymphocyte cell
v62	Zernike value: amplitude of the moment of order 5; measured from the rotated (anti-clockwise, arbitrary degrees, around its centroid, 1 times) binary mask of the lymphocyte cell
v63	Zernike value: phase (angle) in degree of order 5; measured from the rotated (anti-clockwise, arbitrary degrees, around its centroid, 1 times) binary mask of the lymphocyte cell
v64	Zernike value: amplitude of the moment of order 5; measured from the rotated (clockwise, arbitrary degrees, around its centroid, 1 times) binary mask of the lymphocyte cell
v65	Zernike value: phase (angle) in degree of order 5; measured from the rotated (clockwise, arbitrary degrees, around its centroid, 1 times) binary mask of the lymphocyte cell
v66	Zernike value: amplitude of the moment of order 5; measured from the rotated (clockwise, arbitrary degrees, around its centroid, 3 times) binary mask of the lymphocyte cell
v67	Zernike value: phase (angle) in degree of order 5; measured from the rotated (clockwise, arbitrary degrees, around its centroid, 3 times) binary mask of the lymphocyte cell
v68	Zernike value: amplitude of the moment of order 5; measured from the rotated (clockwise, arbitrary degrees, around its centroid, 5 times) binary mask of the lymphocyte cell
v69	Zernike value: phase (angle) in degree of order 5; measured from the rotated (clockwise, arbitrary degrees, around its centroid, 5 times) binary mask of the lymphocyte cell
v70	Zernike value: amplitude of the moment of order 6; measured from the rotated (anti-clockwise, arbitrary degrees, around its centroid, 6 times) binary mask of the lymphocyte cell
v71	Zernike value: phase (angle) in degree of order 6; measured from the rotated (anti-clockwise, arbitrary degrees, around its centroid, 6 times) binary mask of the lymphocyte cell

v72	Zernike value: amplitude of the moment of order 6; measured from the rotated (anti-clockwise, arbitrary degrees, around its centroid, 4 times) binary mask of the lymphocyte cell
v73	Zernike value: phase (angle) in degree of order 6; measured from the rotated (anti-clockwise, arbitrary degrees, around its centroid, 4 times) binary mask of the lymphocyte cell
v74	Zernike value: amplitude of the moment of order 6; measured from the rotated (anti-clockwise, arbitrary degrees, around its centroid, 2 times) binary mask of the lymphocyte cell
v75	Zernike value: phase (angle) in degree of order 6; measured from the rotated (anti-clockwise, arbitrary degrees, around its centroid, 2 times) binary mask of the lymphocyte cell
v76	Zernike value: amplitude of the moment of order 6; measured from the (arbitrary degrees, around its centroid, 0 times) binary mask of the lymphocyte cell
v77	Zernike value: phase (angle) in degree of order 6; measured from the (arbitrary degrees, around its centroid, 0 times) binary mask of the lymphocyte cell
v78	Zernike value: amplitude of the moment of order 6; measured from the rotated (clockwise, arbitrary degrees, around its centroid, 2 times) binary mask of the lymphocyte cell
v79	Zernike value: phase (angle) in degree of order 6; measured from the rotated (clockwise, arbitrary degrees, around its centroid, 2 times) binary mask of the lymphocyte cell
v80	Zernike value: amplitude of the moment of order 6; measured from the rotated (clockwise, arbitrary degrees, around its centroid, 4 times) binary mask of the lymphocyte cell
v81	Zernike value: phase (angle) in degree of order 6; measured from the rotated (clockwise, arbitrary degrees, around its centroid, 4 times) binary mask of the lymphocyte cell
v82	Zernike value: amplitude of the moment of order 6; measured from the rotated (clockwise, arbitrary degrees, around its centroid, 6 times) binary mask of the lymphocyte cell
v83	Zernike value: phase (angle) in degree of order 6; measured from the rotated (clockwise, arbitrary degrees, around its centroid, 6 times) binary mask of the lymphocyte cell
v84	Zernike value: amplitude of the moment of order 7; measured from the rotated (anti-clockwise, arbitrary degrees, around its centroid, 7 times) binary mask of the lymphocyte cell
v85	Zernike value: phase (angle) in degree of order 7; measured from the rotated (anti-clockwise, arbitrary degrees, around its centroid, 7 times) binary mask of the lymphocyte cell
v86	Zernike value: amplitude of the moment of order 7; measured from the rotated (anti-clockwise, arbitrary degrees, around its centroid, 5 times) binary mask of the lymphocyte cell
v87	Zernike value: phase (angle) in degree of order 7; measured from the rotated (anti-clockwise, arbitrary degrees, around its centroid, 5 times) binary mask of the lymphocyte cell
v88	Zernike value: amplitude of the moment of order 7; measured from the rotated (anti-clockwise, arbitrary degrees, around its centroid, 3 times) binary mask of the lymphocyte cell
v89	Zernike value: phase (angle) in degree of order 7; measured from the rotated (anti-clockwise, arbitrary degrees, around its centroid, 3 times) binary mask of the lymphocyte cell
v90	Zernike value: amplitude of the moment of order 7; measured from the rotated (anti-clockwise, arbitrary degrees, around its centroid, 1 times) binary mask of the lymphocyte cell
v91	Zernike value: phase (angle) in degree of order 7; measured from the rotated (anti-clockwise, arbitrary degrees, around its centroid, 1 times) binary mask of the lymphocyte cell
v92	Zernike value: amplitude of the moment of order 7; measured from the rotated (clockwise, arbitrary degrees, around its centroid, 3 times) binary mask of the lymphocyte cell

v93	Zernike value: phase (angle) in degree of order 7; measured from the rotated (clockwise, arbitrary degrees, around its centroid, 3 times) binary mask of the lymphocyte cell
v94	Zernike value: amplitude of the moment of order 7; measured from the rotated (clockwise, arbitrary degrees, around its centroid, 5 times) binary mask of the lymphocyte cell
v95	Zernike value: phase (angle) in degree of order 7; measured from the rotated (clockwise, arbitrary degrees, around its centroid, 5 times) binary mask of the lymphocyte cell
v96	Zernike value: amplitude of the moment of order 7; measured from the rotated (clockwise, arbitrary degrees, around its centroid, 7 times) binary mask of the lymphocyte cell
v97	Zernike value: phase (angle) in degree of order 7; measured from the rotated (clockwise, arbitrary degrees, around its centroid, 7 times) binary mask of the lymphocyte cell
v98	Zernike value: phase (angle) in degree of order 7; measured from the rotated (clockwise, arbitrary degrees, around its centroid, 5 times) binary mask of the lymphocyte cell
v99	Zernike value: phase (angle) in degree of order 7; measured from the rotated (clockwise, arbitrary degrees, around its centroid, 7 times) binary mask of the lymphocyte cell
v100	count the number of lymphocyte cells within the region delimited by K1
v101	count the number of lymphocyte cells within the region delimited by K2
v102	count the number of lymphocyte cells within the region delimited by K3
v103	variance in the cell areas of individual lymphocytes within the region delimited by K1
v104	variance in the cell areas of individual lymphocytes within the region delimited by K2
v105	variance in the cell areas of individual lymphocytes within the region delimited by K3
v106	mean in the cell areas of individual lymphocytes within the region delimited by K1
v107	mean in the cell areas of individual lymphocytes within the region delimited by K2
v108	mean in the cell areas of individual lymphocytes within the region delimited by K3
v109	median in the cell areas of individual lymphocytes within the region delimited by K1
v110	median in the cell areas of individual lymphocytes within the region delimited by K2
v111	median in the cell areas of individual lymphocytes within the region delimited by K3
v112	standard deviation in the cell areas of individual lymphocytes within the region delimited by K1
v113	standard deviation in the cell areas of individual lymphocytes within the region delimited by K2
v114	standard deviation in the cell areas of individual lymphocytes within the region delimited by K3
v115	variance in the cell eccentricities of lymphocytes within the region delimited by K1
v116	variance in the cell eccentricities of lymphocytes within the region delimited by K2
v117	variance in the cell eccentricities of lymphocytes within the region delimited by K3
v118	mean in the cell eccentricities of lymphocytes within the region delimited by K1
v119	mean in the cell eccentricities of lymphocytes within the region delimited by K2
v120	mean in the cell eccentricities of lymphocytes within the region delimited by K3
v121	median in the cell eccentricities of lymphocytes within the region delimited by K1

v122	median in the cell eccentricities of lymphocytes within the region delimited by K2
v123	median in the cell eccentricities of lymphocytes within the region delimited by K3
v124	standard deviation in the cell eccentricities of lymphocytes within the region delimited by K1
v125	standard deviation in the cell eccentricities of lymphocytes within the region delimited by K2
v126	standard deviation in the cell eccentricities of lymphocytes within the region delimited by K3
v127	variance in the cell diameters of lymphocytes within the region delimited by K1
v128	variance in the cell diameters of lymphocytes within the region delimited by K2
v129	variance in the cell diameters of lymphocytes within the region delimited by K3
v130	mean in the cell diameters of lymphocytes within the region delimited by K1
v131	mean in the cell diameters of lymphocytes within the region delimited by K2
v132	mean in the cell diameters of lymphocytes within the region delimited by K3
v133	median in the cell diameters of lymphocytes within the region delimited by K1
v134	median in the cell diameters of lymphocytes within the region delimited by K2
v135	median in the cell diameters of lymphocytes within the region delimited by K3
v136	standard deviation in the cell diameters of lymphocytes within the region delimited by K1
v137	standard deviation in the cell diameters of lymphocytes within the region delimited by K2
v138	standard deviation in the cell diameters of lymphocytes within the region delimited by K3
v139	variance in the average pixel intensity for individual lymphocytes within the region delimited by K1
v140	variance in the average pixel intensity for individual lymphocytes within the region delimited by K2
v141	variance in the average pixel intensity for individual lymphocytes within the region delimited by K3
v142	mean in the average pixel intensity for individual lymphocytes within the region delimited by K1
v143	mean in the average pixel intensity for individual lymphocytes within the region delimited by K2
v144	mean in the average pixel intensity for individual lymphocytes within the region delimited by K3
v145	median in the average pixel intensity for individual lymphocytes within the region delimited by K1
v146	median in the average pixel intensity for individual lymphocytes within the region delimited by K2
v147	median in the average pixel intensity for individual lymphocytes within the region delimited by K3
v148	standard deviation in the average pixel intensity for individual lymphocytes within the region delimited by K1
v149	standard deviation in the average pixel intensity for individual lymphocytes within the region delimited by K2
v150	standard deviation in the average pixel intensity for individual lymphocytes within the region delimited by K3
v151	the variance of entropies of the cell's images (red R channel only) for the individual lymphocytes within the region delimited by K1
v152	the variance of entropies of the cell's images (red R channel only) of the individual lymphocytes within the region delimited by K2

v153	the variance of entropies of the cell's images (red R channel only) of the individual lymphocytes within the region delimited by K3
v154	the mean of entropies of the cell's images (red R channel only) of the individual lymphocytes within the region delimited by K1
v155	the mean of entropies of the cell's images (red R channel only) of the individual lymphocytes within the region delimited by K2
v156	the mean of entropies of the cell's images (red R channel only) of the individual lymphocytes within the region delimited by K3
v157	the median of entropies of the cell's images (red R channel only) of the individual lymphocytes within the region delimited by K1
v158	the median of entropies of the cell's images (red R channel only) of the individual lymphocytes within the region delimited by K2
v159	the median of entropies of the cell's images (red R channel only) of the individual lymphocytes within the region delimited by K3
v160	the standard deviation of entropies of the cell's images (red R channel only) of the individual lymphocytes within the region delimited by K1
v161	the standard deviation of entropies of the cell's images (red R channel only) of the individual lymphocytes within the region delimited by K2
v162	the standard deviation of entropies of the cell's images (red R channel only) of the individual lymphocytes within the region delimited by K3
v163	the variance of the ratios (between the medians of the red (R) and blue (B) pixel channels) of the individual lymphocyte cells within the region delimited by K1
v164	the variance of the ratios (between the medians of the red (R) and blue (B) pixel channels) of the individual lymphocyte cells within the region delimited by K2
v165	the variance of the ratios (between the medians of the red (R) and blue (B) pixel channels) of the individual lymphocyte cells within the region delimited by K3
v166	the mean of the ratios (between the medians of the red (R) and blue (B) pixel channels) of the individual lymphocyte cells within the region delimited by K1
v167	the mean of the ratios (between the medians of the red (R) and blue (B) pixel channels) of the individual lymphocyte cells within the region delimited by K2
v168	the mean of the ratios (between the medians of the red (R) and blue (B) pixel channels) of the individual lymphocyte cells within the region delimited by K3
v169	the median of the ratios (between the medians of the red (R) and blue (B) pixel channels) of the individual lymphocyte cells within the region delimited by K1
v170	the median of the ratios (between the medians of the red (R) and blue (B) pixel channels) of the individual lymphocyte cells within the region delimited by K2
v171	the median of the ratios (between the medians of the red (R) and blue (B) pixel channels) of the individual lymphocyte cells within the region delimited by K3
v172	the standard deviation of the ratios (between the medians of the red (R) and blue (B) pixel channels) of the individual lymphocyte cells within the region delimited by K1
v173	the standard deviation of the ratios (between the medians of the red (R) and blue (B) pixel channels) of the individual lymphocyte cells within the region delimited by K2

v174	the standard deviation of the ratios (between the medians of the red (R) and blue (B) pixel channels) of the individual lymphocyte cells within the region delimited by K3
v175	the variance of the ratios (between the medians of the red (R) and green (G) pixel channels) of the individual lymphocyte cells within the region delimited by K1
v176	the variance of the ratios (between the medians of the red (R) and green (G) pixel channels) of the individual lymphocyte cells within the region delimited by K2
v177	the variance of the ratios (between the medians of the red (R) and green (G) pixel channels) of the individual lymphocyte cells within the region delimited by K3
v178	the mean of the ratios (between the medians of the red (R) and green (G) pixel channels) of the individual lymphocyte cells within the region delimited by K1
v179	the mean of the ratios (between the medians of the red (R) and green (G) pixel channels) of the individual lymphocyte cells within the region delimited by K2
v180	the mean of the ratios (between the medians of the red (R) and green (G) pixel channels) of the individual lymphocyte cells within the region delimited by K3
v181	the median of the ratios (between the medians of the red (R) and green (G) pixel channels) of the individual lymphocyte cells within the region delimited by K1
v182	the median of the ratios (between the medians of the red (R) and green (G) pixel channels) of the individual lymphocyte cells within the region delimited by K2
v183	the median of the ratios (between the medians of the red (R) and green (G) pixel channels) of the individual lymphocyte cells within the region delimited by K3
v184	the standard deviation of the ratios (between the medians of the red (R) and green (G) pixel channels) of the individual lymphocyte cells within the region delimited by K1
v185	the standard deviation of the ratios (between the medians of the red (R) and green (G) pixel channels) of the individual lymphocyte cells within the region delimited by K1
v186	the standard deviation of the ratios (between the medians of the red (R) and green (G) pixel channels) of the individual lymphocyte cells within the region delimited by K1
v187	sum of the inverse of the distances between the lymphocyte within the region delimited by K1
v188	sum of the inverse of the distances between the lymphocyte within the region delimited by K2
v189	sum of the inverse of the distances between the lymphocyte within the region delimited by K3
v190	ratio between the areas of lymphocyte and non-lymphocyte cells within the region delimited by K1
v191	ratio between the areas of lymphocyte and non-lymphocyte cells within the region delimited by K2
v192	ratio between the areas of lymphocyte and non-lymphocyte cells within the region delimited by K3
v193	ratio between the areas of the lymphocyte cells and the tissue area within the region delimited by K1
v194	ratio between the areas of the lymphocyte cells and the tissue area within the region delimited by K2
v195	ratio between the areas of the lymphocyte cells and the tissue area within the region delimited by K3
v196	ratio between the number of lymphocyte and non-lymphocyte cellswithin the region delimited by K1
v197	ratio between the number of lymphocyte and non-lymphocyte cells within the region delimited by K2
v198	ratio between the number of lymphocyte and non-lymphocyte cells within the region delimited by K3
v199	median of the square difference of the intensities of individual lymphocytes within the region delimited by K1

v200	median of the square difference of the intensities of individual lymphocytes within the region delimited by K2
v201	median of the square difference of the intensities of individual lymphocytes within the region delimited by K3
v202	area enclosed by the non-lymphocyte cells and area enclosed by the lymphocyte cells within the region delimited by K1
v203	area enclosed by the non-lymphocyte cells and area enclosed by the lymphocyte cells within the region delimited by K2
v204	area enclosed by the non-lymphocyte cells and area enclosed by the lymphocyte cells within the region delimited by K3
v205	intersected segment between the area enclosed by the non-lymphocyte cells and area enclosed by the lymphocyte cells within the region delimited by K1
v206	intersected segment between the area enclosed by the non-lymphocyte cells and area enclosed by the lymphocyte cells within the region delimited by K2
v207	intersected segment between the area enclosed by the non-lymphocyte cells and area enclosed by the lymphocyte cells within the region delimited by K3
v208	median of the distances between the lymphocyte cells and their closest non-lymphocyte cells within the region delimited by K1
v209	median of the distances between the lymphocyte cells and their closest non-lymphocyte cells within the region delimited by K2
v210	median of the distances between the lymphocyte cells and their closest non-lymphocyte cells within the region delimited by K3
v211	ratio between the median of the minimum distance between the lymphocyte cells, and the median of the minimum distances between the lymphocyte and closest non-lymphocyte cells within the region delimited by K1
v212	ratio between the median of the minimum distance between the lymphocyte cells, and the median of the minimum distances between the lymphocyte and closest non-lymphocyte cells within the region delimited by K2
v213	ratio between the median of the minimum distance between the lymphocyte cells, and the median of the minimum distances between the lymphocyte and closest non-lymphocyte cells within the region delimited by K3
v214	number of the lymphocyte cells within the area enclosed by the non-lymphocyte cells within the region delimited by K1
v215	number of the lymphocyte cells within the area enclosed by the non-lymphocyte cells within the region delimited by K2
v216	number of the lymphocyte cells within the area enclosed by the non-lymphocyte cells within the region delimited by K3
v217	sum of the distance between the single lymphocyte cell and the non-lymphocyte cells within the region delimited by K1
v218	sum of the distance between the single lymphocyte cell and the non-lymphocyte cells within the region delimited by K2
v219	sum of the distance between the single lymphocyte cell and the non-lymphocyte cells within the region delimited by K3
v220	total tissue area in pixels from the lymphocyte centroid to the region delimited by K1
v221	entropy of the intensities of the area from the lymphocyte centroid to the region delimited by K1
v222	maximum pixel intensity of the area from the lymphocyte centroid to the region delimited by K1

v223	median pixel intensity of the area from the lymphocyte centroid to the region delimited by K1
v224	minimum pixel intensity of the area from the lymphocyte centroid to the region delimited by K1
v225	median pixel intensity of the tissue holding the lymphocyte cells from the lymphocyte-centroid to the region delimited by K1
v226	entropy of the red channel (R) of the tissue holding the lymphocyte cells from the lymphocyte-centroid to the region delimited by K1
v227	ratio between the median value of all the pixels red (R) and blue (B) channel of the connective tissue holding the lymphocyte cells and delimited by K1
v228	ratio between the median value of all the pixel's red (R) and green (G) channel of the connective tissue holding the lymphocyte cells and delimited by K1
v229	Haralick textural feature: angular energy of the second moment from the image representing the connective tissue, characterized as a gray-level co-ocurrance matrix (GLCM) and delimited by K1
v230	Haralick textural feature: contrast from the image representing the connective tissue, characterized as a gray-level co-ocurrance matrix (GLCM) and delimited by K1
v231	Haralick textural feature: correlation from the image representing the connective tissue, characterized as a gray-level co-ocurrance matrix (GLCM) and delimited by K1
v232	Haralick textural feature: variance from the image representing the connective tissue, characterized as a gray-level co-ocurrance matrix (GLCM) and delimited by K1
v233	Haralick textural feature: inverse different moment from the image representing the connective tissue, characterized as a gray-level co-ocurrance matrix (GLCM) and delimited by K1
v234	Haralick textural feature: sum of the distinct gray levels in the quantified image representing the connective tissue, characterized as a gray-level co-ocurrance matrix (GLCM) and delimited by K1
v235	Haralick textural feature: variance of the distinct gray levels in the quantified image representing the connective tissue, characterized as a gray-level co-ocurrance matrix (GLCM) and delimited by K1
v236	Haralick textural feature: sum of the distinct entropy of the gray levels in the quantified image representing the connective tissue, characterized as a gray-level co-ocurrance matrix (GLCM) and delimited by K1
v237	Haralick textural feature: entropy of the the distinct gray levels in the quantified image representing the connective tissue, characterized as a gray-level co-ocurrance matrix (GLCM) and delimited by K1
v238	Haralick textural feature: difference between the rows and columns variances of the distinct gray levels in the quantified image representing the connective tissue, characterized as a gray-level co-ocurrance matrix (GLCM) and delimited by K1
v239	Haralick textural feature: difference between the rows and columns entropy of the distinct gray levels in the quantified image representing the connective tissue, characterized as a gray-level co-ocurrance matrix (GLCM) and delimited by K1
v240	Haralick textural feature: the information measures of correlation from the image representing the connective tissue, characterized as a gray-level co-ocurrance matrix (GLCM) and delimited by K1
v241	Haralick textural feature: the correlation coefficients of the normal distribution generated from the image representing the connective tissue, characterized as a gray-level co-ocurrance matrix (GLCM) and delimited by K1
v242	Haralick textural feature: the probability exponential distribution of the correlation coefficients calculated from the normal distribution generated from the image representing the connective tissue, characterized as a gray-level co-ocurrance matrix (GLCM) and delimited by K1
v243	total area in pixels from the lymphocyte centroid to the region delimited by K2
v244	entropy of the intensities of the area from the lymphocyte centroid to the region delimited by K2

v245	maximum pixel intensity of the area from the lymphocyte centroid to the region delimited by K2
v246	median pixel intensity of the area from the lymphocyte centroid to the region delimited by K2
v247	minimum pixel intensity of the area from the lymphocyte centroid to the region delimited by K2
v248	sampleedgeMedIntensityK1 median pixel intensity of the tissue holding the lymphocyte cells from the lymphocyte-centroid to the region delimited by K2
v249	entropy of the red channel (R) pixels of the tissue holding the lymphocyte cells from the lymphocyte-centroid to the region delimited by K2
v250	ratio between the median value of all the pixels red (R) and blue (B) channel of the connective tissue holding the lymphocyte cells and delimited by K2
v251	ratio between the median value of all the pixel's red (R) and green (G) channel of the connective tissue holding the lymphocyte cells and delimited by K2
v252	Haralick textural feature: angular energy of the second moment from the image representing the connective tissue, characterized as a gray-level co-ocurrance matrix (GLCM) and delimited by K2
v253	Haralick textural feature: contrast from the image representing the connective tissue, characterized as a gray-level co-ocurrance matrix (GLCM) and delimited by K2
v254	Haralick textural feature: correlation from the image representing the connective tissue, characterized as a gray-level co-ocurrance matrix (GLCM) and delimited by K2
v255	Haralick textural feature: variance from the image representing the connective tissue, characterized as a gray-level co-ocurrance matrix (GLCM) and delimited by K2
v256	Haralick textural feature: inverse different moment from the image representing the connective tissue, characterized as a gray-level co-ocurrance matrix (GLCM) and delimited by K2
v257	Haralick textural feature: sum of the distinct gray levels in the quantified image representing the connective tissue, characterized as a gray-level co-ocurrance matrix (GLCM) and delimited by K2
v258	Haralick textural feature: variance of the distinct gray levels in the quantified image representing the connective tissue, characterized as a gray-level co-ocurrance matrix (GLCM) and delimited by K2
v259	Haralick textural feature: sum of the distinct entropy of the gray levels in the quantified image representing the connective tissue, characterized as a gray-level co-ocurrance matrix (GLCM) and delimited by K2
v260	Haralick textural feature: entropy of the the distinct gray levels in the quantified image representing the connective tissue, characterized as a gray-level co-ocurrance matrix (GLCM) and delimited by K2
v261	Haralick textural feature: difference between the rows and columns variances of the distinct gray levels in the quantified image representing the connective tissue, characterized as a gray-level co-ocurrance matrix (GLCM) and delimited by K2
v262	Haralick textural feature: difference between the rows and columns entropy of the distinct gray levels in the quantified image representing the connective tissue, characterized as a gray-level co-ocurrance matrix (GLCM) and delimited by K2
v263	Haralick textural feature: the information measures of correlation from the image representing the connective tissue, characterized as a gray-level co-ocurrance matrix (GLCM) and delimited by K2
v264	Haralick textural feature: the correlation coefficients of the normal distribution generated from the image representing the connective tissue, characterized as a gray-level co-ocurrance matrix (GLCM) and delimited by K2
v265	Haralick textural feature: the probability exponential distribution of the correlation coefficients calculated from the normal distribution generated from the image representing the connective tissue, characterized as a gray-level co-ocurrance matrix (GLCM) and delimited by K2
v266	total area in pixels from the lymphocyte centroid to the region delimited by K3

v267	entropy of the intensities of the area from the lymphocyte centroid to the region delimited by K3
v268	maximum pixel intensity of the area from the lymphocyte centroid to the region delimited by K3
v269	median pixel intensity of the area from the lymphocyte centroid to the region delimited by K3
v270	minimum pixel intensity of the area from the lymphocyte centroid to the region delimited by K3
v271	median pixel intensity of the piece of tissue holding the lymphocyte cells from the lymphocyte-centroid to the region delimited by K3
v272	entropy of the red channel (R) pixels of the tissue holding the lymphocyte cells from the lymphocyte-centroid to the region delimited by K3
v273	ratio between the median value of all the pixels red (R) and blue (B) channel of the connective tissue holding the lymphocyte cells and delimited by K3
v274	ratio between the median value of all the pixel's red (R) and green (G) channel of the connective tissue holding the lymphocyte cells and delimited by K3
v275	Haralick textural feature: angular energy of the second moment from the image representing the connective tissue, characterized as a gray-level co-ocurrance matrix (GLCM) and delimited by K3
v276	Haralick textural feature: contrast from the image representing the connective tissue, characterized as a gray-level co-ocurrance matrix (GLCM) and delimited by K3
v277	Haralick textural feature: correlation from the image representing the connective tissue, characterized as a gray-level co-ocurrance matrix (GLCM) and delimited by K3
v278	Haralick textural feature: variance from the image representing the connective tissue, characterized as a gray-level co-ocurrance matrix (GLCM) and delimited by K3
v279	Haralick textural feature: inverse different moment from the image representing the connective tissue, characterized as a gray-level co-ocurrance matrix (GLCM) and delimited by K3
v280	Haralick textural feature: sum of the distinct gray levels in the quantified image representing the connective tissue, characterized as a gray-level co-ocurrance matrix (GLCM) and delimited by K3
v281	Haralick textural feature: variance of the distinct gray levels in the quantified image representing the connective tissue, characterized as a gray-level co-ocurrance matrix (GLCM) and delimited by K3
v282	Haralick textural feature: sum of the distinct entropy of the gray levels in the quantified image representing the connective tissue, characterized as a gray-level co-ocurrance matrix (GLCM) and delimited by K3
v283	Haralick textural feature: entropy of the the distinct gray levels in the quantified image representing the connective tissue, characterized as a gray-level co-ocurrance matrix (GLCM) and delimited by K3
v284	Haralick textural feature: difference between the rows and columns variances of the distinct gray levels in the quantified image representing the connective tissue, characterized as a gray-level co-ocurrance matrix (GLCM) and delimited by K3
v285	Haralick textural feature: difference between the rows and columns entropy of the distinct gray levels in the quantified image representing the connective tissue, characterized as a gray-level co-ocurrance matrix (GLCM) and delimited by K3
v286	Haralick textural feature: the information measures of correlation from the image representing the connective tissue, characterized as a gray-level co-ocurrance matrix (GLCM) and delimited by K3
v287	Haralick textural feature: the correlation coefficients of the normal distribution generated from the image representing the connective tissue, characterized as a gray-level co-ocurrance matrix (GLCM) and delimited by K3
v288	Haralick textural feature: the probability exponential distribution of the correlation coefficients calculated from the normal distribution generated from the image representing the connective tissue, characterized as a gray-level co-ocurrance matrix (GLCM) and delimited by K3

Supplementary Table 5. statistical analysis of the cluster conformation between lung ad and scc

(a) ad and scc comparison based on individual clusters

**ad vs scc**	**t-value**	**df 95 % CI**	**p-value**
**C1**	4.22	3802 (0.028,0.076)	2.60E-05
C2	0.79	434.67 (-0.061,0.141)	0.44
C3	1.02	549.85 (-0.034,0.11)	0.31
C4	1.12	5.98 (-0.70,2.03)	0.28
**C5**	3.57	67 (0.09,0.32)	6.60E-04
**C6**	6.96	388.31 (0.33,0.59)	1.46E-11
C7	-0.71	58.42 (-0.18,0.09)	0.48
C8	-0.1	196.1 (-0.01,0.06)	0.7

(b) ad and scc differences

**ad vs scc**	**chi-squared**	**df 95 % CI**	**p-value**
**ad**	172.67	7	2.20E-16
**scc**	25.07	7	8.00E-04

(c) TIL subtype composition between ad and scc

**ad vs scc**	**chi-squared**	**df 95 % CI**	**p-value**
**CD4+**	2.37	617.26 (0.07,0.6)	0.02
**CD8+**	8.95	4321.7 (0.39,0.60)	2.20E-16
CD20+	0.72	73.61 (-0.41,0.88)	0.47

Supplementary Figure 1. Representative images used in the study. **(A)** Image representation for the tumor-microarray (TMA) of Hematoxylin & eosin (H&E) and immunofluorescence (IF) scans. **(B)** Adaptation of the whole-slide image (WSI) to TMA-sized patch extraction from the isolated tumor region, segmented by a deep learning model. From the tumor regions, nuclei were detected and lymphocyte and non-lymphocytes were identified. The identified lymphocytes (blue) and the non-lymphocytes cell (yellow) are seen in the lower right corner. **(C)** KM plots for the TIL cluster model (M^{AD}) across the different stages of LUAD IA, **(D)** IB, **(E)** IIA, **(F)** IIB, **(G)** IIIA, **(H)** IIIB.

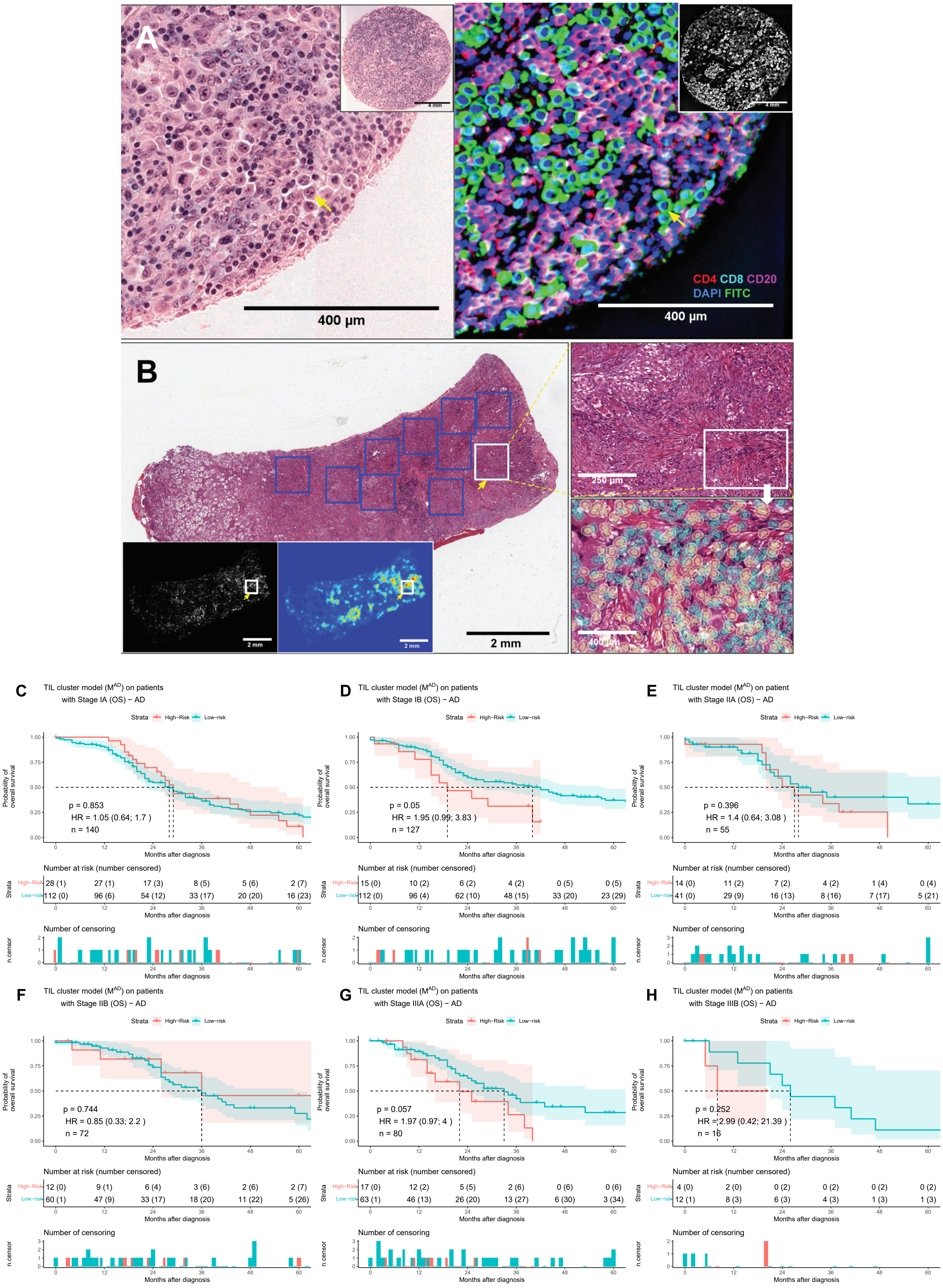

Supplementary Figure 1

Supplementary Figure 2. **(A)** KM plots for the TIL cluster model (M^{AD}) across the different stages, showcased in terms of less granular stage groupings as Stage I, **(B)** II, **(C)** III, **(D)** IV. **(E)** Further shown as early (IA-IIB) and **(F)** late stages (IIIA-IV). **(G)** KM plots for TIL cluster model M^{AD} applied to D_3 to D_5 lung SCC patients and **(H)** TIL cluster model M^{SCC} applied to D_3 to D_5 LUAD patients. **(I)** KM plots for TIL cluster model (M^{AD}) across the different arms of chemotherapy agents used on patients from cohorts D_6 and D_7. Docetaxel/cisplatin or carboplatin, **(J)** carboplatin only, **(K)** Other chemotherapy agents (navelbine, paclitaxel, gemcitabine), **(L)** cisplatin/navelbine docetaxel and pemetrexed combinations. **(M)** TIL cluster model (M^{AD}) applied to D_8 of lung AD treated with monotherapy, Docetaxel.

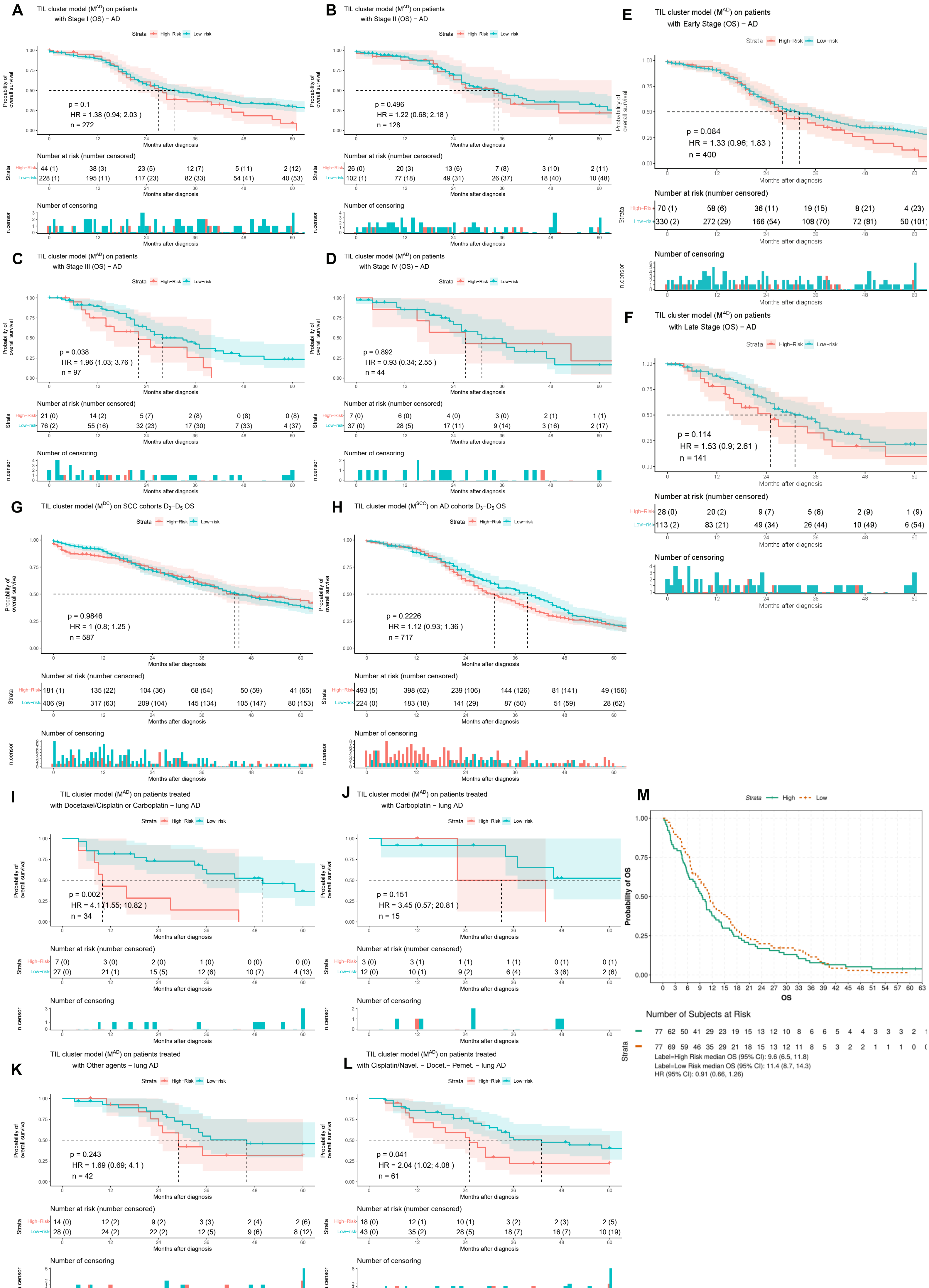

Supplementary Figure 2

Supplementary Figure 3. KM plots using OS as endpoint for the TIL cluster model (A) M^{AD} applied to all testing cohorts (D_3, D_4, D_5 and D_7) for AD and cluster model (B) M^{SCC} applied to all testing cohorts (D_3, D_4 and D_5) for SCC. KM curves for the TIL cluster model (M^{SCC}) across the different stages, showcased in terms of less granular stage groupings as Stage I (C), II (D), III (E) and IV (F). (G) Further shown as early (I-II) and (H) late stages (III-IV).

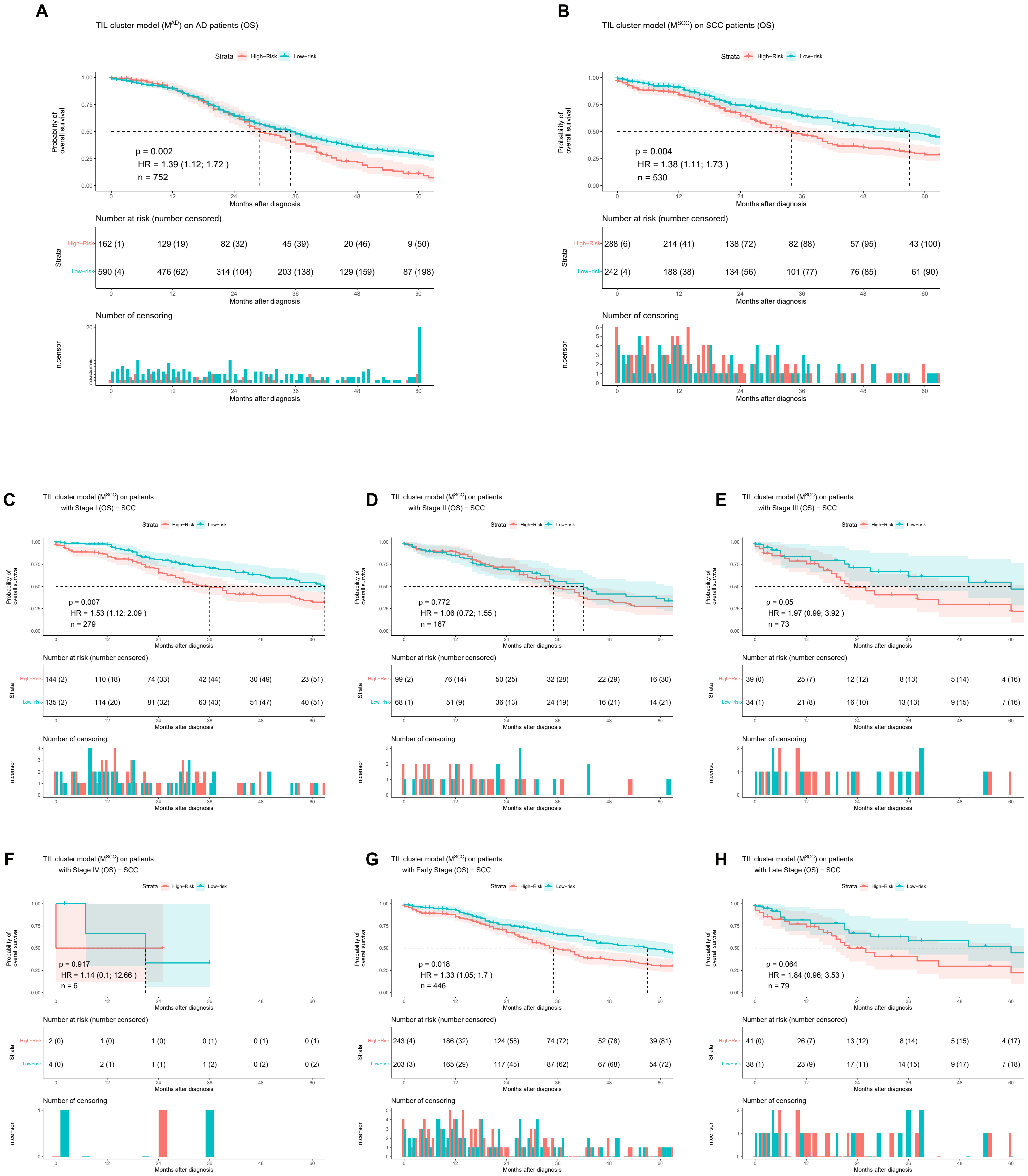

Supplementary Figure 3

Supplementary Figure 4. KM curves for the density of TILs biomarker (denTIL) models applied across the different validation cohorts (D_3 to D_7) for AD ($denTIL^{AD}$ trained on $D^{AD}{}_1$+$D^{AD}{}_2$) and SCC ($denTIL^{SCC}$ trained on $D^{SCC}{}_1$+$D^{SCC}{}_2$), using OS as endpoint. The KM plots can be seen for **(A)** $D^{AD}{}_1$+$D^{AD}{}_2$ (Training set $denTIL^{AD}$) **(B)** D_3, **(C)** D_4, **(D)** D_5, **(E)** D_6, **(F)** D_7. The KM plots can be seen for **(G)** $D^{SCC}{}_1$+$D^{SCC}{}_2$ (Training set $denTIL^{SCC}$) **(H)** D_3, **(C)** D_4, **(D)** D_5.

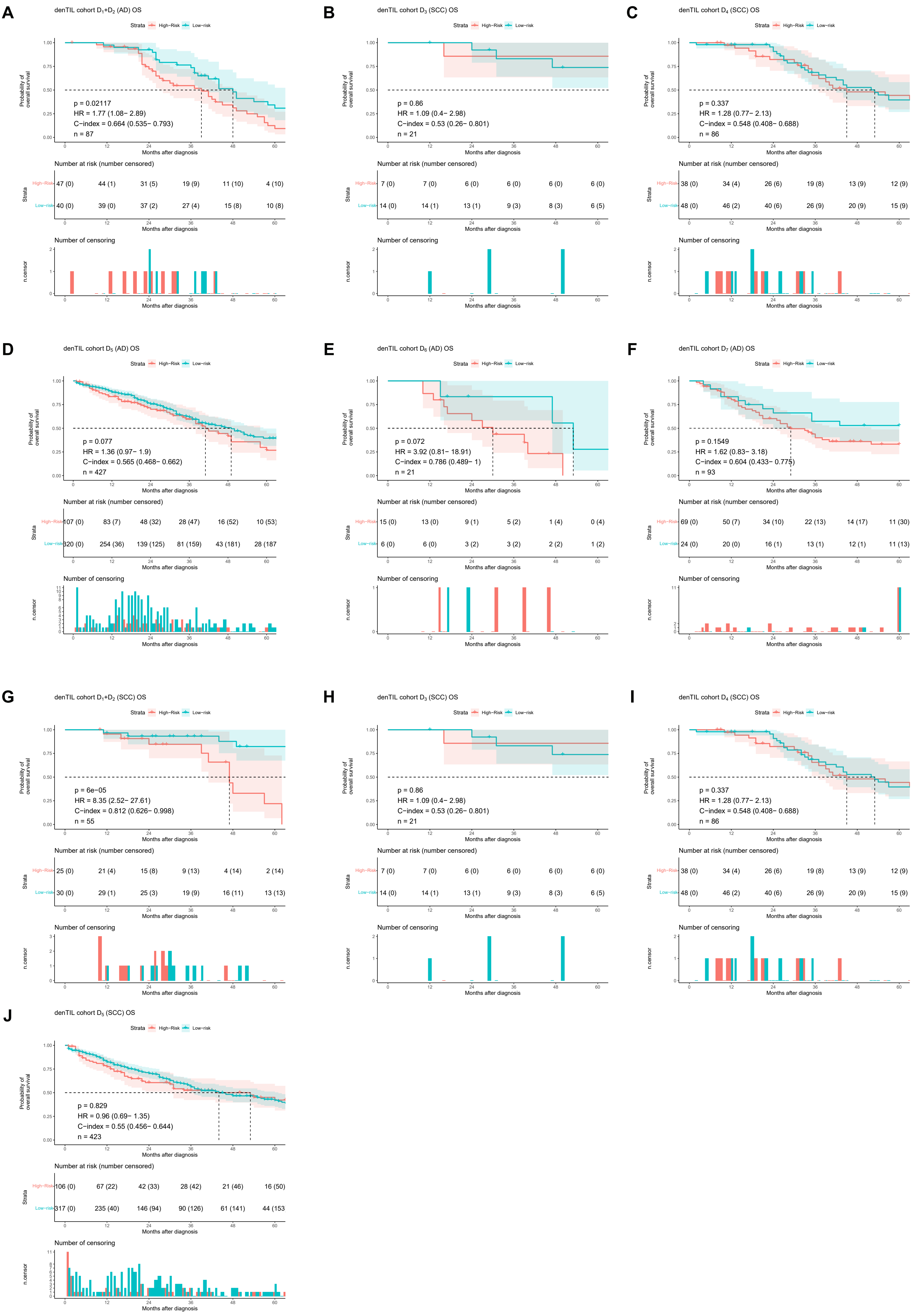

Supplementary Figure 4

Supplementary Figure 5. KM curves for the density of TILs biomarker (spaTIL) models applied across the different validation cohorts (D_3 to D_7) for AD (spaTIL^{AD} trained on $D^{AD}{}_1$+$D^{AD}{}_2$) and SCC (spaTIL^{SCC} trained on $D^{SCC}{}_1$+$D^{SCC}{}_2$), using OS as endpoint. The KM plots can be seen for **(A)** $D^{AD}{}_1$+$D^{AD}{}_2$ (Training set spaTIL^{AD}) **(B)** D_3, **(C)** D_4, **(D)** D_5, **(E)** D_6, **(F)** D_7. The KM plots can be seen for **(G)** $D^{SCC}{}_1$+$D^{SCC}{}_2$ (Training set spaTIL^{SCC}) **(H)** D_3, **(C)** D_4, **(D)** D_5.

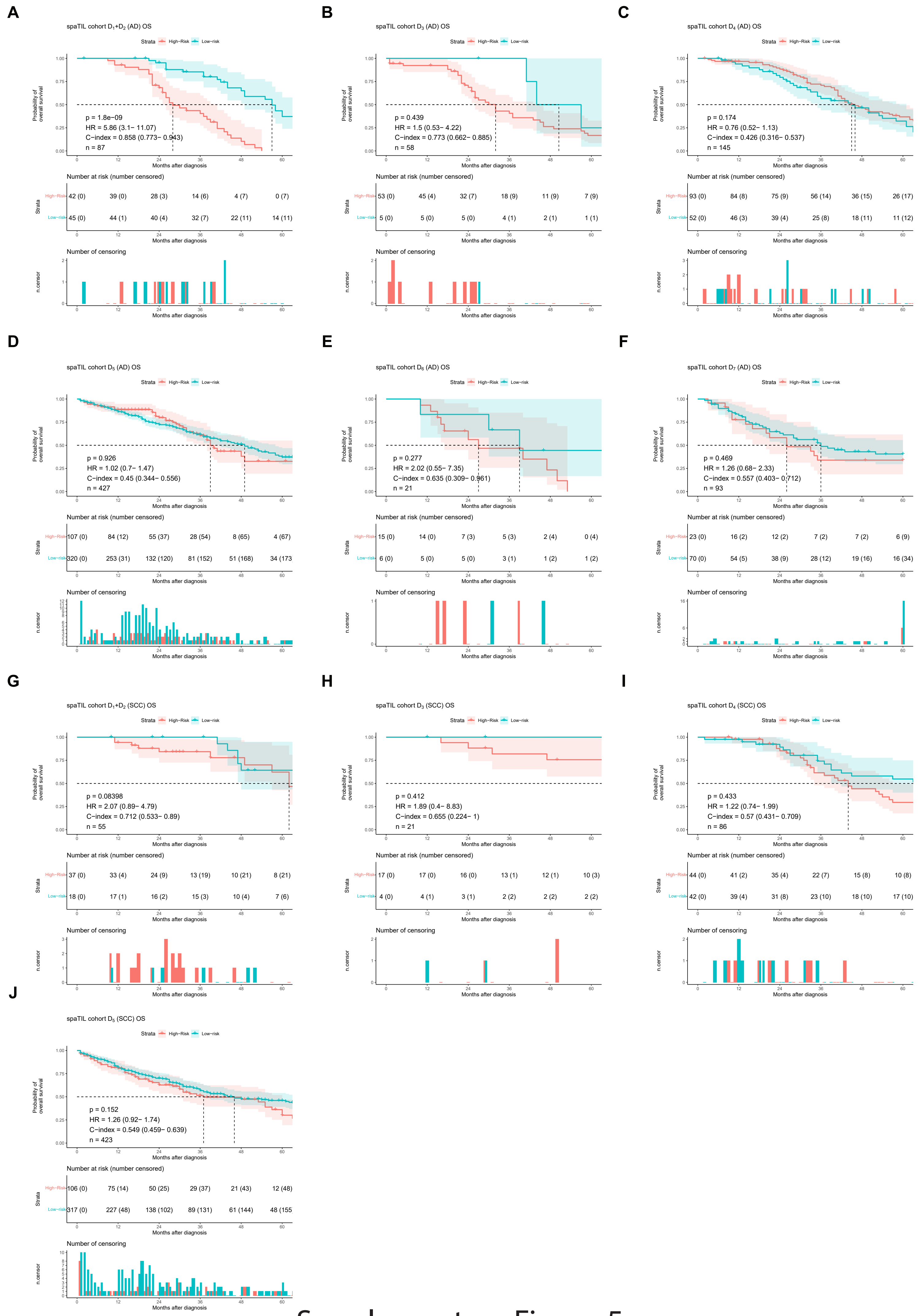

Supplementary Figure 5

Supplementary Figure 6. KM plots for TIL cluster model (M^{AD}) applied to lung AD patients treated with **(A)** radiochemotherapy and **(B)** radiotherapy. **(C)** KM plots for TIL cluster model (M^{AD}) for PD-L1 expression during resection on lung AD patients from cohort D_7, for high PD-L1 (>=50%) and **(D)** low (<50%) PD-L1 expressions, **(E)** positive PD-L1 (>=1%) and **(F)** negative PD-L1 (<1%) expressions.

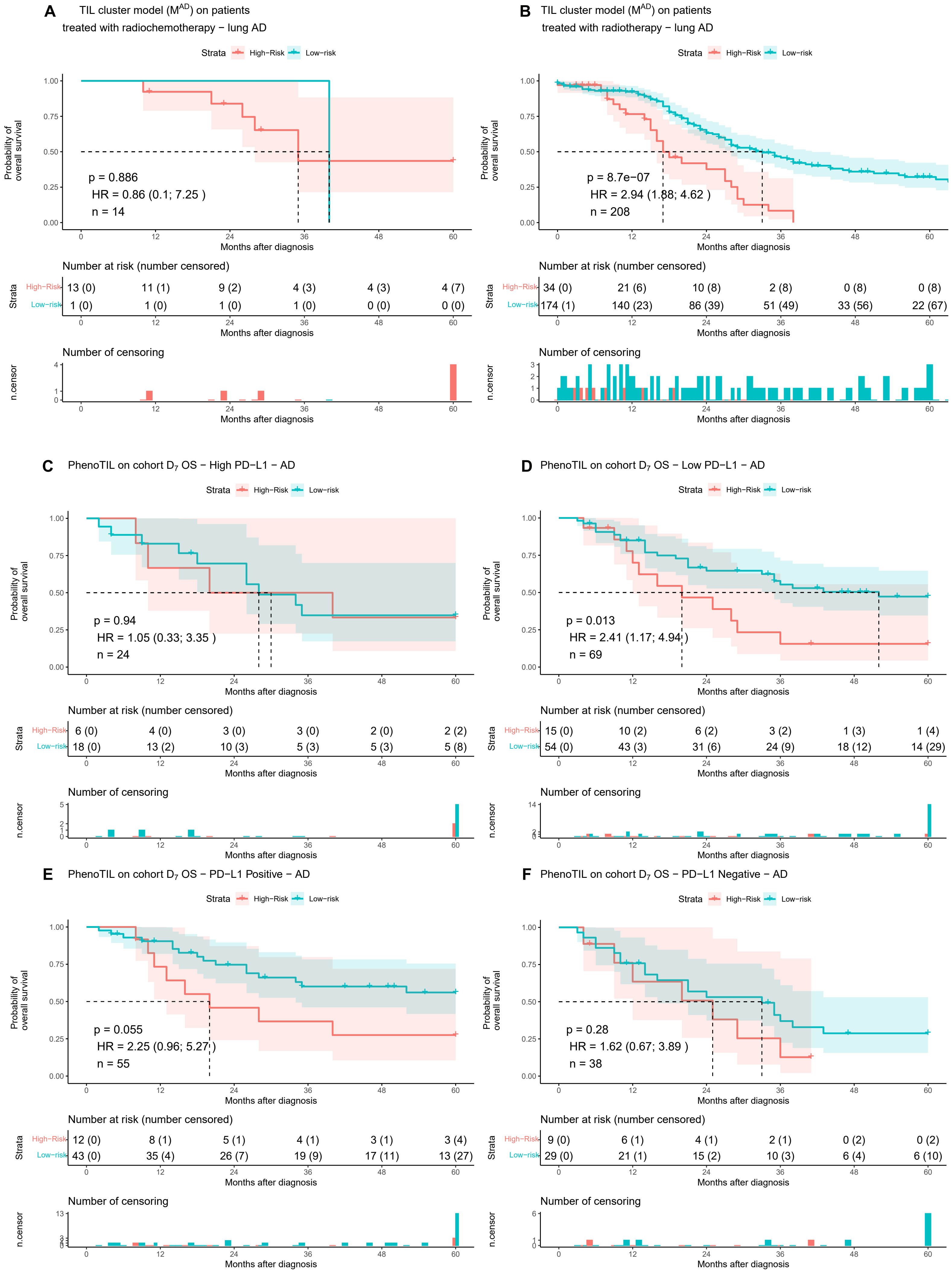

Supplementary Figure 6

Supplementary Figure 7. **(A)** 1,774 patients from eight different sites were embedded into a two-dimensional feature space and then plotted using UMAP. Each point represents a patient and each color a different site. The embedding was done using the 288 PhenoTIL features taken from across the different histologic image tiles and TMAs. The plot indicates that there are no evident clusters of patients based off the individual sites (cohort), suggesting that PhenoTIL features are resilient to batch effects and are consistent across different labs and sites. **(B)** Cluster conformation across LUAD and lung SCC cases from cohort D_3 engendered by the TIL cluster models (M^{AD} and M^{SCC}). The 'constructive' clusters (the cluster that plays a positive association with survival given by the Cox model) for AD and SCC were used for the analysis. On the LUAD samples, the low-risk group has the highest concentration of cluster C1. **(C)** For lung SCC samples, the low-risk group has the highest concentration of cluster C2. The high-risk group has the highest concentration of cluster C8. For the two low-risk group samples, cluster C6 had the highest concentration. For the high-risk group, there was not a clear concentration of a single cluster, but rather a combination of multiple clusters. **(D)** For the TIL subtype, quantity and composition for both LUAD and lung SCC are illustrated. The low-risk group had a higher concentration of $CD8^{+}$. **(E)** TIL cluster quantity and composition for both LUAD and lung SCC are illustrated. The low-risk group was dominated by clusters C1 and C4. The high-risk group was dominated by cluster C7.

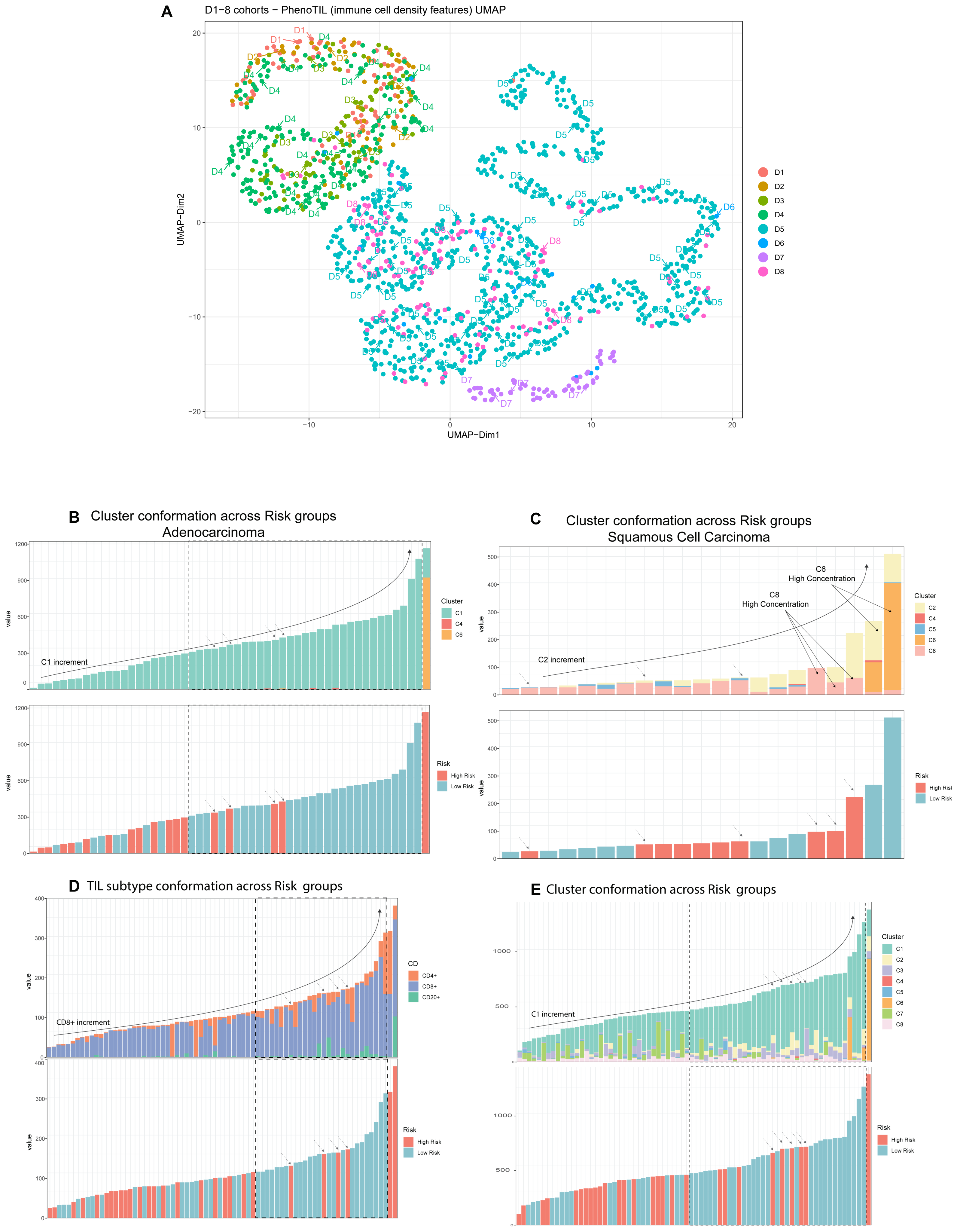

Supplementary Figure 7

Supplementary Figure 8. PhenoTIL analysis for the LUAD model (M^{AD}) is performed using ridgeline plots, a useful chart to visualize feature distributions and compare the low and high-risk groups. For a more detailed explanation, refer to the Supplementary Material, 'PhenoTIL feature description for lung AD and SCC clusters'. Four features distributions are illustrated for clusters in cohort D_3. The ridgeplot was generated for cluster **(A)** C1, **(B)** C2, **(C)** C3, and **(D)** C8. **(E)** PhenoTIL analysis for the lung SCC model (M^{SCC}) is performed using ridgeline plots, a useful chart to visualize feature distribution and compare between the low and high-risk groups. For a more detailed explanation, refer to the Supplementary Material, 'PhenoTIL feature description for lung AD and SCC clusters'. Four features distributions are illustrated for clusters in cohort D_3. The ridgeplot was generated for clusters C1, **(F)** C2, **(G)** C3, and **(H)** C8.

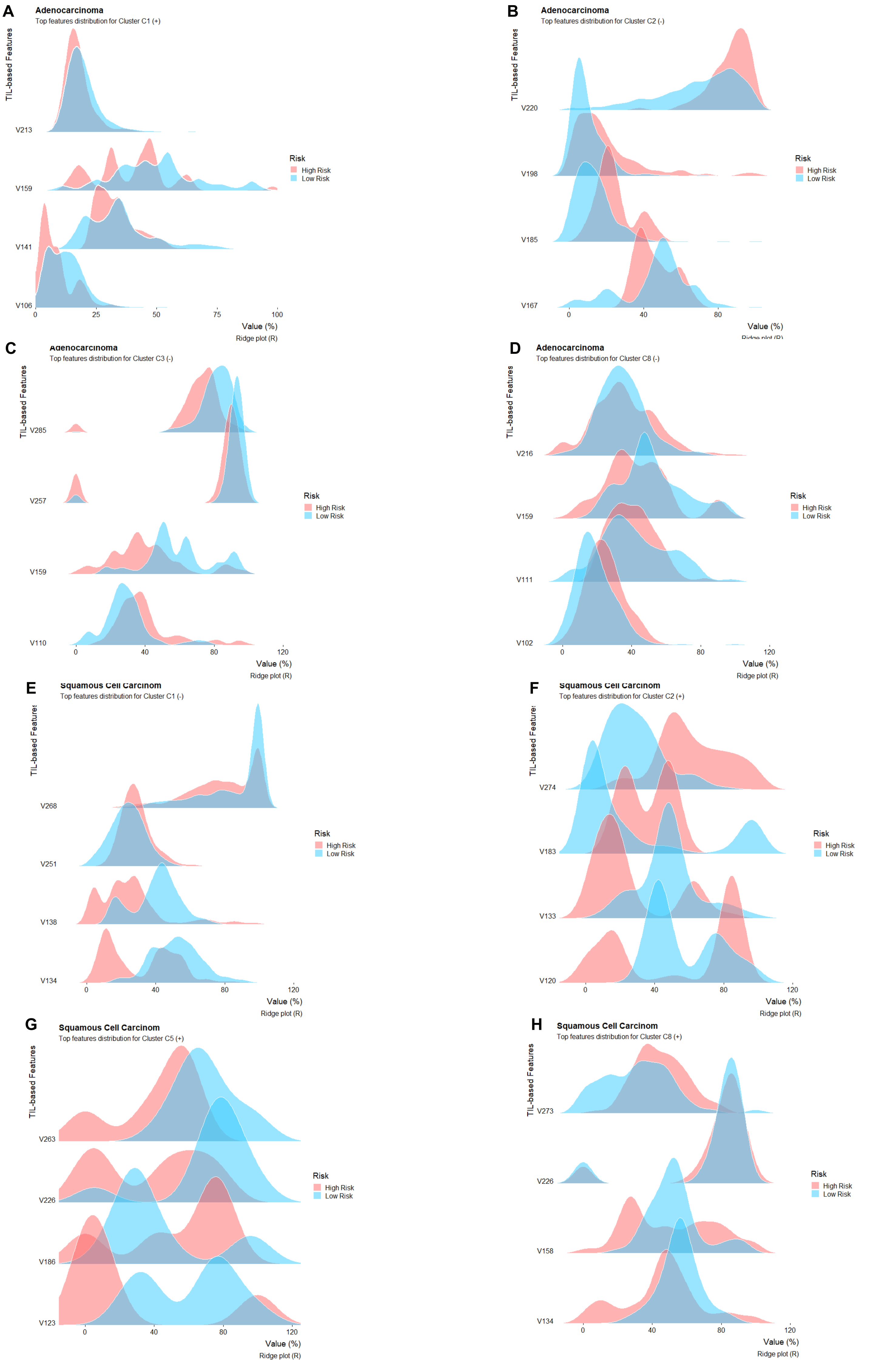

Supplementary Figure 8

Supplementary Figure 9. Gene ontology and signaling pathway analysis. The significant biological processes, cellular components, molecular functions, and signaling pathways are displayed in box plots. The values are displayed on a logarithmic scale (-log10(FDR)). The GO terms are linked together in each section via a red line, highlighting the variation of the FDR adjusted p-values. **(A)** For the PhenoTIL model (M^{AD}) clusters, the association with molecular pathways in LUAD samples was explored for D_5. The number of regulated genes (nRG) associated with the PhenoTIL model (M^{AD}) was found to be 1159. **(B)** For the PhenoTIL model (MS^{CC}) clusters, the association with molecular pathways in LUSC samples was explored for cohort D_5. The nRG associated with the PhenoTIL model (M^{SCC}) was found to be 2710.

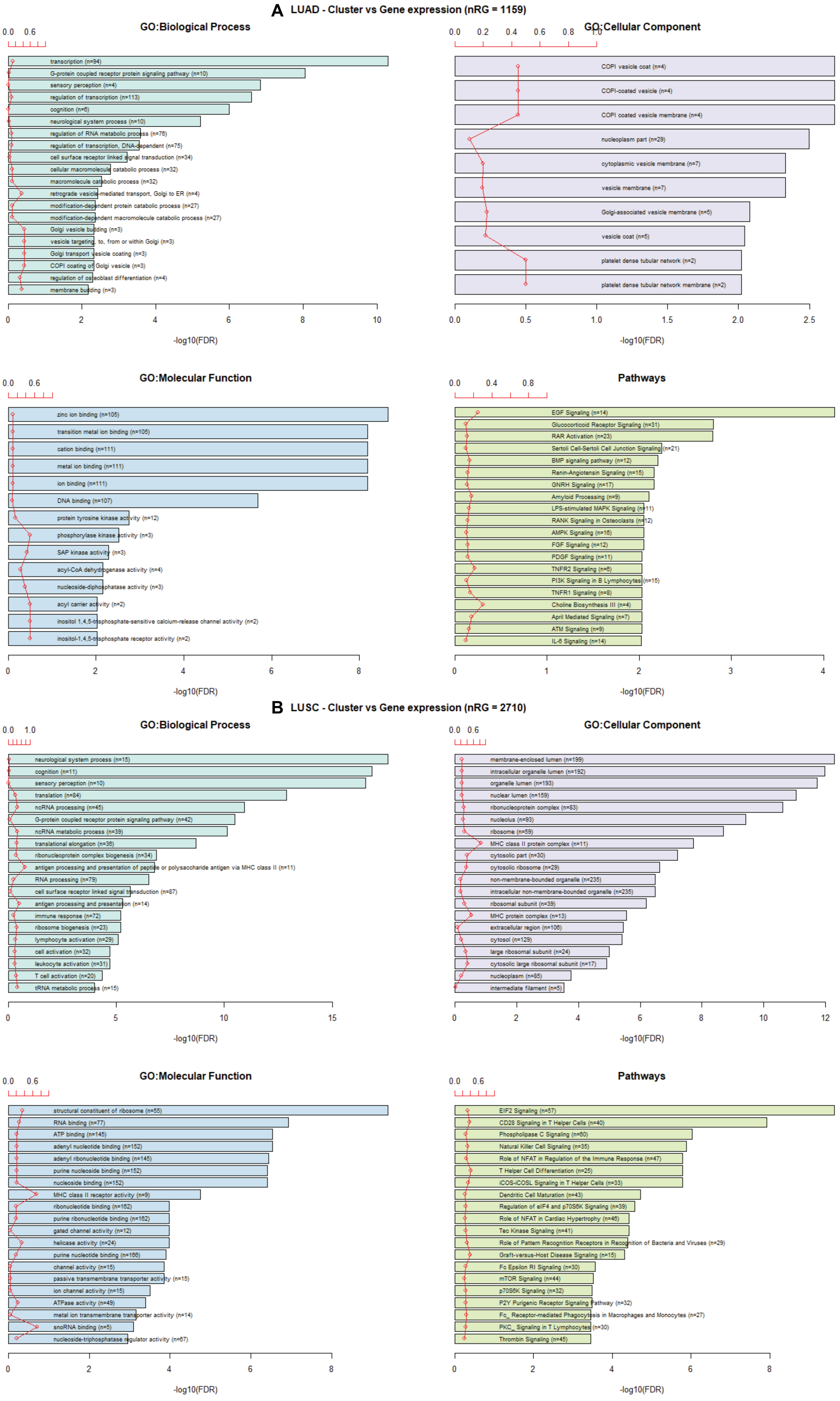

Supplementary Figure 9

Supplementary Figure 10. (A) The PhenoTIL features and the building of the TIL clusters can be initially represented by a process of sampling the neighborhood of lymphocytes and non-lymphocyte cells, including surrounding tumor cells. We use three different regions of interest (K1, K2, K3) to sample the cells. K1 allows fewer cells, compared to K3 which allows for inclusion of the most cells. The distinctive PhenoTIL features are extracted and generated across the three spatial radii. **(B)** The identification of the TIL subtpes with the spatial pairing of the immunofluorescence and H&E images is shown. The process involves the localization of individual lymphocytes on both images, the histogram representation for each of the TIL subtype color staining, representing CD4, CD8 and C20, and the final quantification of pixel-wise staining for each lymphocyte. **(C)** A TMA is shown, highlighting the identified area of interest (with a small red square). The area is shown alongside the detected binary mask for the nuclei, the color heatmap for the TIL subtype stains of CD4, CD8, CD20 and also the epithelial channel (cytokeratine). The process involves the overlaying of the binary mask over each of different stains and the quantification is performed (after pre-processing and adjustments on account of image registration). The histogram of each channel for the lymphocyte cells is generated. **(D)** The membrane and cytoplasm are further identified and the pixel intensity from the membrane is quantified for each IF channel ($CD4^+$, $CD8^+$, $CD20^+$). The process is based on the comparison at a pixel level, of the intensities of each cell membrane. The values are then normalized by the AQUA value. The marker with the highest value is taken as the true label for the cell. This is repeated for each detected TIL.

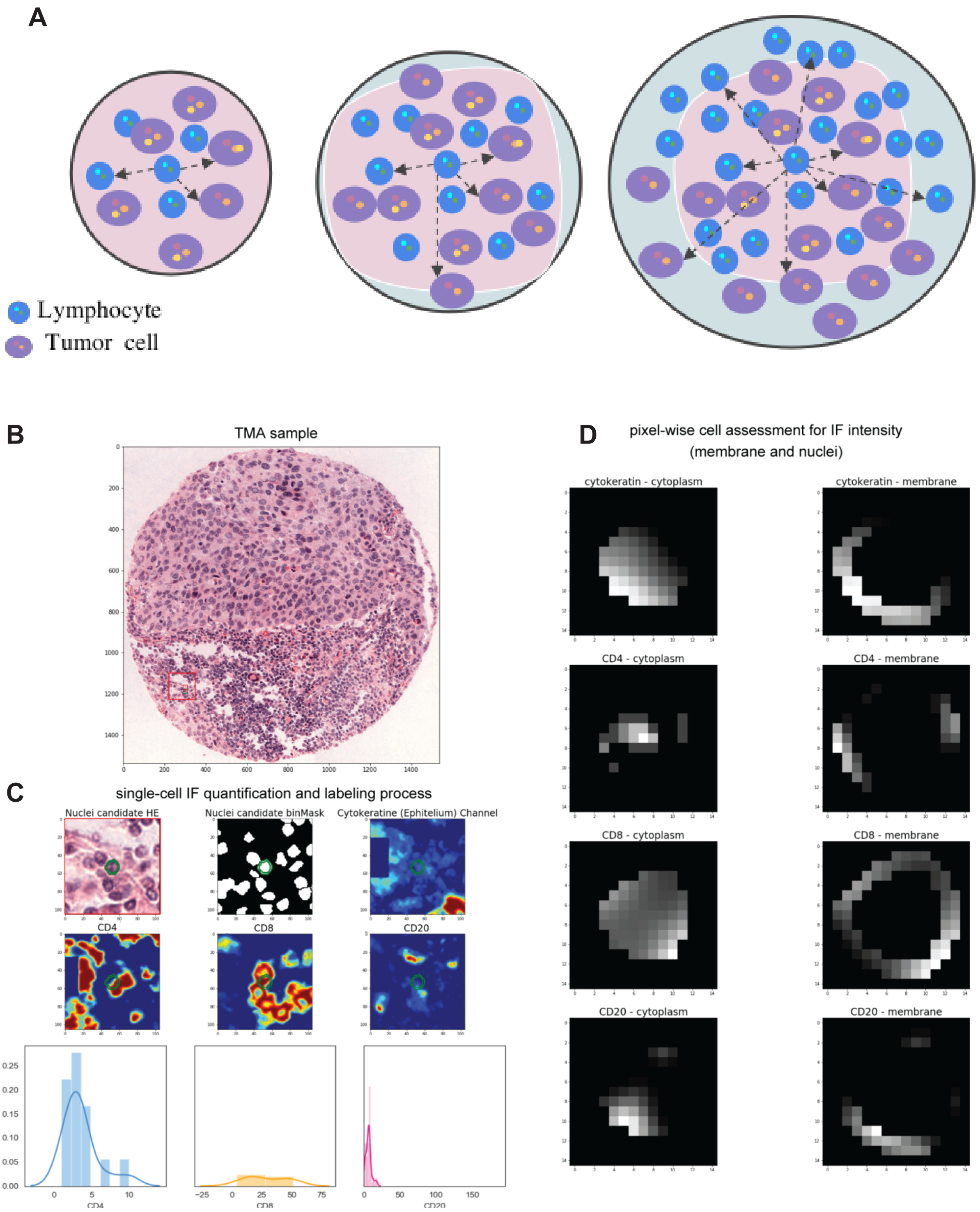

Supplementary Figure 10
